# Mechanical strain stimulates COPII‐dependent secretory trafficking via Rac1

**DOI:** 10.15252/embj.2022110596

**Published:** 2022-08-08

**Authors:** Santosh Phuyal, Elena Djaerff, Anabel‐Lise Le Roux, Martin J Baker, Daniela Fankhauser, Sayyed Jalil Mahdizadeh, Veronika Reiterer, Amirabbas Parizadeh, Edward Felder, Jennifer C Kahlhofer, David Teis, Marcelo G Kazanietz, Stephan Geley, Leif Eriksson, Pere Roca‐Cusachs, Hesso Farhan

**Affiliations:** ^1^ Institute of Basic Medical Sciences University of Oslo Oslo Norway; ^2^ Institute for Bioengineering of Catalonia (IBEC) the Barcelona Institute of Technology (BIST) Barcelona Spain; ^3^ Department of Systems Pharmacology and Translational Therapeutics, Perelman School of Medicine University of Pennsylvania Philadelphia Pennsylvania USA; ^4^ Institute of Pathophysiology Medical University of Innsbruck Innsbruck Austria; ^5^ Department of chemistry and molecular biology University of Gothenburg Gothenburg Sweden; ^6^ Institute of General Physiology University of Ulm Ulm Germany; ^7^ Institute of Cell Biology Medical University of Innsbruck Innsbruck Austria; ^8^ Universitat de Barcelona Barcelona Spain

**Keywords:** COPII, endoplasmic reticulum, mechanobiology

## Abstract

Cells are constantly exposed to various chemical and physical stimuli. While much has been learned about the biochemical factors that regulate secretory trafficking from the endoplasmic reticulum (ER), much less is known about whether and how this trafficking is subject to regulation by mechanical signals. Here, we show that subjecting cells to mechanical strain both induces the formation of ER exit sites (ERES) and accelerates ER‐to‐Golgi trafficking. We found that cells with impaired ERES function were less capable of expanding their surface area when placed under mechanical stress and were more prone to develop plasma membrane defects when subjected to stretching. Thus, coupling of ERES function to mechanotransduction appears to confer resistance of cells to mechanical stress. Furthermore, we show that the coupling of mechanotransduction to ERES formation was mediated via a previously unappreciated ER‐localized pool of the small GTPase Rac1. Mechanistically, we show that Rac1 interacts with the small GTPase Sar1 to drive budding of COPII carriers and stimulates ER‐to‐Golgi transport. This interaction therefore represents an unprecedented link between mechanical strain and export from the ER.

## Graphical abstract

Mechanical strain regulates the number of endoplasmic reticulum exit sites through small GTPase heterodimerization.

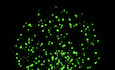

## Introduction

Endoplasmic reticulum (ER) exit sites (ERES) are specialized ribosome‐free domains of the rough ER (Malkus *et al*, 2002; Farhan *et al*, 2007; Shomron *et al*, 2021), which give rise to an intricate network of tubules and vesicles that ferry cargo toward distal compartments (Zeuschner *et al*, 2006; Phuyal & Farhan, 2021; Weigel *et al*, 2021). In recent years, ERES have emerged as platforms that integrate signaling pathways in response to alterations of secretory protein load, starvation, or mitogens (Farhan *et al*, 2008; Farhan *et al*, 2010; Zacharogianni *et al*, 2011; Centonze *et al*, 2019; Centonze & Farhan, 2019; Subramanian *et al*, 2019). Thus, signaling to ERES allows cells to tune the secretory rate to meet the changing requirements during cell growth and proliferation. At ERES, secretory proteins leave the ER via COPII‐dependent carriers. The assembly of the COPII coat is initiated by the small GTPase Sar1, which is activated by its exchange factor Sec12, a transmembrane ER‐resident protein. Recruitment of Sec12 was shown to reconstitute a critical event in the biogenesis of ERES (Maeda *et al*, 2017). Moreover, we showed recently that phosphorylation of Sec12 regulates ERES number (Centonze *et al*, 2019).

Besides intracellular and environmental chemical stimuli, cells are constantly exposed to mechanical stimuli such as substrate stiffness, compression, or tensile forces (Discher *et al*, 2005). It is now well established that such mechanical cues trigger signaling pathways that mediate changes in cell differentiation, proliferation, growth, and survival (Engler *et al*, 2006; Roca‐Cusachs *et al*, 2013; Gudipaty *et al*, 2017; Janmey *et al*, 2020). Most research in the area of mechanobiology has focused on the plasma membrane, the nucleus, or the cytoskeleton as receivers and mediators of mechanical signaling (Phuyal & Baschieri, 2020). However, it is currently poorly understood whether and how mechanical stress has any effect on early secretory pathway compartments such as ERES.

The small GTPase Rac1 is a major regulator of actin cytoskeleton remodeling (Nobes & Hall, 1995). Rac1 has been reported to orchestrate spatially restricted signaling cascades at various subcellular organelles (Payapilly & Malliri, 2018; Phuyal & Farhan, 2019). However, any presence of functionally active Rac1 at the ER remains unexplored. Importantly, previous reports show that Rac1 signals downstream of mechanical cues to regulate cellular proliferation, gene expression, nutrient transport, and epithelial wound healing (Katsumi *et al*, 2002; Kumar *et al*, 2004; Liu *et al*, 2007; Yamane *et al*, 2007; Desai *et al*, 2008; Verma *et al*, 2011; Gould *et al*, 2016). An open question is whether Rac1 signaling regulates the early secretory pathway, and whether mechanical cues act as a stimulus for this process.

In this study, we demonstrate that the early secretory pathway responds to mechanical cues. Our results show that changes in mechanical tension increases ERES number and enhances the rate of ER exit in a manner Rac1‐dependent. We further show that Rac1 regulates ERES formation by interacting with the small GTPase Sar1.

## Results

### Mechanical strain stimulates the early secretory pathway in a Rac1‐dependent manner

**Figure 1:**
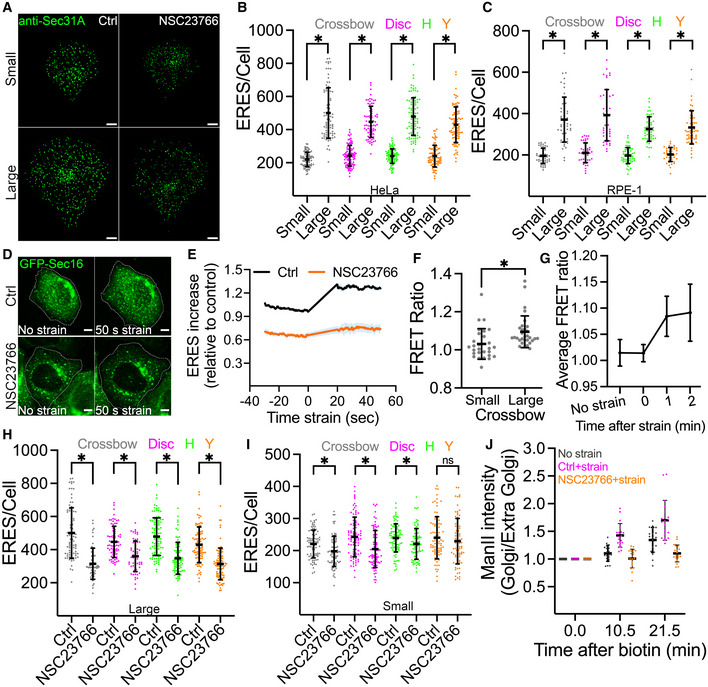
Mechanical strain stimulates ERES and ER‐to‐Golgi transport ARepresentative immunofluorescence images of Sec31A marked ERES in HeLa cells cultured on crossbow shaped micropatterned surface of small (upper panel) or large (lower panel) size for 4 h. Cells were treated with DMSO (Ctrl) or 50 μM NSC23766 (Rac1 inhibitor) for 4 h prior to fixation and immunostaining. Scale bars: 5 μm.B, CThe number of ERES per cell for each pattern size in HeLa (B) and RPE‐1 (C) cells. The number of ERES was quantified from at least 74 cells per condition for HeLa (B) and at least 42 cells per condition for RPE‐1 (C) from three independent experiments. Each dot represents an individual cell.DHeLa cells transiently expressing GFP‐Sec16A were grown on PDMS membranes, treated as indicated and subjected to 7.5% equibiaxial stretch. The effect of stretch on ERES was then monitored by live cell imaging. Representative still images are shown. Scale bar: 5 μm. See also Movie EV1.EQuantification of the experiments in (C). ERES increase in HeLa cells before and during equibiaxial stretch were quantified from a total of 19 cells in four independent live cell imaging experiments. Baseline GFP‐Sec16A count from control (Ctrl) before strain was used to normalize GFP‐Sec16A counts for each condition. Shading represents standard deviation.FHeLa cells transfected with Rac1 FRET biosensor were cultured on crossbow micropatterns and allowed to grow for 4 h before Rac1 FRET ratio was measured in living cells. The dot plot shows FRET ratio for individual cells (small = 28, large = 33 cells) quantified from three independent experiments.GHeLa cells transfected with Rac1 FRET biosensor were cultured on PDMS membranes, subjected to 7.5% equibiaxial stretch and Rac1 FRET ratio was measured in living cells for indicated time points. Graph shows average FRET ratio calculated from a total from 16 cells from three independent experiments.H, IQuantification of ERES in HeLa cells seeded on large (H) and small (I) micropatterned surfaces (described in A). A total of 90–100 cells from three independent experiments were used for ERES quantification.JGolgi arrival kinetics of GFP‐ManII‐RUSH in HeLa cells stably expressing Str‐KDEL‐SBP‐GFP‐ManII (RUSH system). Cell were grown on PDMS membranes, treated as indicated and subjected to 7.5% equibiaxial stretch. See also Movie EV2. ManII arrival at the Golgi was monitored in 19 cells in two independent experiments. See also Movie EV2. Data information: In all graphs, asterisks (*) mark statistical significance (*P*‐value <0.05; Student's unpaired *t*‐test) and error bars show standard deviation.

**Figure EV1:**
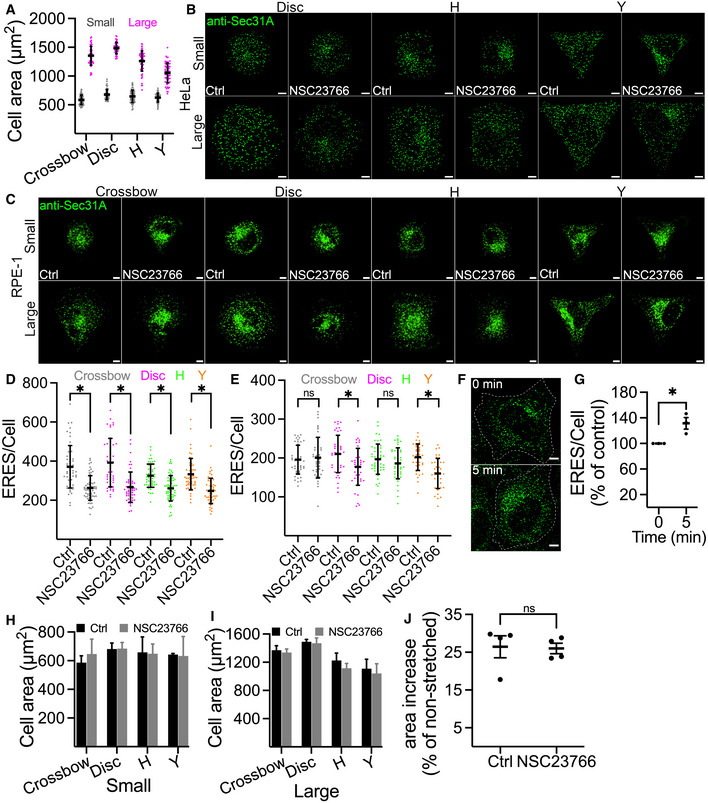
ERES are regulated by an increase in cell area AQuantification graph showing area covered by HeLa cells on Crossbow‐, Disc‐, H‐ or Yshaped fibronectin‐coated micropatterned chips. 50–60 cells from two independent experiments were counted. Error bars show standard deviation, and dots represent individual cells.B, CRepresentative images showing Sec31A labeled ERES in HeLa (B) and RPE‐1 cells (C) seeded on fibronectin‐coated micropatterned chips of indicated geometries and sizes. Cells were treated with either DMSO (Ctrl) or Rac1 inhibitor (NSC23766) for 4 h prior to immunolabeling with Sec31A. Scale bars: 5 μm.D, EQuantification data for (C). Dot plot shows ERES in individual RPE‐1 cells cultured on large (D) and small (E) micropatterned surfaces. At least 42 cells were used for ERES quantification. *n* = 3 (biological replicates), *P‐value <0.05, Student's *t‐*test. Error bars show standard deviation. ns = nonsignificant.F, GHeLa cells were subjected to osmotic shock for the indicated time point and changes in ERES was monitored by counting Sec31A marked ERES. (F) Representative immunofluorescence images showing ERES in control cells and cells subjected to osmotic shock. Scale bar: 5 μm (G) Quantification of ERES in (C). At least 30 cells were counted in each experiment (*n* = 3). Mean ± standard deviation and dots representing average ERES/cell values from each experiment are shown. *P‐value <0.05, Student's *t*‐test.H, IGraphs show area covered by HeLa cells treated with DMSO (Ctrl) or Rac1 inhibitor (NSC23766) grown on small (E) or large (F) fibronectin‐coated micropatterned chips. Between 50 and 60 cells were counted in two experiments. Values show average cell area ± standard deviation.JQuantification graph showing increase in size of HeLa cells during 7.5% biaxial strain. A total of 19 cells were quantified in four live cell imaging experiments. Cell size before strain was used to calculate percent increase in cell size during strain. Mean ± standard deviation and dots representing average values from each experiment are shown. A two‐tailed Student's unpaired *t*‐test was used to determine the statistical significance.

To impose mechanical tension on cells, we cultured HeLa cells on fibronectin‐coated micropatterned surfaces that contained multiple geometric shapes of two different sizes (small: 700 μm^2^, large: 1,600 μm^2^) (Albert & Schwarz, 2014). Cells were allowed to adhere and accommodate to the patterns of different sizes for 4 h followed by fixation and immunostaining with anti‐Sec31 antibodies to label ERES. Strikingly, forcing cells to occupy a larger surface area led to an increased ERES number irrespective of the geometry (Figs 1A left panel and B, and EV1A–B). The same observation was made when we carried out the experiment in nontransformed RPE‐1 cells (Figs 1C and EV1C–E). These findings raised the possibility that ERES respond to mechanical cues. We tested this possibility by subjecting HeLa cells to acute mechanical tension and monitoring ERES in live cells using GFP‐tagged Sec16A as an ERES marker. Cells transiently expressing GFP‐Sec16A were grown on fibronectin‐coated poly(dimethylsiloxane) (PDMS) membranes and subjected to 7.5% biaxial stretch. We noted a rapid increase in the number of ERES in stretched cells within a minute (Fig 1D–E; Movie EV1 upper panel). A similar rapid response was observed by exposing HeLa cells to acute (5 min) mechanical strain through hypotonic swelling (Fig EV1F–G). Thus, mechanical stimulation acutely upregulates ERES number in cells.

We next asked how mechanical strain is linked to the early secretory pathway. Rac1 activation and signaling has been previously linked to mechanical strain (Katsumi *et al*, 2002). To validate that Rac1 is activated in our experimental setup upon mechanical stimulation, we used cells expressing a Rac1 FRET biosensor (Fritz *et al*, 2013). We observed a clear increase in Rac1 activity in cells forced to occupy a large micropatterned surface (Fig 1F) and cells subjected to equibiaxial stretching (Fig 1G). To test whether Rac1 is involved in the stretch‐induced increase in ERES, we cultured cells on the micropatterned surface, allowed them to attach for 30 min and then treated with the Rac1 inhibitor NSC23766 for four hours. NSC23766 is a reversible Rac1‐specific inhibitor that occupies the GEF‐recognition groove centering on Trp56 of Rac1 to inhibit its activation by some GEFs, such as TIAM1 (Gao *et al*, 2004). Rac1 inhibition decreased the number of ERES compared with control in both HeLa and RPE‐1 cells (Figs 1A, H and I, and EV1B–E). Notably, the reduction in ERES number was more pronounced in cells that occupied larger geometries than in the cells occupying smaller geometries (Figs 1A, H and I, and EV1B–E). In a similar manner, NSC23766 also blocked the acute increase in ERES number upon biaxial stretching (Fig 1D and E; Movie EV1). These effects were not due to an effect of NSC23766 on cell surface area, which was found to be similar in treated and untreated cells (Fig EV1H–J). Thus, the increase in ERES number by mechanical stretch is dependent on Rac1.

Because ERES are sites for cargo exit from the ER, we tested whether mechanically challenged cells would exhibit a change in the rate of ER‐to‐Golgi transport. Therefore, we exploited the retention using selective hook (RUSH) assay (Boncompain *et al*, 2012) with Mannosidase II (ManII) as secretory reporter. We applied biaxial strain to HeLa cells stably expressing the RUSH reporter ManII and observed an enhanced ER‐export of ManII in stretched cells compared with that in nonstretched cells (Fig 1J; Movie EV2). Again, Rac1 was essential for stretch‐induced acceleration of ER‐export since perturbation of its activity with the inhibitor NSC23766 delayed the ER‐to‐Golgi transport (Fig 1J; Movie EV2).

Based on the findings from these experiments, we conclude that the early secretory pathway responds to mechanical strain, and this response depends on Rac1 signaling activity.

### The early secretory pathway is required for cellular adaptation to mechanical strain

**Figure 2:**
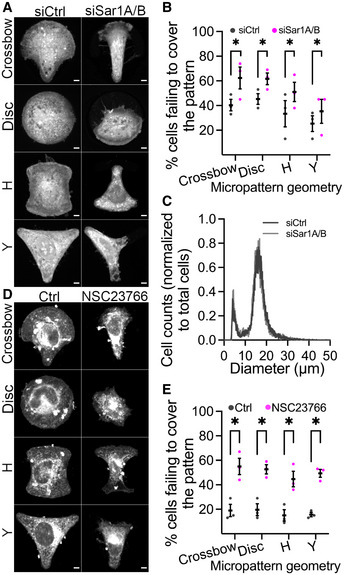
Dysfunctional ERES affects ability of cells to adapt to mechanical tension ARepresentative immunofluorescence images of HeLa cells cultured on micropatterned surface of multiple geometries. Cells were transfected with 10 nM nontargeting control (siCtrl) or cotransfected with siRNAs targeting Sar1A and Sar1B. After 48 h, cells were trypsinized and 80,000 cells were seeded on micropatterned chips. Cells were allowed to attach for 3 h, stained with CellMask for 5 min and processed for microscopy. Scale bars: 5 μm.BQuantification graph showing the percentage of cells failing to entirely cover the different geometries (described in A). Data derived from at least 100 cells from a set of three independent experiments. Error bars represent standard deviation. Asterisks (*) denote statistical significance (*P*‐value <0.05; chi‐squared test).CQuantification graph shows the diameter of cells in nontargeting control (siCtrl) or siRNA targeting Sar1A and Sar1B (siSar1A/B). After trypsinization and resuspension of cells in culture medium, the diameter of cells was determined using the CASY cell counter. A total of 27,630 and 19,677 cells were counted for siCtrl and siSar1A/B, respectively.DRepresentative immunofluorescence images of HeLa cells cultured on micropatterned surface of multiple geometries. Cells were treated with 50 μM of the Rac1 inhibitor NSC23766 for 4 h prior to staining with CellMask and processing for immunofluorescence microscopy. Scale bars: 5 μm.EQuantification of (D). Percentage of cells failing to cover entire micropatterned surface is shown for control (ctrl) and the Rac1 inhibitor NSC23766. Between 113 and 117 cells were counted in total from a set of three independent experiments. Asterisks (*) mark statistical significance (*P*‐value <0.05; chi‐squared test). Error bars show standard deviation.

**Figure EV2:**
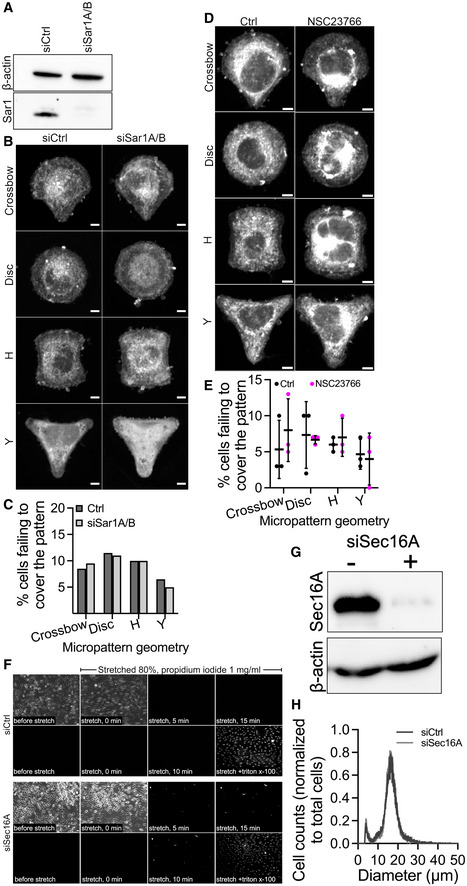
ERES function confers resistance of cell to mechanical stress AHeLa cells were cotransfected with 5 nM siSar1A and 5 nM siSar1B (siSar1A/B) for 48 h. Control cells (siCtrl) were transfected with 10 nM non‐targeting siRNA oligos. The efficiency of knockdown was evaluated by Western blot. A representative immunoblot showing the knockdown efficiency of siRNA oligos.BRepresentative immunofluorescence images of HeLa cells cultured on micropatterned surface of multiple geometries of small size (600 μm2). Cells were treated as described in (A), and after 48 h, cells were trypsinized and 80,000 cells were seeded on micropatterned chips. Cells were allowed to attach for 3 h, stained with CellMask for 5 min, and processed for microscopy. Scale bars: 5 μm.CQuantification graph showing the percentage of cells failing to entirely cover the different geometries (described in B). Between 60 and 70 cells were imaged in two independent experiments.DRepresentative immunofluorescence images of HeLa cells cultured on micropatterned surface of multiple geometries of small size (600 μm2). Cells were treated with DMSO (Ctrl) or NSC23766 for 4 h before staining with CellMask for 5 min and processed for microscopy. Scale bars: 5 μm.EQuantification graph showing the percentage of cells failing to entirely cover the different geometries (described in D). Between 98 and 106 cells were imaged in three independent experiments. Error bars represent standard deviation.FDepletion of Sec16A compromises membrane integrity. HeLa cells were transfected with 5 nM non‐targeting siRNA (Ctrl) or siRNA targeting Sec16A (siSec16A). After 48 h, cells were harvested by trypsinization and immediately seeded on fibronectin‐coated silicon membranes. After 24 h, cells were unidirectionally stretched by 80% for 15 min in the presence of a membrane impermeable dye (propidium iodide). To visualize cells at the end of the experiment, tritonX‐100 was added. Scale bar: 50 μm. Source data are available online for this figure.

Having established the first link between mechanical strain and the early secretory pathway, we examined whether the early secretory pathway has any functional consequences for cellular adaptation during mechanical stimulation. For this purpose, we used small interfering RNA (siRNA) targeting the small GTPases Sar1A and Sar1B, which is known to disrupt the assembly and the functionality of ERES (Cutrona *et al*, 2013). We verified depletion of Sar1 level using Western blot (Fig EV2A). We cultured Sar1‐depleted HeLa cells on the micropatterned surface, allowed them to adhere for 3 h, and monitored whether cells occupy the whole micropattern. We found that a significantly large proportion of Sar1‐depleted cells failed to spread and fully cover the large micropatterns compared with the control cells (Fig 2A and B). On the contrary, no difference between control and Sar1A/B depleted cells was observed for cells on smaller micropattern geometries (Fig EV2B and C). This indicates that the failure to spread and cover the large micropattern is not the result of an adhesion defect, but rather due to an inability of cells to adapt to the mechanical challenge. Of note, the defect in cellular adaptation can also not be attributed to an effect on cell size, which was found to be unaffected by depletion of Sar1A/B (Fig 2C). A similar effect was observed when cells were treated with the Rac1 inhibitor NSC23766 (Fig 2D and E) further supporting a functional link for Rac1 with ERES in the context of adaptation of the cell surface to mechanical stress during cell spreading. As a complementary approach, we tested whether ERES are involved in regulating the ability of cells to ERES in cellular adaptation during mechanical stimulation. We subjected control or Sec16A depleted HeLa cells to mechanical stretch and monitored membrane permeability in live cells using propidium iodide. We found that Sec16A depletion compromises plasma membrane integrity in cells when exposed to mechanical strain (Fig EV2F). This suggests that acute regulation of anterograde membrane flux through the secretory pathway plays a role in maintaining plasma membrane integrity. As with Sar1A/B depletion, Sec16A depletion did not alter cell size (Fig EV2G and H). Altogether, our results support the idea that the early secretory pathway plays an important role during cellular adaptation to mechanical tension.

### Perturbation of Rac1 activation impairs the early secretory pathway

**Figure 3:**
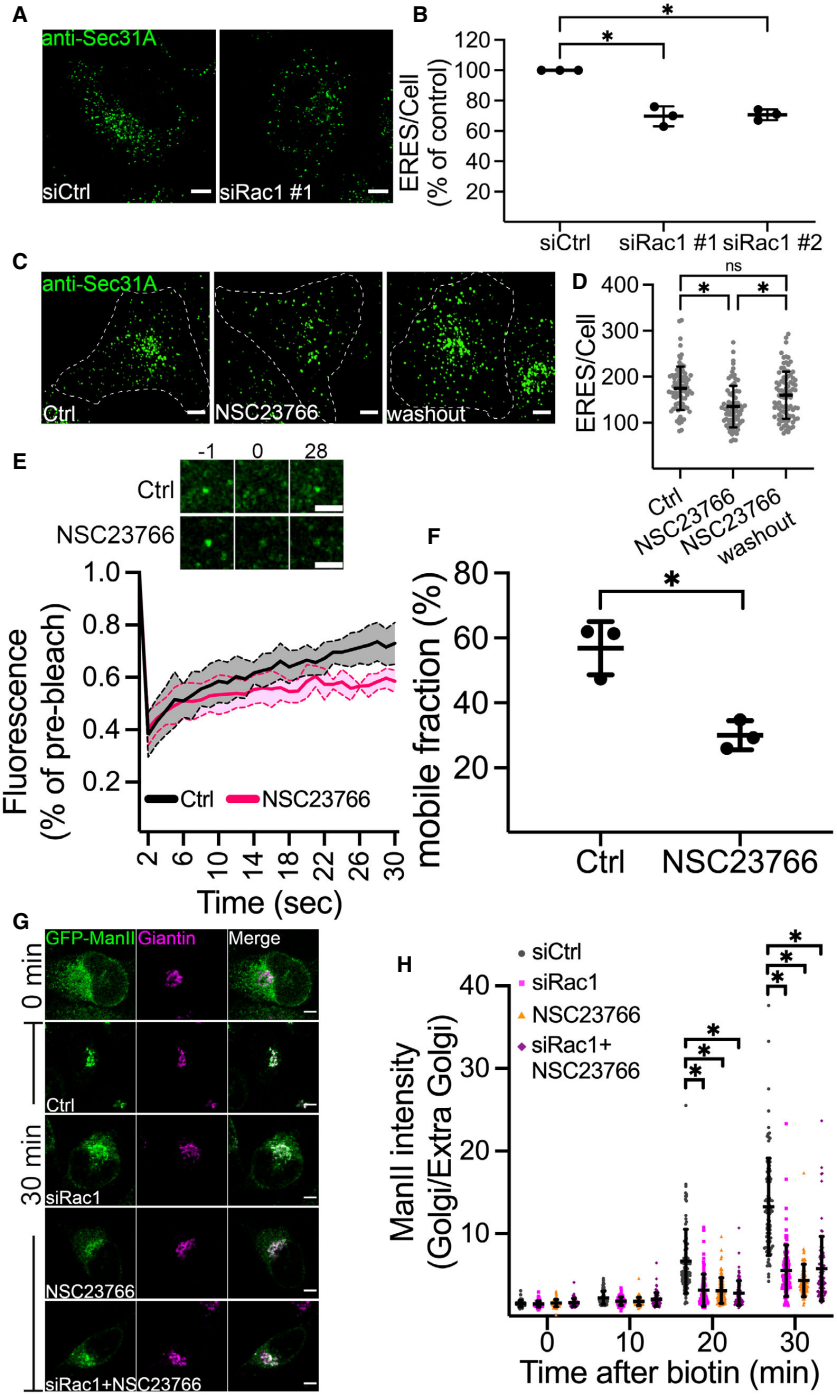
Perturbation of Rac1 activity affects ERES and ER‐export AHeLa cells were transfected with 10 nM nontargeting control siRNA (siCtrl) or siRNAs targeting Rac1 (siRac1). After 72 h, cells were fixed and processed for immunostaining against Sec31A to label ERES. Representative confocal microscopy images are shown.BQuantification graph shows the number of ERES/cell in cells transfected with control siRNA (siCtrl) or siRNA against Rac1 (two siRNAs #1 and #2). Values are expressed as % of siCtrl. From three independent experiments, with >30 cells per condition were counted.CRepresentative immunofluorescence images showing ERES in HeLa cells treated with DMSO (Ctrl), Rac1 inhibitor NSC23766 (50 μM, 4 h), or NSC23766 washout (50 μM for 4 h, then washout for 2 h).DGraph shows the number of ERES per cell (displayed as % of Ctrl) derived from at least 30 cells per condition from three experiments.EFluorescence recovery after photobleaching (FRAP) of GFP‐Sec16A marked ERES in HeLa cells. Images show an individual ERES before (−1), immediately after (0) and 28 s after photobleaching. The graph illustrates FRAP analysis of individual ERES from 23 (for control) and 24 (for NSC23766) regions in three experiments. Line connects individual time points. Data are ±SD.FQuantification of the mobile fraction of GFP‐Sec16A in control and Rac1 inhibited cells. Mobile fraction is derived from a total of 23 (Ctrl) and 24 (NSC23766) individual ERES from three independent experiments.GThe rate of ER‐export was monitored using GFP‐ManII‐RUSH in HeLa cells after perturbation of Rac1 with siRNA (siRac1), or Rac1 inhibitor NSC23766, or a combination of both. Representative images show GFP‐ManII‐RUSH distribution in HeLa cells at indicated time points.HQuantification shows ratio of ManII fluorescence intensity within Golgi to outside Golgi region after addition of biotin at indicated time points. Between 76 and 104 cells were used for measurement of ManII intensity in three experiments. Data information: Scale bars in all immunofluorescence images are 5 μm. Asterisks (*) indicate statistical significance (*P*‐value <0.05), ns indicates nonsignificant. For data presented in (B), (D), and (H), one‐way Anova was used, whereas Student's unpaired *t*‐test was used for F. Error bars represent standard deviation.

**Figure EV3:**
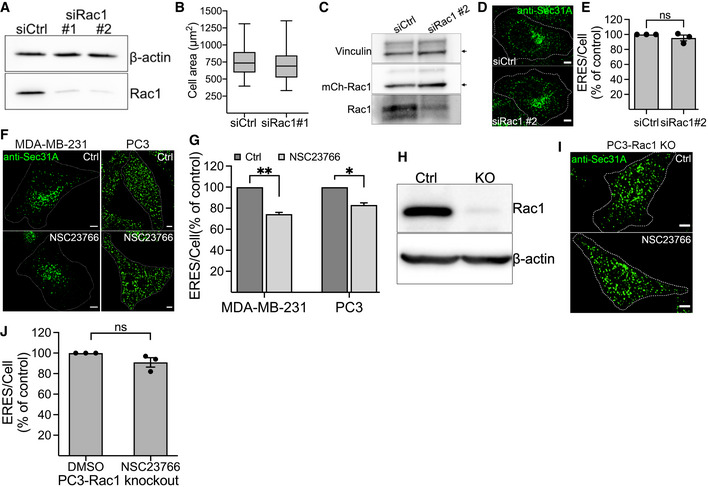
Rac1 regulates ERES AHeLa cells were transfected with two siRNAs against Rac1 (siRac1 #1 and siRac1 #2) for 72 h. The efficiency of knockdown was evaluated by Western blot. A representative immunoblot showing the knockdown efficiency of siRNA oligos.BArea covered by HeLa cells transfected with non‐targeting control siRNA (siCtrl) or siRNA against Rac1 (siRac1 #1). Number of cells = 30, number of experiments = 3. In the boxplot, whiskers indicate the min‐max range of the data and the middle band indicates the median of all data.C–EHeLa cells stably expressing mCherry‐tagged siRNA resistant Rac1 was transfected with control siRNA (siCtrl) or siRNA against Rac1 (siRac1 #2) for 72 h. Cells were then lysed for immunoblotting or processed for immunostaining with Sec31A. (C) A representative immunoblot showing reduction in the levels of endogenous Rac1 while siRNA resistant mCherry‐Rac1 remains unaffected. (D) Immunofluorescence images showing ERES in siCtrl and siRac1 treated cells. Scale bars, 5 μm. (E) Quantitation of experiment in D. Graph shows mean ± SEM (*n* = 3, siCtrl: 117 cells, siRac1 #2: 94 cells). ns = not siginificant.F, GInhibition of Rac1 with NSC23766 (50 μM for 4 h) reduces ERES in MDA‐MB‐231 and PC3 cells. Sec31A marked ERES are shown in F and quantification of ERES is shown in (F). Scale bars: 5 μm. (G) Graph shows mean ± SEM (*n* = 3 experiments, at least 30 cell/experiment), where asterisks (*) represent statistical significance at *P*‐value <0.05, and two asterisks represent statistical significance at *P*‐value <0.05 (Student's unpaired *t* test).HImmunoblot of CRISPR Rac1 knockout PC3 cells.I, JEffect of Rac1 inhibitor NSC23766 is on‐target. CRISPR Rac1 knockout PC3 (PC3‐Rac1 KO) cells were treated with DMSO (Ctrl) or NSC23766 (50 μM) for 4 h and ERES was quantified by counting Sec31A labeled puncta from confocal images. (I) Representative images showing ERES in PC3‐Rac1 KO Ctrl or NSC23766 treated cells. Scale bars, 5 μm. (J) Treating PC3‐Rac1 KO cells with NSC23766 does not reduce ERES. Average ERES per cell are displayed in % of control (*n* = 3, number of cells = 60–90). ns = nonsignificant. Source data are available online for this figure.

We next asked whether Rac1 also signals to ERES in the absence of mechanical strain. For this purpose, we silenced Rac1 expression using siRNA in HeLa cells and assessed the number of ERES. We found that downregulation of Rac1 significantly reduced the ERES count (Fig 3A and B). We verified the depletion of Rac1 by Western blotting (Fig EV3A). Since Rac1 is required to maintain the cytoskeleton, we also measured the area covered by Rac1 knockdown cells and found no significant differences compared with control cells (Fig EV3B). The effect of Rac1 depletion on ERES could be rescued by introducing a siRNA‐resistant version of Rac1 in the cells (Fig EV3C–E). To further verify the results from the knockdown experiments, we treated HeLa cells with the Rac1 inhibitor NSC23766 for 4 h, fixed and processed for quantification of ERES by immunofluorescence and confocal microscopy. In agreement with the results (Fig 3A and B) from knockdown experiments, pharmacological inhibition of Rac1 also led to a remarkable decrease in ERES (Fig 3C and D). Similar results were obtained in breast cancer MDA‐MB‐231 cells and prostate cancer PC3 cells (Fig EV3F and G), indicating that the effect is not cell type specific. Notably, treatment of Rac1‐knockout PC3 cells with NSC23766 did not affect ERES, thus verifying an on‐target effect of the inhibitor on ERES (Fig EV3H–J). Because NSC23766 is a reversible inhibitor, we also checked whether the effect of Rac1 inhibition on ERES subsides following inhibitor washout. Indeed, the number of ERES almost completely recover after 2 h of NSC23766 washout (Fig 3C and D).

To determine the effect of Rac1 on ERES dynamics in living cells, we performed fluorescence recovery after photobleaching (FRAP) in cells expressing GFP‐tagged Sec16A. We have previously used this strategy to uncover effects on ERES biogenesis and maintenance (Farhan *et al*, 2010; Tillmann *et al*, 2015). As shown in Fig 3E and F, inhibition of Rac1 reduced both the fluorescence recovery, resulting in a reduced mobile fraction of GFP‐Sec16A after compared with control cells.

We wanted to further investigate whether the observed effect of perturbed Rac1 activity on ERES translates to altered ER‐to‐Golgi transport. We performed RUSH experiments with ManII as a cargo and noted a substantial delay in the rate of ManII arrival at the Golgi upon knockdown or inhibition of Rac1 (Fig 3G and H). Treatment of Rac1‐depleted HeLa cells with NSC23766 had no cumulative effect on ManII arrival at the Golgi as compared with knockdown or inhibition alone (Fig 3G and H). This observation corroborates the PC3 Rac1 knockout ERES data (Fig EV3I and J) and further indicates the on‐target effect of the inhibitor. Taken together, the results presented so far clearly establish an important role for Rac1 in regulating the ERES and the early secretory pathway.

### Manipulation of Rac1 activity at the ER affects ERES

**Figure 4:**
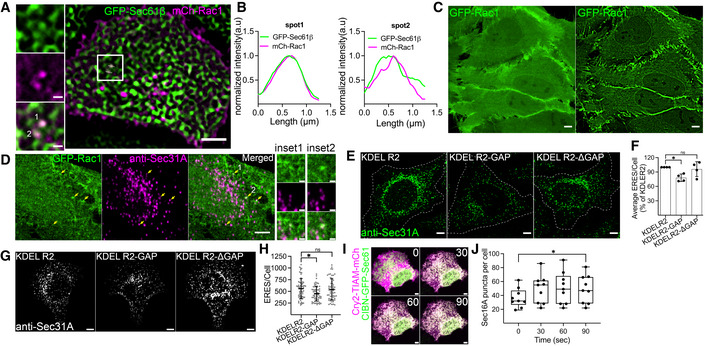
Manipulation of ER‐localized Rac1 affects ERES ACo‐occurrence of mCherry‐Rac1 and GFP‐Sec61β at the endoplasmic reticulum (ER) in HeLa cells stably expressing mCherry‐Rac1 and transiently expressing GFP‐Sec61β as monitored by confocal microscopy. SRRF imaging mode was used in Andor DragonFly spinning disk confocal microscope for obtaining the image. Scale bar: 5 and 1 μm (inset). See also Movie EV3.BLine profile of two marked spots from (A) showing the overlap between mCherry‐Rac1 and GFP‐Sec61β signal at the ER.CSide‐by‐side comparison of cells with endogenously tagged GFP‐Rac1. The image on the left shows a confocal image and the image on the right was performed using SRRF mode. Scale bar: 5 μm.DCells with endogenous GFP‐Rac1 were fixed and stained against Sec31A to label ERES. Arrows indicate Rac1‐puncta colocalizing with ERES. Magnified view of two spots (numbered 1 and 2 in merged channel) is shown. Scale bar: 5 and 1 μm (inset).ERepresentative images showing Sec31A marked ERES in HeLa cells transfected with indicated plasmids. HeLa cells were transfected with the plasmids 16 h before immunolabeling with Sec31A. Scale bar: 5 μm.FQuantification of ERES number in HeLa cells from the experiment described in C. Between 90 and 100 cells were quantified from four independent experiments. Average ERES per cell ± standard deviation is shown. Each dot represents average ERES/cell from an experiment. A one‐way ANOVA was used test for statistical significance. *P‐value <0.05, ns = nonsignificant.GImages represent ERES in HeLa cells that were transfected with the indicated plasmids and cultured on the crossbow micropattern for 4 h. Cells were transfected with the plasmids 16 h before they were trypsinized and seeded on the micropatterns for ERES staining. Scale bar: 5 μm.HThe dot plot shows ERES count in individual cells from (G). Mean ± standard deviation is shown for each condition. At least 58 cells were used for counting ERES number. Independent experiments were performed three times. Statistical significance was tested using one‐way ANOVA, *P‐value <0.05, ns = nonsignificant.IMerged confocal images show distribution of Rac1‐specific GEF TIAM (magenta) in HeLa cells before (0), and after optogenetic recruitment at indicated time points. CIBN‐Sec61β (green) was used to recruit Cry2‐TIAM to the ER with 488 nm laser. Scale bar: 5 μm.JGraph shows the number of SNAP‐Sec16A marked ERES in HeLa cells before (0) and after (30, 60, and 90 s) recruitment of TIAM to the ER from two independent experiments. Whiskers indicate min‐max range and central band indicates the median of all data. Number of cells = 9. A paired *t*‐test was run to compare t0 and t90. *P‐value <0.05.

**Figure EV4:**
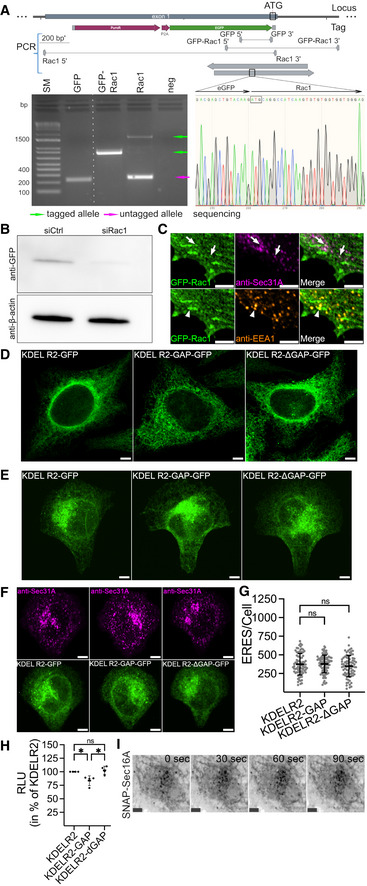
Activity of Rac1 at the ER regulates ERES ACRISPR/Cas system was used to endogenously tag Rac1 in HeLa with GFP. PCR and Sanger sequencing approaches were used to validate successful incorporation of GFP at the desired location. Schematic illustration of GFP knock‐in strategy and binding sites for primers (top). DNA agarose gel showing PCR fragments (bottom left) and sequencing chromatogram showing sequencing results (bottom right).BHeLa cells generated in (A) were transiently transfected with 10 nM siRNA targeting Rac1 (siRac1) for 72 h prior to cell lysis and anti‐GFP immunoblotting. Control (siCtrl) cells were transfected with 10 nM nontargeting siRNA oligos. A representative immunoblot showing levels of GFP‐Rac1 and β‐actin is shown.CHeLa cells generated in (A) were co‐immunostained with anti‐Sec31A and anti‐EEA1 antibodies. Representative immunofluorescence image is shown. Arrows indicate co‐occurrence of GFP‐Rac1 and Sec31A. Note the absence of anti‐EEA1 staining. Arrowhead shows co‐occurrence of GFP‐Rac1 and EEA1. Scale bars, 5 μm.D, ERepresentative immunofluorescence images show expression of the indicated plasmids in HeLa cells cultured on normal glass coverslips (D) or crossbow micropatterns (E). Scale bars, 5 μm.FHeLa cells were transfected with KDELR2‐GFP, KDELR2‐GAP‐GFP or KDELR2‐ΔGAP‐GFP plasmids and incubated overnight. At the end of the incubation, cells were harvested by trypsinization and 80,000 cells were cultured on fibronectin‐coated micropatterns. Cells were allowed to attach for 4 h before fixation and Sec31A immunostaining. Representative immunofluorescence images showing Sec31A marked ERES (upper panel) and plasmid expression (lower panel) in cells covering small crossbow geometry. Scale bars, 5 μm.GQuantification of (F). ERES in 79–91 cells were counted in three experiments. ns = nonsignificant, one‐way ANOVA was used to test for statisticial significance. Error bars show standard deviation.HQuantification graph shows relative luminescence values obtained from HeLa cells that stably express KDELR2‐mCherry, KDELR2‐GAP‐mCherry or KDELR2‐ΔGAP‐mCherry. Cells were lysed and the level of active Rac1 was determined using Rac1 G‐LISA Activation Assay kit following the manufacturer's protocol. Luminescence values obtained from four independent experiments are presented as % of KDELR2‐mCherry (KDELR2) expressing cells. *P‐value <0.05, one‐way ANOVA. ns = nonsignificant. Error bars show standard deviation.ISNAP‐Sec16A distribution in HeLa cells at indicated time points after light‐induced recruitment of TIAM to the ER (see Fig 4I and J). Scale bar: 5 μm. Source data are available online for this figure.

Recent studies have uncovered that multiple and distinct subcellular pools of Rac1 regulate different cellular functions (Phuyal & Farhan, 2019). However, any presence of functionally active Rac1 at the ER remains unexplored. Therefore, we wondered whether Rac1 localizes to the ER. Because Rac1 antibodies do not work in immunofluorescence, we generated HeLa cells stably expressing mCherry‐tagged Rac1. These cells were transiently transfected with GFP‐tagged ER‐resident protein Sec61β to label the ER. Using live cell imaging, we monitored for any co‐occurrence of Rac1 and Sec61β. Because the majority of Rac1 is cytosolic, which could mask a subtle pool of this GTPase at the ER, we used SRRF imaging (Gustafsson *et al*, 2016) to increase the resolution. The localization of Rac1 at the ER might be transient and therefore difficult to detect, because Rac1 predominantly localizes to the plasma membrane and the cytosol. Using SRRF imaging, we detected a transient pool of Rac1 puncta co‐occurring with Sec61β (Fig 4A and B; Movie EV3). To substantiate these findings, we used CRISPR/Cas to endogenously tag Rac1 gene at the N‐terminus with eGFP in HeLa cells. First, we validated successful incorporation of GFP using several approaches (Fig EV4A and B). Next, we performed SRRF imaging with endogenous GFP‐tagged Rac1 HeLa cells, and detected Rac1 on the nuclear envelope (Fig 4C). This is a possible indication of Rac1 at the ER. We also noticed the presence of GFP‐Rac1 puncta, which costained for the ERES marker Sec31A (Fig 4D). Thus, we provide the first evidence for the presence of Rac1 at the ER and at ERES. As previously reported (Palamidessi *et al*, 2008), we also detected a fraction of GFP‐Rac1 at the endosomes (Fig EV4C), but the endosomal pool of Rac1 was distinct from the ERES localized pool (Fig EV4C).

Because we observed ER‐localized pool of Rac1, we next explored whether selective manipulation of this pool would lead to altered ERES. To selectively inactivate Rac1 at the ER, we generated a plasmid expressing the GAP‐domain of the Rac1‐specific GAP β2‐Chimaerin fused to the KDEL receptor 2 (KDELR2) for ER targeting (hereafter KDELR2‐GAP). As a control, we created catalytically inactive GAP fused to KDELR2 by mutating the R363 to alanine (hereafter KDELR2‐ΔGAP). We included KDELR2 as an additional control in our experiments. We overexpressed these plasmids in HeLa cells (Fig EV4D–F) and quantified the effect on ERES with and without mechanical stimulation of cells. While KDELR2 or KDELR2‐ΔGAP overexpressing cells had similar number of ERES, cells overexpressing KDELR2‐GAP exhibited a significant ERES reduction under normal and mechanically challenged growth conditions (Fig 4E–H). During mechanical strain, inactive Rac1 at the ER (KDELR2‐GAP overexpression) reduced ERES count only in cells occupying large micropatterns (Fig 4G and H), but not in cells occupying small micropatterns (Fig EV4C and D). Overexpression of KDELR2‐GAP slightly reduced the total cellular pool of active Rac1 (Fig EV4H). The effect of KDELR2‐GAP on ERES is similar to those obtained with the knockdown or the pharmacological inhibition of Rac1, which affect every subcellular pool of Rac1. However, the KDELR2‐GAP only acts at the ER, and thus, these results point toward a functionally active Rac1 at the ER possibly regulating the ERES.

We next asked whether increasing Rac1 activation at the ER is sufficient to regulate ERES. To this end, we took advantage of CIBN‐CRY2‐based light‐inducible dimerization system (Kennedy *et al*, 2010) to recruit the Rac1‐specific GEF TIAM1 to the ER (Fig 4I). This approach has previously been used to generate spatially restricted active Rac1 at the leading edge in cells (de Beco *et al*, 2018). As presented in Fig 4I, cytosolic TIAM was successfully recruited to the ER by CIBN‐Sec61β, activating the ER pool of Rac1 in living HeLa cells. Following this, we counted ERES using SNAP‐tagged Sec16A as a marker, and noted an overall increase in ERES over time (Figs 4J and EV4I) further strengthening the notion of an involvement of active Rac1 in signaling to ERES.

### Effect of Rac1 on the early secretory pathway is actin independent

**Figure 5:**
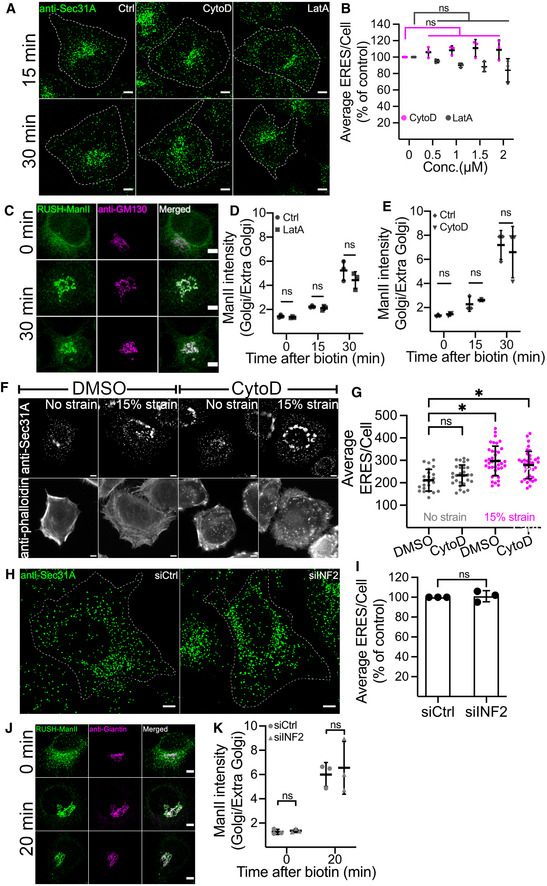
Effect of Actin disruption on ERES and ER‐to‐Golgi transport A, BRepresentative immunofluorescence images showing Sec31A labeled ERES in HeLa cells treated with DMSO (Ctrl), 0.5 μM cytochalasin D (CytoD) and 0.5 μM latrunculin A (LatA) for 15 and 30 min (A). Scale bar: 5 μm. The number of ERES per cell in HeLa cells treated with different doses of CytoD and LatA for 30 min are displayed as % of control ± standard deviation (90–100 cells from three experiments) (B).CDistribution of ManII‐RUSH in HeLa cells. Cells were pre‐treated with DMSO (Ctrl) or 1 μM LatA for 15 min prior to addition of biotin to initiate ManII‐trafficking. Golgi arrival for ManII‐RUSH was monitored for 30 min. Scale bar: 5 μm.D, EGraph showing ratio of ManII within Golgi to outside Golgi. Values were derived from HeLa cells treated with LatA or CytoD as described in (C). At least 30 cells per condition were used for quantification in each experiment (*n* = 3).FHeLa cells cultured on PDMS membranes were treated with DMSO or 1 μM cytochalasin D (CytoD) and ERES were labeled with anti‐Sec31A antibody before (no strain) or after 15% equibiaxial strain (15% strain). Antiphalloidin staining was performed to visualize actin in cells. Representative images are shown. Scale bars, 5 μm.GQuantification of experiment described in (F). The graph shows average ERES/Cell. Data were derived from a total of 90–115 cells from three experiments. Each dot represents several cells, and error bars show standard deviation.H, IsiRNA mediated depletion of INF2 does not phenocopy Rac1 effect. HeLa cells were transfected with nontargeting control siRNA (siCtrl) or siRNA against INF2 (siINF2) for 72 h before processing cells immunostaining with Sec31A to quantify ERES. Representative images showing Sec31A‐labeled ERES in HeLa cells (H), and quantification of ERES per cell presented as % of control (I). In total, 137 cells (siCtrl) and 113 cells (siINF2) were used for ERES quantification from three independent experiments. Scale bar in H is 5 μm.J, KKnockdown of INF2 does not alter the kinetics of ER‐to‐Golgi transport. HeLa cells stably expressing ManII‐RUSH were transfected with siRNAs as described in E, and the ER‐to‐Golgi transport of ManII‐RUSH was followed by confocal microscopy (J). Representative images for indicated time points are shown. Scale bars, 5 μm. The ratio of ManII‐RUSH within the Golgi to outside the Golgi was calculated for the indicated time points (K). Three independent experiments were performed and a total of 82 (siCtrl, 0 min), 72 (siINF2, 0 min) and 93 (siCtrl and siINF2, 20 min) were used for quantification. Data information: In all graphs, error bars represent standard deviation, *P‐value <0.05 and ns = nonsignificant. One‐way ANOVA was used to test for statistical significance for (B) and (G). Student's *t*‐test was used for the rest. Source data are available online for this figure.

**Figure EV5:**
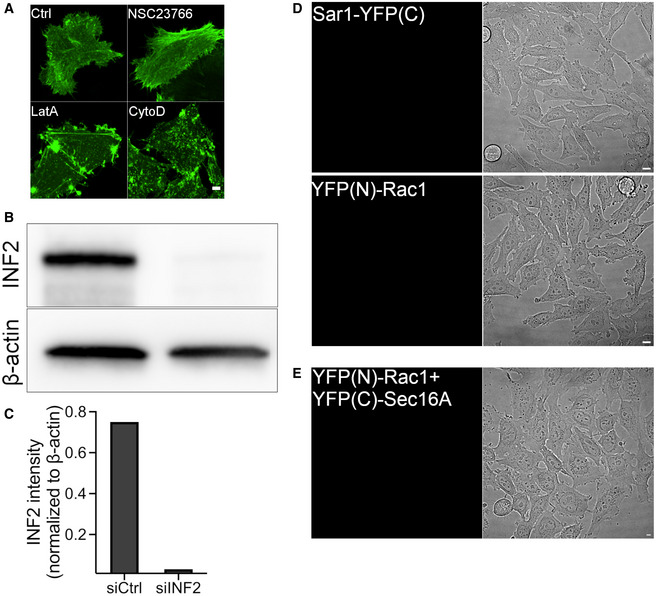
Controls for actin disruption and YFP‐BiFC ATreatment of HeLa cells expressing GFP‐tagged actin chromobody with 0.5 μM cytochalasin D (CytoD) or latrunculin A (LatA) for 15 min severely affects actin compared to DMSO (Ctrl) or Rac1 inhibitor (NSC23766, 50 μM for 4 h).B, CImmunoblot (B) and quantification graph (C) showing reduction in endogenous INF2 level in HeLa cells following transfection with 25 nM siRNA targeting INF2 (siINF2) for 72 h.DWhen expressed alone, Sar1‐YFP(C) and YFP(N)‐Rac1 show no YFP signal.EYFP(N)‐Rac1 and YFP(C)‐Sec16 cotransfected HeLa cells. Lack of positive YFP signal suggest no interaction of Rac1 and Sec16. Data information: Scale bar for immunofluorescence images (A, D, and E): 5 μm. Source data are available online for this figure.

We next aimed to unravel the underlying molecular details of Rac1 regulated ER‐export. Rac1 is pivotal in regulating actin dynamics, and actin assembly has previously been observed at the ER (Wales *et al*, 2016). Therefore, we asked whether actin mediates the observed effect of Rac1 on ERES and ER‐export. To test this, we treated HeLa cells under normal growth conditions with different concentrations of actin disrupting agents cytochalasin D (CytoD) and latrunculin A (LatA), and quantified ERES and ER‐to‐Golgi transport. Neither CytoD nor LatA treatment reduced ERES (Fig 5A and B) or affected the rate of the ER‐to‐Golgi transport (Fig 5C and E) as Rac1 did. In live cell imaging experiments, using the GFP‐tagged actin probe actin‐chromobody, we did verify that both drugs worked as anticipated Fig EV5A. Because actin cytoskeleton and its regulators are tightly coupled to mechanotransduction (Ohashi *et al*, 2017), we further explored whether actin mediates Rac1 signaling to ERES during mechanical strain. We cultured HeLa cells on PDMS membranes, treated them with DMSO (control) or CytoD, and exposed them to mechanical strain. We quantified ERES by immunolabeling endogenous Sec31A, a subunit of the COPII machinery. In agreement with the data presented in Figs 1A–E and EV1B, and C, mechanical strain increased ERES number (Fig 5F and G). However, actin disruption did not abrogate strain induced ERES increase (Fig 5F and G).

To perform a more targeted actin perturbation, we silenced the expression of inverted formin 2 (INF2) (Fig EV5B and C), which regulates actin dynamics locally at the ER (Wales *et al*, 2016). We determined ERES count (Fig 5H and I) or the kinetics of ER‐to‐Golgi transport (Fig 5J and K) in INF2 depleted cells and were unable to detect any appreciable changes compared with the control.

Based on these data, we conclude that the effect of Rac1 on the early secretory pathway is actin independent.

### Rac1 interacts with the COPII subunit Sar1 and stimulates vesicle budding

**Figure 6:**
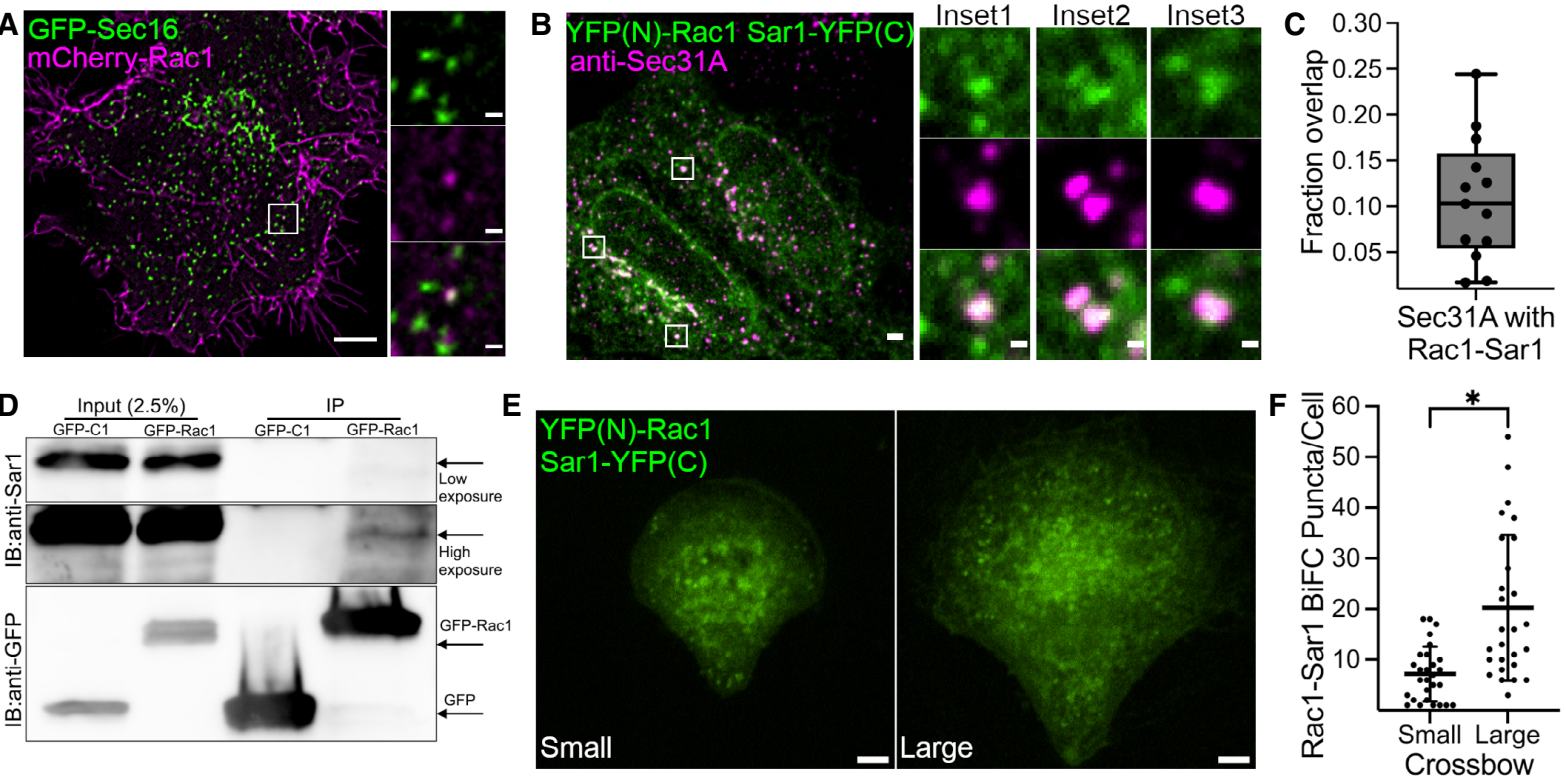
Rac1 regulates ERES and ER‐export by dimerizing with Sar1 AColocalization of mCherry‐Rac1 and GFP‐Sec16A in HeLa cells as monitored by SRRF live cell imaging. See also Movie EV4. Scale bars: 5 and 0.5 μm (inset)BRepresentative immunofluorescence images of HeLa cells cotransfected with YFP(N)‐Rac1 and Sar1‐YFP(C) and immunostained with Sec31A. Rac1 and Sar1 form complexes at ERES in HeLa cells, which partially overlap with Sec31A. Scale bars: 5 and 1 μm (insets).CBox plot showing the fraction of ERES that overlap with the Rac1‐Sar1 complex (number of cells = 24, number of experiments = 4). Individual data points shown in the graph represent number of images. Whiskers indicate min‐max range and central band indicates the median of all data.DGFP‐tagged Rac1 was immunoprecipitated by GFP‐trap and the fraction was immunoblotted for endogenous Sar1. Representative immunoblot showing coimmunoprecipitation of Sar1 by Rac1.ERepresentative immunofluorescence images of HeLa cells cotransfected with YFP(N)‐Rac1 and Sar1‐YFP(C) and cultured on crossbow micropatterns of indicated size. Cells were cotransfected with the indicated DNA constructs and incubated overnight prior to trypsinization and seeding on the micropatterned coverslip. Cells were allowed to grow for 3 h, fixed and processed for microscopy. Scale bar: 5 μm.FQuantification of Rac1‐Sar1 puncta described in (E) (number of experiments = 3, number of cells = 28 (small) and 27 (large)). In the quantification graph, error bars represent standard deviation and asterisk (*) denotes statistical significance at *P*‐value <0.05. A two‐tailed unpaired Student's *t* test was used for statistical testing. Source data are available online for this figure.

Because we observed Rac1 at ERES and functionally active Rac1 at the ER (Fig 4), we tested whether Rac1 directly signals to the constituents of the ER‐export machinery. We visualized mCherry‐tagged Rac1 together with GFP‐tagged Sec16A in HeLa cells, and observed a transient co‐occurrence of Rac1 with Sec16A (Fig 6A; Movie EV4) further suggesting a direct link between Rac1 and ERES. At ERES, recruitment of the small GTPase Sar1 is an early step during the process of COPII assembly. Therefore, we investigated whether Rac1 interacts with Sar1. Because the localization of Rac1 to ERES was very transient (Movie EV4), we opted for biomolecular fluorescence complementation (BiFC) to capture any transient Rac1‐Sar1 interactions. Overexpression of Sar1‐YFP(C) or YFP(N)‐Rac1 alone in HeLa cells gave no fluorescent signal (Fig EV5D). When cotransfected, YFP(C)‐Sar1 and YFP(N)‐Rac1 formed a fluorescent YFP complex (Fig 6B) indicating Rac1 and Sar1 as interaction partners. We observed that many of the Rac1‐Sar1 complexes formed puncta that colocalized with Sec31A, indicating Rac1‐Sar1 complexes form at ERES (Fig 6B and C). Moreover, we were also able to coimmunoprecipitate endogenous Sar1 with GFP‐tagged Rac1 in HeLa cells (Fig 6D) further supporting the BiFC results (Fig 6B). We also used BiFC to probe for Rac1 and Sec16A interaction and were unable to detect any complex formation (Fig EV5E). Finally, we tested whether the Rac1‐Sar1 complex formation was sensitive to a mechanical stimulus. Therefore, we expressed Sar1‐YFP(C) and YFP(N)‐Rac1 in HeLa that we cultured for 4 h on small and large micropatterns. We noticed that cells on large micropatterns had more Rac1‐Sar1 complexes (Fig 6E and F), which indicates that this interaction might be sensitive to mechanical strain. Together, these findings indicated that Rac1 may regulate ER export via its interaction with Sar1.

**Figure 7:**
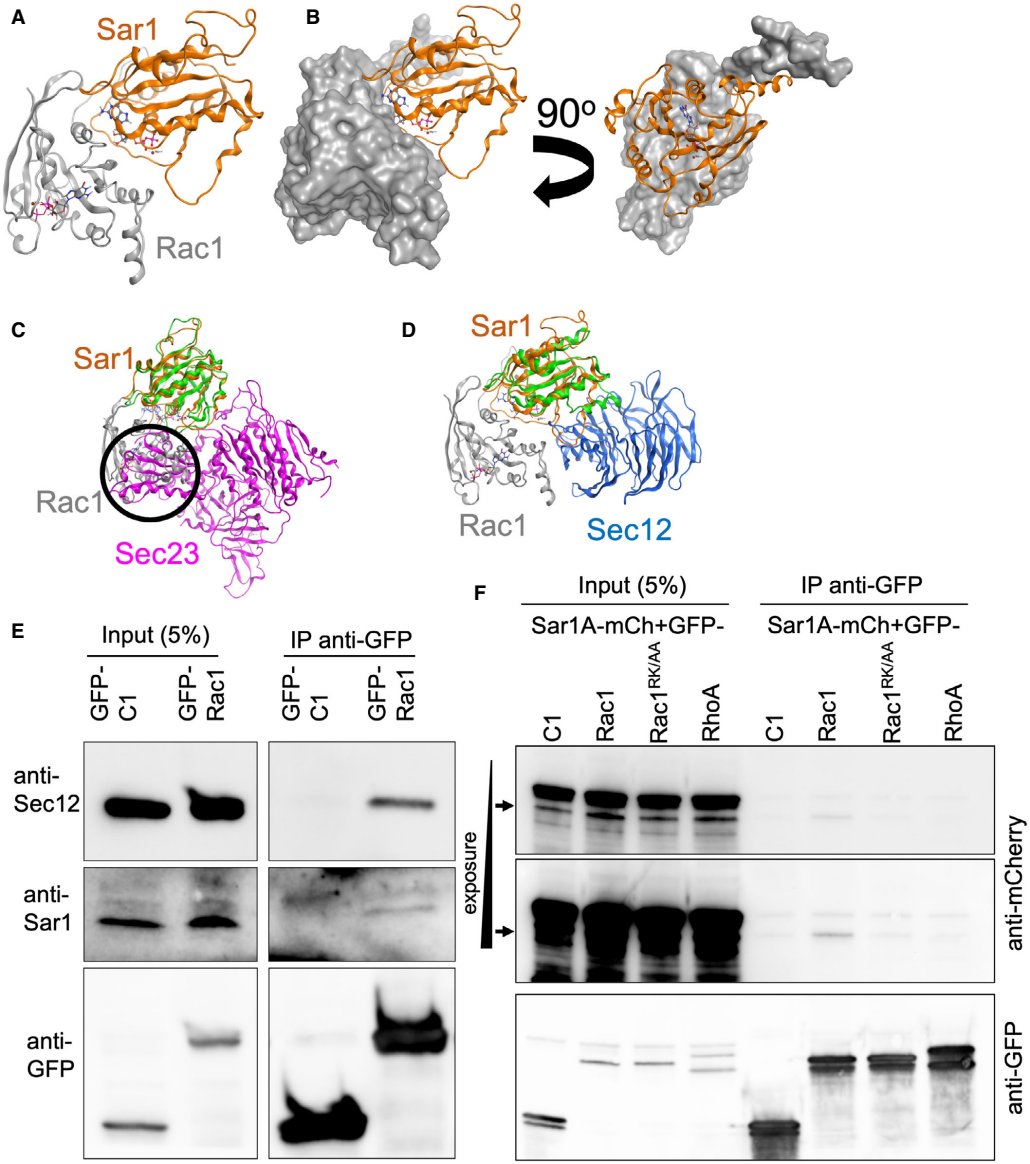
*In silico* modeling predicts a Rac1‐Sar1‐Sec12 ternary complex and its experimental validation A, B
*In silico* model showing the complex between Rac1 (gray) and Sar1 (gold) and how Rac1 covers the GTP binding site of Sar1. GTP of Rac1 in stick model, and of Sar1 in ball‐and‐stick.C
*In silico* model showing the steric clash between Rac1 and Sec23 (encircled), obtained by superposing the Sar1 units of the Sar1‐Sec23 (green‐purple) heterodimer onto the Sar1‐Rac1 heterodimer (gold‐gray).D
*In silico* model showing that the Sar1‐Rac1 heterodimer can interact with Sec12 by superposing the Sar1 units of Sar1‐Rac1 (gold‐gray) and Sar1‐Sec12 heterodimers (green‐blue).ECells expressing GFP‐Rac1 were lysed followed by immunoprecipitation against GFP and immunoblotting against endogenous Sar1 and Sec12. Blotting against GFP was performed to determine efficiency of the IP.FHeLa cells were cotransfected with the indicated plasmids and cells were incubated overnight prior to cell lysis and immunoprecipitation against GFP. Immunoblotting against anti‐mCherry was performed to detect Sar1A‐mCherry. The IP efficiency was determined using anti‐GFP. Different exposure times are shown for anti‐mCherry blot. Source data are available online for this figure.

We next explored the Rac1‐Sar1 interaction using *in silico* modeling, which suggested that Rac1 could cover the GTP binding site in Sar1 (Fig 7A and B), thereby preventing Sec23 (Sar1 GAP) from accessing Sar1, due to a steric clash between Sec23 and Rac1 (black circle in Fig 7C). On the contrary, our model predicts that the Sar1‐Rac1 dimer can interact with the Sar1 GEF Sec12 (Fig 7D). To validate this prediction, we performed co‐immunoprecipitation experiments. As shown in Fig 7E, immunoprecipitated GFP‐tagged Rac1 brought down endogenous Sar1 and Sec12, supporting the notion of the existence of a ternary complex and increasing the level of confidence in our *in silico* model. Based on the prediction from our *in silico* model, we created a Rac1 double mutant by substituting arginine at 163 (R163) and lysine at 166 (K166) with alanine (Rac1^RK/AA^), which we predict to reduce the ability to bind Sar1. We used co‐immunoprecipitation experiments to test this prediction. As an additional control, we included RhoA to determine whether the interaction is specific to Rac1. Indeed, Rac1^RK/AA^ exhibited a severely reduced ability to bind to Sar1 compared with wild‐type Rac1 (Fig 7F). We used RhoA as an additional control to test the specificity of Rac1 and Sar1 interaction and failed to detect Sar1 in the co‐immunoprecipitated fraction (Fig 7F). Taken together, these results further support the existence of a Rac1‐Sar1 complex and therefore strengthen the notion of Rac1‐dependent regulation of ERES.

**Figure 8:**
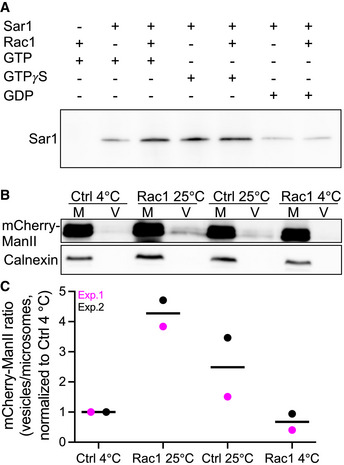
Rac1 stimulates the formation of active Sar1 and COPII AMicrosomes isolated from HeLa cells were incubated as indicated. Active Sar1 pulldown was then carried out, followed by immunoblotting against Sar1.BMicrosomes (M) from HeLa cells stably expressing mCherry‐ManII‐RUSH were incubated with ATP and an ATP‐regenerating system, GTP, biotin and cytosol in the presence or absence of recombinant Rac1 for 30 min at 25°C. COPII vesicles (V) were pelleted by ultracentrifugation (100,000 *g*) and immunoblotted for anti‐mCherry to detect ManII‐RUSH and anticalnexin (ER marker).CQuantification data of two independent vesicle budding assays. All values were normalized to 4°C Ctrl condition. Source data are available online for this figure.

Because Rac1 had a positive effect on ERES structure and function, we reasoned that Rac1 ought to exert a positive effect on Sar1. This can occur either because of enhanced Sar1 activation by Sec12, or reduced inactivation by Sec23. Our *in silico* model predicted that Sec23 is not capable of binding the Sar1‐Rac1 dimer. On the contrary, our combined experimental and computational data indicate that Sec12 can bind the complex. Therefore, we next investigated whether Rac1 affects the activation state of Sar1. To this end, we incubated microsomes with recombinant Sar1 followed by pulldown with an antibody directed to the active form of Sar1. Because microsomes contain Sec12, the GEF for Sar1, we observed Sar1 activation on microsomes (Fig 8A). When we included recombinant Rac1 in the assay, we obtained higher levels of active Sar1 (Fig 8A). No effect for Rac1 on Sar1 activation was observed when we performed the assay with GTPγS, which maximally activates Sar1 (Fig 8A). Likewise, when the assay was performed in the presence of GDP, only small amounts of active Sar1 were detected, and no effect of Rac1 on Sar1 activation was detectable (Fig 8A).

Active Sar1 promotes COPII assembly and thereby the formation of carriers from the ER. To determine whether recombinant Rac1 stimulates carrier formation, we used an *in vitro* COPII vesicle budding assay (Kim *et al*, 2005; Farhan *et al*, 2010). We used microsomes from HeLa cells stably expressing the RUSH cargo ManII. In the absence of biotin, ManII is only found in the ER. Microsomes were incubated with cytosol from HeLa cells, biotin, recombinant Rac1, GTP, and ATP regeneration system (see materials and methods for details). Vesicles and microsomes were separated by differential centrifugation. Performing the assay in the presence of Rac1 increased the amounts of mCherry‐ManII in the vesicle fraction compared with the fraction without exogenous Rac1 (Fig 8B and C). This data, together with the result from the active Sar1 pulldown (Fig 8A), suggest that Rac1 stimulates cargo exit from the ER through its interaction and positive modulation of the COPII subunit Sar1.

## Discussion

We and others established that ERES are platforms for the integration of several signaling pathways in response to mitogens (Farhan *et al*, 2010; Tillmann *et al*, 2015; Scharaw *et al*, 2016), or starvation (Zacharogianni *et al*, 2011), or the UPR and other ER‐localized signaling molecules (Centonze *et al*, 2019; Subramanian *et al*, 2019). Our current work expands this view by showing that ERES respond to mechanical stretching of cells. As a candidate mechanotransducer, we suggest the small GTPase Rac1 based on the following observations: (i) Rac1 is activated in cells occupying the large micropatterns or subjected to biaxial stretching; (ii) Rac1 inhibition or knockdown reduces the number of ERES; (iii) Rac1 localized to ERES and formed a complex with Sar1; (iv) Activation or inhibition of the ER pool of Rac1 regulates ERES number. Future work will need to fully elucidate the complete signaling cascade connecting mechanical strain and ERES, but our current work suggests Rac1 to be part of this cascade.

Mounting evidence suggests that small GTPases form dimers, consequently affecting their biological function. For instance,AUTHOR: dimerization of Arf1 (Diestelkoetter‐Bachert *et al*, 2020) and Sar1 (Hariri *et al*, 2014) was shown to regulate biogenesis of COPI vesicles and scission of COPII vesicles, respectively. Reportedly, Rac1 homodimerization regulates its intrinsic GTP hydrolyzing ability (Zhang *et al*, 2001). In contrast to homodimerization, only scant information is available on heterodimerization of small GTPases. Based on co‐immunoprecipitation and BiFC data, we propose that Rac1 and Sar1 form such a heterodimer. The endomembrane system hosts a wide range of small GTPases, and future studies are required to test whether Rac1 and/or Sar1 has other dimerization partners. While our results highlight a role for Rac1 in COPII‐dependent trafficking, it is likely that future work will uncover more endomembrane‐related functions of Rac1 and other small GTPases. All other downstream biological effects of Rac1 on ERES are compatible with the positive effect of Rac1 toward Sar1. This raises the question of how the Rac1‐Sar1 dimer could positively regulate ER‐export? The fact that we observe a ternary complex of Rac1, Sar1, and Sec12 suggests that Rac1 might promote Sar1 activation. This is supported by our finding of more Sar1‐GTP formed on microsomes in the presence of Rac1. In addition, our *in silico* data suggest that the Rac1‐Sar1 complex does not accommodate Sec23. A possible alternative interpretation of our data is that Rac1‐Sar1 heterodimerization prevents formation of Sar1 homodimers. Sar1 homodimerization was proposed previously to promote vesicle scission (Hariri *et al*, 2014), and therefore, the heterodimerization with Rac1 might delay vesicle scission, thereby extending the time for cargo loading and the formation of larger carriers. At this moment, we can only speculate about the existence of such carriers. In support of this speculation is our live imaging experiment where we observed that stretched cells exhibited more tubular ERES. However, we point out that the topic of whether ERES give rise to vesicles, tubules, or tunnels is a controversially discussed topic (Phuyal & Farhan, 2021), and further explorations along this line are certainly beyond the scope of the current work.

Another surprising finding was the identification of a very transient and small pool of endogenous Rac1 at the ER and at ERES. The high level of cytosolic Rac1 in any given cell type might have precluded the identification of this subcellular Rac1 pool with conventional confocal microscopy techniques. The ER pool of Rac1 is biologically active and is unlikely representing nascent biosynthetic Rac1. This is supported by the data showing that localized inactivation of Rac1 at the ER (with an ER‐targeted GAP) reduced the number of ERES. In addition, Rac1 formed complexes with Sar1 at ERES. The observation of Rac1 at the ER is in line with a previous finding (Woroniuk *et al*, 2018), which documented Rac1 signaling activity at the perinuclear region, a membrane system that is continuous with the ER. Reportedly, the C‐terminal fusion of CAAX motif of Rac1 to the green fluorescent protein GFP was sufficient for targeting GFP to the ER (Choy *et al*, 1999) further highlighting the intrinsic ability of Rac1 to associate with the ER membrane.

Our work establishes for the first time a mechanistic link between mechanotransduction and the regulation of ERES and demonstrates an important role of ERES for proper cellular adaptation in mechanically challenged cells. Previous research has established the regulation of ERES by intracellular stimuli (e.g., mitosis or unfolded proteins in the ER) or by extracellular chemical stimuli (e.g., nutrients and mitogens). Our work adds mechanical stimuli to the picture of the regulation of ERES. The increase in the number of ERES upon mechanical stimulation is unlikely a mere redistribution of ERES for the following reasons: First, if the increase was not a real one, but only due to redistribution of ERES, then it would be expected to occur in any condition, irrespective of the biological context. However, we observe the increase only in control cells and not in Rac1 inhibited or depleted cells. Second, the increase of ERES is in line with the acceleration of ER‐to‐Golgi trafficking as observed in the RUSH assay. A mere redistribution will not cause a change in ER‐to‐Golgi trafficking. This will pave the way for future investigations on the role of other mechanical stimuli such as stiffness, compression, or shear stress. The mechanisms by which Rac1 activity is regulated during mechanotransduction are poorly understood, although studies have proposed a role for mechanosensitive channels and focal adhesion molecules at the cell surface (Bae *et al*, 2014; Arya *et al*, 2020). Future work is needed to characterize the receptors or sensors at the cell surface that signal to Rac1.

Finally, our work has implications for the regulation of proteostasis in response to changes in cell size. We noticed that the absolute number of ERES is higher in cells forced to grow on larger micropatterns. The same was true when we expanded the cell area using acute stretching. This shows that cells are capable of adapting their secretory capacity when they expand in size. In fact, a recent preprint showed that different organelles undergo changes dependent on the cell size (Lanz *et al*, 2021). Future work could focus on studying how cell size affects proteostasis by controlling the rate of protein synthesis, secretion, and degradation.

## Materials and Methods

### Cell culture and treatment

HeLa cells were maintained in DMEM (Lonza, #12‐604F) supplemented with 10% FCS (Life Technologies, #10500064), 50 units/ml penicillin and 50 μg/ml streptomycin (Lonza, #DE17‐603E), in a humidified 5% CO_2_ atmosphere at 37°C. PC3 cells and MDA‐MB‐231 cells were cultured in RPMI (Sigma, #R8758) medium. RPE‐1 cells were maintained in DMEM/F‐12 (Life Technologies, #31331028) medium. All cell culture media contained the same supplements as for HeLa cells. CRISPR Rac1 knockout PC3 cells were extensively characterized previously (Baker *et al*, 2020).

For all Rac1 inhibition experiments, cells were treated with 50 μM NSC23766 or 0.1% (v/v) DMSO in complete medium for 4 h. For actin disruption experiments, cells were treated with 0.5, 1, 1.5, and 2 μM cytochalasin D or latrunculin A in complete medium for 15 and 30 min. For RUSH assays, cells were preincubated with 1 μM cytochalasin D or latrunculin A for 15 min before starting the cargo release with biotin.

siRNA oligos were delivered to the cells using HiPerFect (Qiagen, # 301707) transfection reagent. Briefly, 10–20 nM siRNA duplexes were complexed with the transfection reagent in 100 μl serum and antibiotics free cell culture medium, incubated at room temperature inside the laminar flow hood for 5 min and added to the cells immediately after seeding for experiments. Knockdown efficiency after 48 or 72 h was measured to check that the levels of desired proteins were reduced. For Rac1 knockdown experiments, the siGenome nontargeting siRNA pool from Dharmacon were used as control. The AllStars negative control siRNA from Qiagen was used as control for INF2 knockdown experiments (Table 1).

**Table 1:** List of reagents and plasmids.

Name	Vendor	Cat. no.	Sequence	Remarks
NSC23766	Tocris	2161		
Cytochalasin D	Sigma	C8273		
Latrunculin A	Tocris	3973		
GTP	Sigma	G8877		
Creatine phosphate	Sigma	10621714001		
Creatine Kinase	Sigma	10127566001		
Magnesium acetate tetrahydrate	Sigma	M5661‐50G		
Potassium acetate	Sigma	236497‐100G		
Adenosine 5′‐triphosphate disodium salt hydrate	Sigma	A26209‐1G		
Cytoo Starters Kit	CYTOO	10–900–00‐18		
Anti‐Rac1 Antibody, clone 23A8	Merck	05‐389		
anti‐Giantin	Covance	PRB‐114P		
anti‐GM130	BD Biosciences	610823		
anti‐INF2	ProteinTech group	20466‐1‐AP		
anti‐Sar1	abcam	ab125871		
Anti‐Sec31A Clone 32/Sec31A	BD Biosciences	612351		
anti‐GFP	Roche	13026100		
anti‐Calnexin (E‐10)	SantaCruz	sc‐46669		
HRP mouse	Jackson Immuno‐research	115035003		
HRP rabbit	Jackson Immuno‐research	111035144		
Alexa Fluor 488	Thermo Fisher Scientific	A‐11001		Goat anti‐Mouse IgG (H + L) Cross‐Adsorbed Secondary Antibody
Alexa Fluor 568	Thermo Fisher Scientific	A‐11031		Goat anti‐Mouse IgG (H + L) Highly Cross‐Adsorbed Secondary Antibody
Alexa fluor 647	Thermo Fisher Scientific	A‐21235		Goat anti‐Mouse IgG (H + L) Cross‐Adsorbed Secondary Antibody
Alexa Fluor 488	Thermo Fisher Scientific	A‐11008		Goat anti‐Rabbit IgG (H + L) Cross‐Adsorbed Secondary Antibody
Alexa Fluor 568	Thermo Fisher Scientific	A‐11011		Goat anti‐Rabbit IgG (H + L) Highly Cross‐Adsorbed Secondary Antibody
Alexa fluor 647	Thermo Fisher Scientific	A‐21244		Goat anti‐Rabbit IgG (H + L) Cross‐Adsorbed Secondary Antibody
Sar1 activation assay kit	NewEastBio	NB‐81801		
Recombinant Rac1	NewEastBio	NB‐10101		
Recombinant Sar1	NewEastBio	NB‐10114		
Fugene 6	Promega	E2692		
HiPerfect	Qiagen	301707		
Rac1 siRNA #1	Dharmacon	M‐003560‐06‐0005	5′‐UAAGGAGAUUGGUGCUGUA‐3′ 5′‐UAAAGACACGAUCGAGAAA‐3′ 5′‐CGGCACCACUGUCCCAACA‐3′ 5′‐AUGAAAGUGUCACGGGUAA‐3′	
Rac1 siRNA #2	ThermoFisher Scientific	AM51331 (siRNA ID: 120600)	5′‐CCUUUGUACGCUUUGCUCAtt‐3′	used for rescue experiments
siINF2	Qiagen	SI00319165	5′‐CCGCTTCAGCATTGTCATGAA‐3′	
AllStars Negative Control siRNA (5 nmol)	Qiagen	1027280		
Silencer™ Select Negative Control No. 1 siRNA	ThermoFisher Scientific	4390843		
siGenome Non‐Targeting Control siRNA Pool #1	Horizon Discovery (Dharmacon)	D‐001206‐13‐05		
GFP‐Sec16A	Addgene	15776		
SNAP‐Sec16A	This paper		FWD:5′‐atatagatctcgaggaatgcctgg‐3′ REV:5′‐atatgaattcCTAGTTCAGCACCAG‐3′	Cloned using EcoRI and BglII
GFP‐Rac1	Addgene	13719		
mCherry‐Rac1	Substituted GFP in GFP‐Rac1 with mCherry from pmCherry‐N1 using HindIII and BsrgI			
YFP(N)‐Rac1	This paper		FWD: 5′‐AATTCTCGAGCAATGCAGGCCATCAAGTGT‐3′ REV: 5′‐TTAAGGGCCCTTACAACAGCAGGCATTTTC‐3′	Cloned using XhoI and ApaI
Sar1‐YFP(C)	This paper		FWD:5′‐tatagaattcATGTCTTTCATCTTTGA‐3′ REV: 5′‐tataatcgatGTCAATATACTGGGA‐3′	Cloned using ClaI and EcoRI
KDELR2‐GFP	This paper		FWD:5′‐tatactcgagATGAACATTTTCCGGCTG‐3′ REV: 5′‐atataagcttTGCTGGCAAACTGAGCTT‐3′	Cloned using XhoI and HindIII into pmEGFP‐N1
KDLER2‐GAP‐GFP	This paper		FWD:5′‐tatagaattctaTGTGACCTCACAACAC, REV:5′‐tataggatccttGAATAAAACGTCTTCG‐3′	Cloned using EcoRI and BamHI into KDELR2‐GFP
KDELR2‐dGAP‐GFP	This paper	dGAP was synthesized as gBlock and cloned into KDELR2‐GFP using the same primers as for KDELR2‐GAP‐GFP		
GFP‐Sec61β	Gift from Lei Lu, Nanyang Technological University, Singapore			
pTriEx4‐Rac1‐2G	Addgene	66110		
CIBN‐GFP‐Sec61β	Addgene	104177		
CRY2‐TIAM‐mCherry	Gift from Mathieu Coppey			

All plasmid transfections were carried out using Fugene 6 (Promega, #E2691) (used at 3 μl per μg DNA) transfection reagent following the manufacturer's protocol. Cells were transfected with 750 ng of plasmid DNA. Unless otherwise stated, all listed concentrations of siRNAs and chemicals are final concentrations.

### Immunofluorescence staining

Cells were fixed with 4% paraformaldehyde for 12 min, washed three times with PBS, quenched for 5 min with 50 mM NH_4_Cl, and washed again twice with PBS. After permeabilization with 0.2% (v/v) triton X‐100 for 4 min, cells were washed twice with PBS, and blocked for 30 min in 1% (wt/vol) BSA. Finally, cells were incubated for 1 h with the primary antibody, washed thrice with PBS, incubated for 1 h with secondary antibody, and mounted with antifading Polyvinyl alcohol mounting medium with DABCO® (Sigma, #10981) after washing three times with PBS. All incubation steps were carried out at room temperature, triton X‐100 and BSA dilutions were prepared in PBS, and each PBS washing steps were 5 min on a shaker.

### FRAP microscopy

HeLa cells were seeded on 35 mm MatTek dishes (MatTek corporation, #P35G‐1.5‐20‐C) and incubated overnight. Cells were then transfected with 0.75 μg of GFP‐Sec16A plasmid and incubated overnight. Prior to FRAP experiments, cells were treated with DMSO or with Rac1 inhibitor NSC23766 for 4 h. The experiment was performed on a Zeiss LSM700 confocal microscope equipped with a 63× oil‐immersion objective (Plan‐Apochromat 63×/1.40 NA Ph3 M27) at 37°C. Images were acquired using Zeiss Zen software. A prebleach image was acquired before bleaching individual ERES at 100% laser intensity (10 iterations) and subsequent image acquisition at one image per second.

FRAP analysis was carried out as described previously (Farhan *et al*, 2010; Centonze *et al*, 2019).

### Preparation of polydimethylsiloxane (PDMS) membranes

Stretchable PDMS (Sylgard Silicone Elastomer Kit, Dow Corning) membranes were prepared as previously described (Kosmalska *et al*, 2015). Briefly, a mix of 10:1 base to crosslinker ratio was spun on plastic wafers for 1 min at 500 rpm and then cured overnight at 65°C. Once polymerized, membranes were peeled off the wafers and assembled onto a metal ring for fibronectin coating, cell seeding, and microscopy experiments.

### Mechanical stimulation of the cells and live cell imaging or ERES staining

Cell mechanical stimulation was performed as previously described (Kosmalska *et al*, 2015). Briefly, a 150 μl droplet of a 10 μg/ml fibronectin solution (Sigma, #F0895) was deposited in the center of the membrane mounted in the ring. After overnight incubation at 4°C, the fibronectin solution was rinsed. HeLa cells transiently transfected with GFP‐Sec16A were then seeded on the fibronectin‐coated membranes and allowed to attach for up to 1 h. Then, ring‐containing membranes were mounted in the stretch system as previously described (Kosmalska *et al*, 2015). Live‐cells were subjected to a 7.5% equibiaxial strain. Images of the cells were acquired with 500 ms interval frames, with a 60x objective (NIR Apo 60X/WD 2.8, Nikon) mounted in an inverted microscope (Nikon Eclipse Ti) with a spinning disk confocal unit (CSU‐W1, Yokogawa), a Zyla sCMOS camera (Andor) and using the Micromanager software.

For actin disruption experiments, Hela cells were seeded on the fibronectin‐coated PDMS membranes mounted on the stretch system and allowed to attach for up to 1 h. 15 min before stretch, samples were incubated with either 1 μM cytochalasin D in DMSO or DMSO only. Living cells were then subjected to a 15% stretch for 3 min at 37°C, and cells were subsequently fixed at room temperature with 4% PFA. After 12‐min incubation with the fixative, cells were washed with PBS and stretch was slowly released. Samples without stretch were prepared by sticking a PDMS sheet in a MaTtek dish and preparing the fibronectin coating, cells seeding and drug incubation as for the stretch condition. For immunostaining of ERES in these samples, staining conditions were as follow: 10‐min quenching with 50 mM NH_4_Cl, 4‐min permeabilization with 0.2% triton X‐100. After wash, samples were blocked for 1 h with 2% fish gelatin (Sigma Aldrich, #G7765). All the subsequent steps were performed in 2% fish gelatin. Cells were incubated with the primary anti‐Sec31 antibody for 1 h. After wash (3 times 5 min), samples were incubated with both the secondary goat anti‐mouse antibody‐alexa488 (Thermofisher A11029) at 1:500 dilution and Phalloidin‐TRITC (555) (Sigma‐Aldrich P1951, 0.1 mg/ml) for 1 h. All buffer dilutions were prepeared in PBS at given concentrations. Samples were washed, and buffer was exchanged to PBS. Images were acquired with a 60× objective (NIR Apo 60X/WD 2.8, Nikon) mounted in an upright epifluorescence microscope (NIR Apo 60X/WD 2.8, Nikon) and an Orca Flash 4.0 camera (Hamamatsu), using the Metamorph software. For the stretch conditions, samples were slowly restretched to a 15% equibiaxial stretch before imaging.

For Rac1‐FRET assay and the RUSH assay, HeLa cells were subjected to a 7.5% equibiaxial strain. Images were acquired with a 60× objective (NIR Apo 60X/WD 2.8, Nikon) mounted in an upright epifluorescence microscope (NIR Apo 60X/WD 2.8, Nikon) and an Orca Flash 4.0 camera (Hamamatsu), using the Metamorph software.

### ERES and CellMask staining of cells cultured on micropatterned surfaces

We purchased fibronectin‐coated micropatterned chips containing multiple geometries and sizes from CYTOO SA. After seeding, cells were allowed to spread on the micropatterned surfaces for up to 4 h. At the end of incubation, cells were fixed and processed for immunostaining. For quantification of ERES, anti‐Sec31A staining was carried out as described above. For counting cells failing to cover the given geometry, cells were fixed and stained with CellMask™ Deep Red Plasma membrane Stain (ThermoFisher Scientific, #C10046) for 5 min, washed extensively for 3 × 5 min with PBS and mounted for imaging.

### Live cell imaging and optogenetics

Live cell imaging of HeLa cells co‐expressing mCherry‐Rac1 and GFP‐Sec61 or mCherry‐Rac1 and GFP‐Sec16A was performed on Andor Dragonfly spinning disk equipped with a Nikon Ti2E inverted optical microscope (60× TIRF objective (Plan‐APOCHROMAT 60×/1.49 Oil)) and an EMCCD camera (iXon Ultra 888, Andor). Cells were maintained at 37°C in 5% CO_2_ in a heating chamber (Oko lab). SRRF‐Stream mode in Fusion (version 2.1, Andor) was used for image acquisition with an additional 1.5× magnification. Following imaging parameters were used. SRRF Frame count: 150–200, SRRF Radiality Magnification: 4×, SRRF Ring Radius: 1.4 px, SRRF Temporal Analsysis: Mean and SRRF FPN correction: 75–100 frames.

For optogenetics, HeLa cells expressing CIBN‐GFP‐Sec61β, Cry2‐TIAM‐mCherry and SNAP‐Sec16A were stimulated for 30 s with a 488 nm laser.

### Retention using selective hooks (RUSH) assay

A step‐by‐step protocol for RUSH assay has been described elsewhere (Boncompain & Perez, 2012). We used HeLa cells stably expressing the Golgi protein Mannosidase II in RUSH system, and monitored its arrival at the Golgi for up to 30 min in our experiments.

### ERES, RUSH quantification, and colocalization analysis

ERES, RUSH quantification, and colocalization analyses were all carried out using Fiji (Schindelin *et al*, 2012). Prior to analysis, background was subtracted from the immunofluorescence images using “subtract background” command in Fiji, where a rolling ball radius of 50 pixels was used. To obtain ERES count in cells, images were thresholded, and ERES were counted using “Analyze particle” command. Counts were then normalized to total cells. All images within the same experiment were processed in the exact same way for quantification.

In live cell imaging equibiaxial strain experiments, the number of GFP‐Sec16A marked ERES in each frame was detected and counted using “Find maxima” in Fiji after background subtraction as described above.

For quantification of RUSH experiments, ManII intensity in the Golgi was measured by drawing a ROI. To measure extra Golgi intensity of ManII, the ROI was then transferred outside the Golgi. Ratio of ManII at the Golgi to extra Golgi region was calculated for data visualization.

The fraction overlap of Rac1‐Sar1 complex with Sec31 was calculated using DiANA (Gilles *et al*, 2017) plugin in Fiji.

### Cell lysis, immunoprecipitation, and immunoblotting

Cells were washed twice with ice‐cold PBS, scraped off in lysis buffer (50 mM Tris–HCl, 300 mM NaCl, 1 mM EDTA, 0.5% Triton X‐100, pH 7.4) supplemented with EDTA‐free proteinase and phosphatase inhibitor mixture and incubated in ice for 10 min. Cell lysates were then centrifuged at 20,000 *g* for 10 min at 4°C. The supernatant was collected and solubilized in loading buffer. Samples were run on 4–20% TGX gels (BioRad), and proteins were transferred to a nitrocellulose membrane using semidry transfer system. Once the transfer was complete, membranes were blocked (in 5% (wt/vol) milk in TBS with 0.1% Tween) and incubated first with the specified primary antibodies, washed and then with the HRP‐conjugated secondary antibodies. Finally, blots were visualized using ECL clarity chemiluminescence reagent (BioRad, #1705061) on ChemiDoc (BioRad).

For immunoprecipitation experiments, cells were lysed in immunoprecipitation buffer (20 mM Tris–HCl, pH 7.4, 150 mM NaCl, 1 mM MgCl2, 10% glycerol, 0.5% NP‐40) and GFP‐trap (ChromoTek, #gta‐100) was performed following the manufacturer's protocol.

### Rac1 activity assay

For measuring the level of activation of Rac1, luminescence‐based Rac1 G‐LISA Activation Assay (Cytoskeleton Inc. #BK126) kit was used. The assay was carried out following the protocol (freely available at manufacturer's website) provided with the kit without any modifications. Cell lysates prepared from HeLa cells stably overexpressing ER‐targeted Rac1‐GAP, KDEL R2, or ER‐targeted catalytically inactive Rac1‐GAP were used for the measurements.

### Active Sar1 pulldown assay

Sar1 activation assay kit (NewEast Biosciences, #81801) and the protocol provided with the kit was followed for active Sar1 pulldown from microsomes.

To isolate microsomes, HeLa cells were scraped in ice‐cold PBS and pelleted by centrifugation at 500 *g* for 10 min. The pellet was resuspended in 400 μl buffer containing 10 mM Hepes‐KOH, pH 7.2, 250 mM sorbitol, 10 mM KOAc, and 1.5 mM MgOAc. Cells were homogenized by passing through a 25‐gauge syringe for at least 30 times and centrifuged at 1,000 *g* for 10 min. The postnuclear supernatant was then centrifuged at 6,000 *g* for 10 min. The pellet was resuspended in ice‐cold PBS and centrifuged again at 6,000 *g* for 10 min. Finally, the pellet was resuspended in 100 μl of 1× assay/lysis buffer provided with the kit and used for active Sar1 pulldown.

To the microsomal fractions, 750 ng of recombinant Rac1 or Sar1 or both, and GTP or GTPγS (100 μM final concentration) or GDP (1 mM final concentration) were added. The reaction mixture was then incubated at 30°C with constant agitation for 30 min for GTP‐loading prior to active Sar1 pulldown with anti‐active Sar1 monoclonal antibody provided in the kit.

### Vesicle budding assay

We prepared microsomes and cytosol for vesicle budding assay following the previously published protocol from the laboratory (Farhan *et al*, 2010). Microsomes were isolated from HeLa cells stably expressing ManII‐RUSH, whereas cytosol was prepared from HeLa cells.

For budding reactions, 30 μl of microsomes, 65 μl of cytosol, 40 μM biotin, 0.5 mM GTP, and an ATP regeneration system (1 mM ATP, 25 mM creatine phosphate, and 0.3 mg/ml creatine kinase) was used. Vesicle budding reactions were carried out either at 25°C or on ice (control) for 30 min. In the Rac1‐ and Sar1‐containing reactions, 500 ng recombinant proteins were added. The reaction was stopped by placing the tubes on ice. Finally, the reaction mixture was centrifuged at 100,000 *g* for 30 min to pellet the budded vesicles.

### Statistical analyses

The number of experiments and the number of cells used for generating data are indicated in the respective figure legends. All statistical analysis was performed in GraphPad Prism 9 (Version 9.3.1). Where appropriate, we performed a Student's unpaired *t* test to evaluate statistical significance of the data. For comparing multiple means, we used one‐way analysis of variance (ANOVA) followed by the Dunnett's multiple comparison's test. In Fig 3D, Tukey's multiple comparison test was applied following one‐way ANOVA. In the figures, data are presented as mean ± standard deviation from at least three independent experiments, unless otherwise indicated in the figure legends. In the figures, asteriks (*) denote statistical significance at *P*‐value <0.05.

### Rac1 GFP tagging

Rac1 was gene edited using a modified PITCh technology (Sakuma *et al*, 2016) adjusted for generating N‐terminal fusion proteins (Lin *et al*, 2019). A Rac1‐specific gRNA 5′ ACACTTGATGGCCTGCATCA was cloned into plasmid pX330‐BbsI‐PITCh (Addgene plasmid #127875) and transfected along with pN‐PITCh‐GFP‐Rac1 into HeLa cells using JetPrime (Polyplus, Illkirch, France). Plasmid pN‐PITCh‐GFP‐Rac1 contains a Puromycin resistance‐T2A‐eGFP cassette flanked by 50 bp of genomic RAC1 sequence flanking the CRSIPR‐CAS9 induced DNA double‐strand break. This plasmid was constructed by HiFi‐ mediated *in vitro* recombination (NEB) of two PCR‐based fragments generated by amplifying the pN‐PITCh‐GFP (Addgene plasmid #127888) vector backbone using primers 5′ caaacacgtacgcgtacgatgctctagaatg and 5′ tgctatgtaacgcggaactccatatatggg and the Rac1 sequence flanked Puro‐GFP cassette from pN‐PITCh‐GFP using primers 5′ ccgcgttacatagcatcgtacgcgtacgtgtttggGGCCCAGCGAGCGGCCCTGAtgaccgagtacaagcccacg and 5′ cattctagagcatcgtacgcgtacgtgtttgggACCACACACTTGATGGCCTGCAtcttgtacagctcgtccatgccgag. Correct recombinants were verified by DNA sequencing. After transfection, cells were selected using 2.5 μg/ml puromycin and clones established by limiting dilution.

### Modeling

Homology models of human Sar1 and Rac1 were generated using the YASARA code (Krieger & Vriend, 2014) and the default homology modeling macro. In short, PSI‐BLAST searches are made to get position‐specific scoring matrices from UniRef90, followed by searching the PDB for matching templates. Based on BLAST e‐value and alignment scores, up to five templates are selected and up to five models generated with each template. The different models are ranked based on 1D and 3D packing. Hybrid models can then be generated by, for example, replacing loop segments and similar from the chosen main model by same parts from another model, that locally has obtained a better score.

The homology model of Sar1 is based on the Xray structure of the Sar1 dimer from *Cricetulus griseus* (PDB‐ID 1F6B), containing GDP bound to the pocket. For Rac1, the main template used for the final GTP bound model is the NPH1 protein of *Avena sativa* (PDB‐ID 2WKP) with N‐ and C‐terminal segments taken from the crystal structure of human Rac1 (PDB‐ID 1MH1).

The homology models of Sar1 and Rac1 were then subjected to protein–protein docking using a modified protocol of our recently developed meta‐approach (Mahdizadeh *et al*, 2021). The on‐line servers for the protein–protein docking engines ClusPro (Comeau *et al*, 2004; Kozakov *et al*, 2017), FireDock (Andrusier *et al*, 2007; Mashiach *et al*, 2008), Galaxy‐TONGDOCK (Ko *et al*, 2012), PatchDock (Schneidman‐Duhovny *et al*, 2005), and ZDOCK (Chen *et al*, 2003; Pierce *et al*, 2011) were employed using blind docking and default settings, and the 10 best scoring models from each downloaded. The 50 models were clustered based on RMSD values and evaluation of Kelley penalties (Kelley *et al*, 1996) to obtain the optimum number of clusters, using the Clustering of complexes module in the Schrödinger software [Schrödinger Release 2020–2: Maestro, Schrödinger, LLC, New York, NY, 2020.]. As consensus model was then selected the predicted complex with lowest RMSD from the centroid of the most populated cluster which in this case contained 14 of the total predicted 50 complexes.

The obtained Rac1‐Sar1 complex was analyzed, and superposed onto the Sar1‐Sec12 and Sar1‐Sec23 crystal structures from *Saccharomyces cerevisae*, with PDB‐IDs 6X90 and 1M2O, respectively. Superposition was made such that the two Sar1 units would be optimally aligned. All superposition and images were generated using the Molecular Operating Environment software [Molecular Operating Environment (MOE) 2019.01; Chemical Computing Group, Montréal, Canada, 2019].

## Author contributions

**Santosh Phuyal:** Conceptualization; data curation; formal analysis; supervision; validation; investigation; visualization; methodology; writing – original draft. **Elena Djaerff:** Formal analysis; methodology. **Anabel‐Lise Le Roux:** Conceptualization; data curation; formal analysis; investigation; methodology; writing – review and editing. **Martin J Baker:** Methodology. **Daniela Fankhauser:** Methodology. **Sayyed Jalil Mahdizadeh:** Formal analysis; investigation; methodology; writing – review and editing. **Veronika Reiterer:** Methodology; project administration; writing – review and editing. **Amirabbas Parizadeh:** Methodology. **Edward Felder:** Supervision; methodology. **Jennifer C Kahlhofer:** Formal analysis; methodology. **David Teis:** Supervision; methodology; writing – review and editing. **Marcelo G Kazanietz:** Supervision; methodology. **Stephan Geley:** Supervision; methodology; project administration. **Leif A Eriksson:** Data curation; formal analysis; supervision; investigation; methodology; writing – review and editing. **Pere Roca‐Cusachs:** Formal analysis; funding acquisition; investigation; methodology; writing – original draft; writing – review and editing. **Hesso Farhan:** Conceptualization; data curation; formal analysis; supervision; funding acquisition; investigation; methodology; writing – original draft; project administration; writing – review and editing.

## Disclosure and competing interests statement

The authors declared that they have no conflict of interest. PRC is an EMBO member; this has no bearing on the editorial consideration of this article for publication.

## Supporting information




Expanded View Figures PDF

Movie EV1

Movie EV2

Movie EV3

Movie EV4

Source Data for Expanded View

PDF+

Source Data for Figure 5

Source Data for Figure 6

Source Data for Figure 7

Source Data for Figure 8

## Data Availability

This study includes no data deposited in external repositories.
