# Supplementary material for: Mechanical strain stimulates COPII‐dependent secretory trafficking via Rac1
Source: EMBO J. 2022 Aug 8;41(18):e110596. doi: 10.15252/embj.2022110596 (PMC9475550; doi:10.15252/embj.2022110596)
Supplement: Supplementary file 1 — Expanded View Figures PDF [file EMBJ-41-e110596-s010.pdf]

# Expanded View Figures

**Figure EV1. ERES are regulated by an increase in cell area.**

A Quantification graph showing area covered by HeLa cells on Crossbow-, Disc-, H- or Yshaped fibronectin-coated micropatterned chips. 50–60 cells from two independent experiments were counted. Error bars show standard deviation, and dots represent individual cells.

B, C Representative images showing Sec31A labeled ERES in HeLa (B) and RPE-1 cells (C) seeded on fibronectin-coated micropatterned chips of indicated geometries and sizes. Cells were treated with either DMSO (Ctrl) or Rac1 inhibitor (NSC23766) for 4 h prior to immunolabeling with Sec31A. Scale bars: 5 µm.

D, E Quantification data for (C). Dot plot shows ERES in individual RPE-1 cells cultured on large (D) and small (E) micropatterned surfaces. At least 42 cells were used for ERES quantification. $n = 3$ (biological replicates), *$P$-value <0.05, Student's $t$-test. Error bars show standard deviation. ns = nonsignificant.

F, G HeLa cells were subjected to osmotic shock for the indicated time point and changes in ERES was monitored by counting Sec31A marked ERES. (F) Representative immunofluorescence images showing ERES in control cells and cells subjected to osmotic shock. Scale bar: 5 µm (G) Quantification of ERES in (C). At least 30 cells were counted in each experiment ($n = 3$). Mean ± standard deviation and dots representing average ERES/cell values from each experiment are shown. *$P$-value <0.05, Student's $t$-test.

H, I Graphs show area covered by HeLa cells treated with DMSO (Ctrl) or Rac1 inhibitor (NSC23766) grown on small (E) or large (F) fibronectin-coated micropatterned chips. Between 50 and 60 cells were counted in two experiments. Values show average cell area ± standard deviation.

J Quantification graph showing increase in size of HeLa cells during 7.5% biaxial strain. A total of 19 cells were quantified in four live cell imaging experiments. Cell size before strain was used to calculate percent increase in cell size during strain. Mean ± standard deviation and dots representing average values from each experiment are shown. A two-tailed Student's unpaired $t$-test was used to determine the statistical significance.

© 2022 The Authors

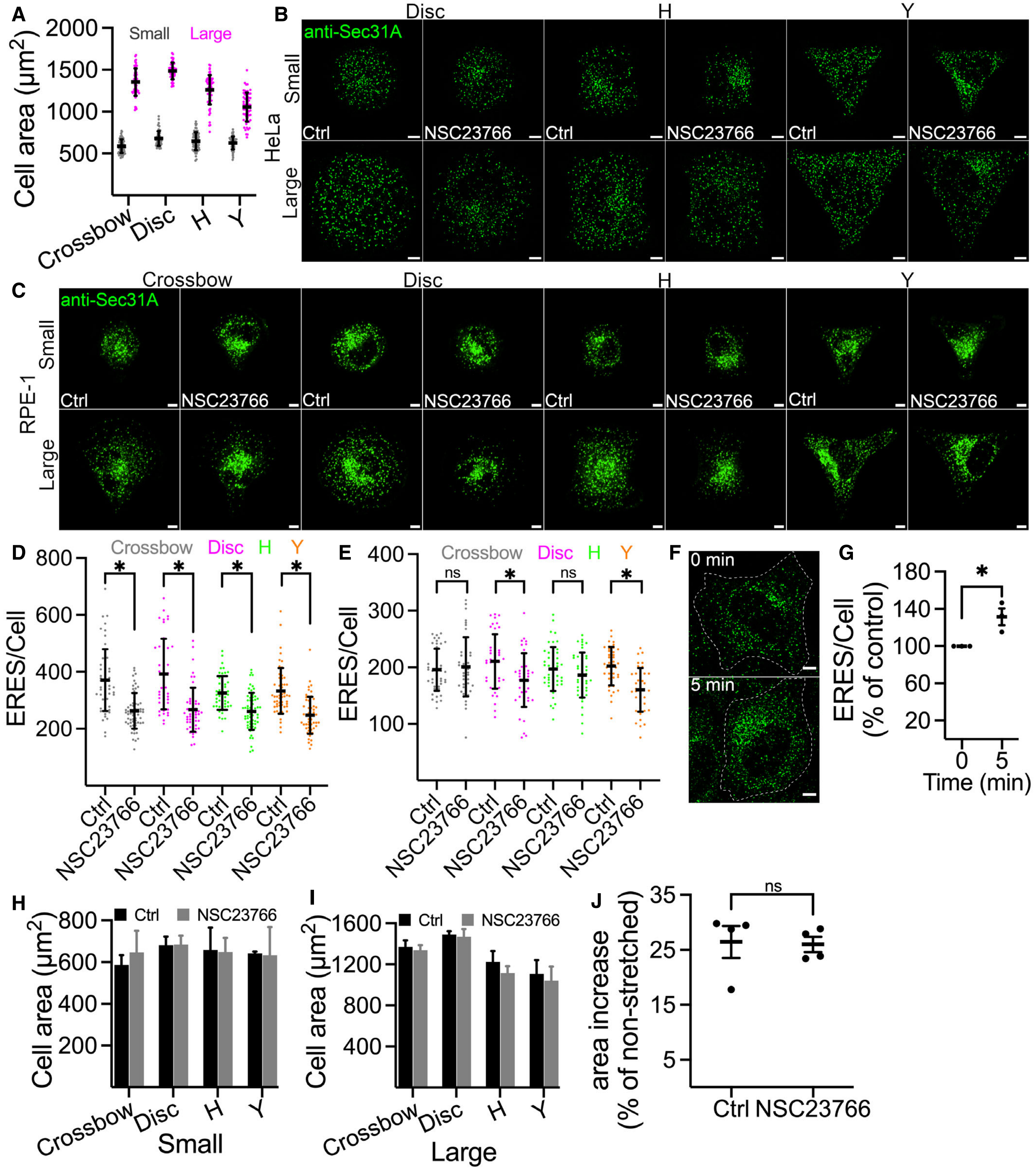


Figure EV1.



© 2022 The Authors

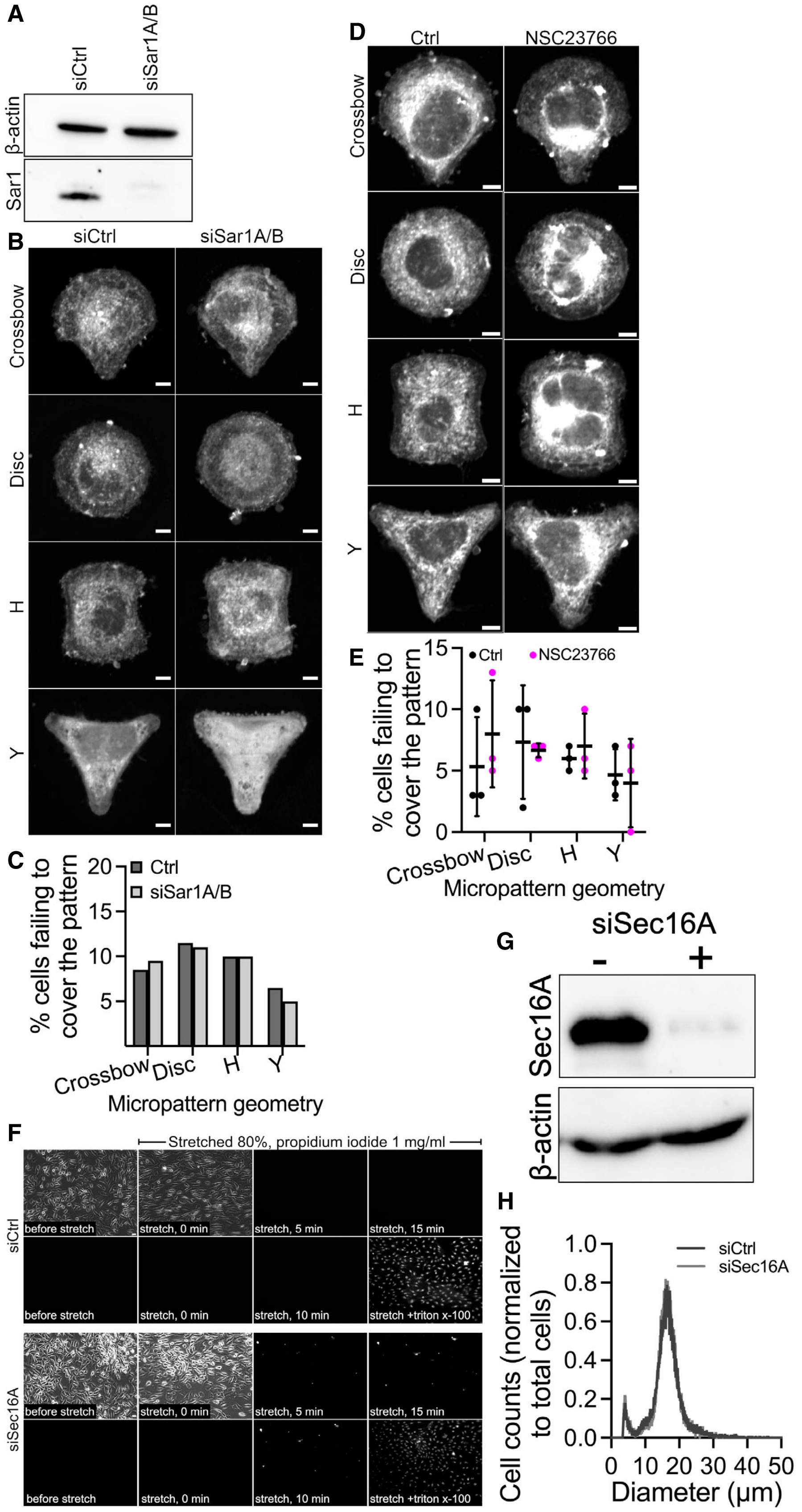


**Figure EV2. ERES function confers resistance of cell to mechanical stress.**

A HeLa cells were cotransfected with 5 nM siSar1A and 5 nM siSar1B (siSar1A/B) for 48 h. Control cells (siCtrl) were transfected with 10 nM non-targeting siRNA oligos. The efficiency of knockdown was evaluated by Western blot. A representative immunoblot showing the knockdown efficiency of siRNA oligos.

B Representative immunofluorescence images of HeLa cells cultured on micropatterned surface of multiple geometries of small size (600 μm2). Cells were treated as described in (A), and after 48 h, cells were trypsinized and 80,000 cells were seeded on micropatterned chips. Cells were allowed to attach for 3 h, stained with CellMask for 5 min, and processed for microscopy. Scale bars: 5 μm.

C Quantification graph showing the percentage of cells failing to entirely cover the different geometries (described in B). Between 60 and 70 cells were imaged in two independent experiments.

D Representative immunofluorescence images of HeLa cells cultured on micropatterned surface of multiple geometries of small size (600 μm2). Cells were treated with DMSO (Ctrl) or NSC23766 for 4 h before staining with CellMask for 5 min and processed for microscopy. Scale bars: 5 μm.

E Quantification graph showing the percentage of cells failing to entirely cover the different geometries (described in D). Between 98 and 106 cells were imaged in three independent experiments. Error bars represent standard deviation.

F Depletion of Sec16A compromises membrane integrity. HeLa cells were transfected with 5 nM non-targeting siRNA (Ctrl) or siRNA targeting Sec16A (siSec16A). After 48 h, cells were harvested by trypsinization and immediately seeded on fibronectin-coated silicon membranes. After 24 h, cells were unidirectionally stretched by 80% for 15 min in the presence of a membrane impermeable dye (propidium iodide). To visualize cells at the end of the experiment, tritonX-100 was added. Scale bar: 50 μm.

Source data are available online for this figure.

© 2022 The Authors

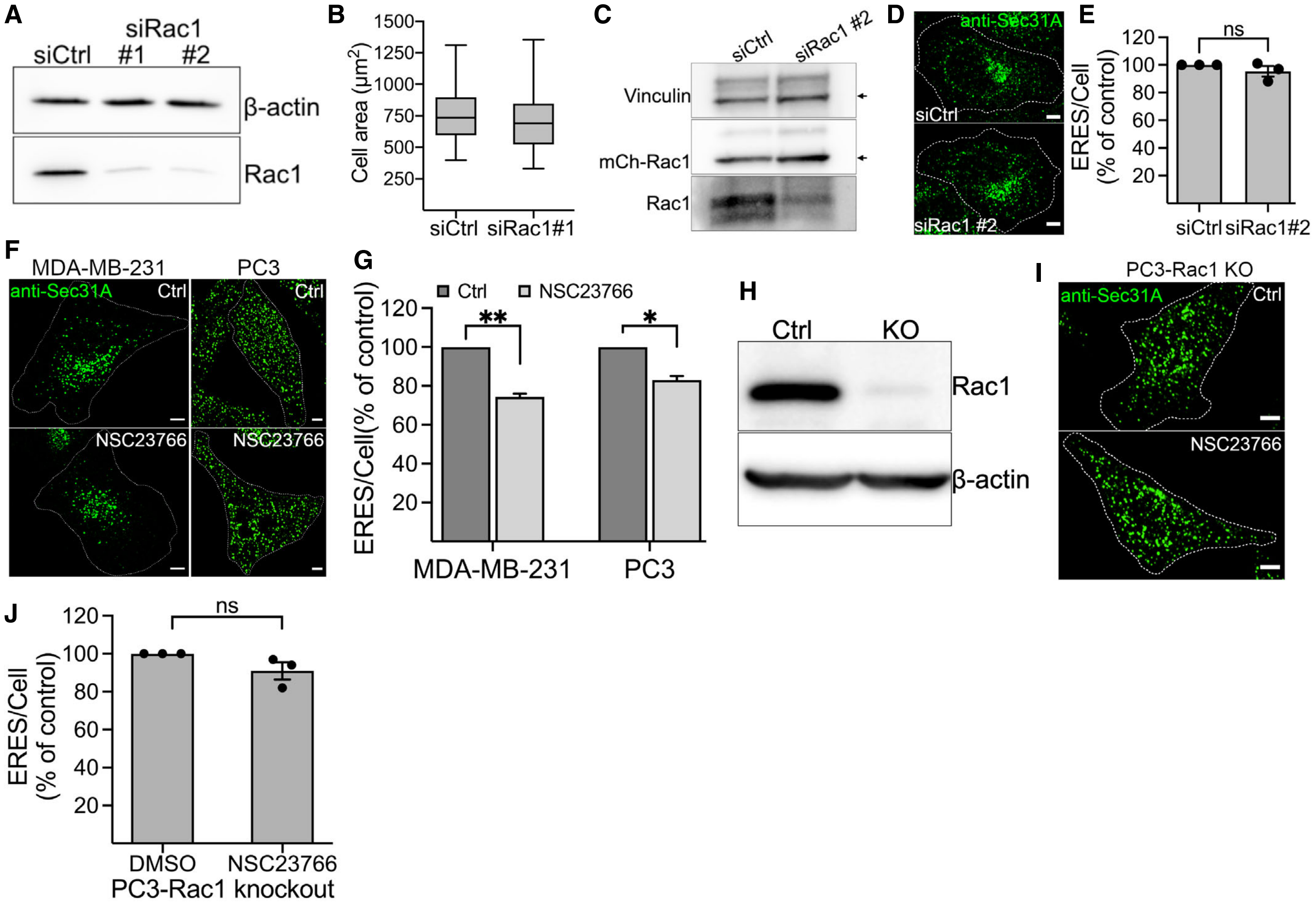


**Figure EV3. Rac1 regulates ERES.**

A HeLa cells were transfected with two siRNAs against Rac1 (siRac1 #1 and siRac1 #2) for 72 h. The efficiency of knockdown was evaluated by Western blot. A representative immunoblot showing the knockdown efficiency of siRNA oligos.

B Area covered by HeLa cells transfected with non-targeting control siRNA (siCtrl) or siRNA against Rac1 (siRac1 #1). Number of cells = 30, number of experiments = 3. In the boxplot, whiskers indicate the min-max range of the data and the middle band indicates the median of all data.

C–E HeLa cells stably expressing mCherry-tagged siRNA resistant Rac1 was transfected with control siRNA (siCtrl) or siRNA against Rac1 (siRac1 #2) for 72 h. Cells were then lysed for immunoblotting or processed for immunostaining with Sec31A. (C) A representative immunoblot showing reduction in the levels of endogenous Rac1 while siRNA resistant mCherry-Rac1 remains unaffected. (D) Immunofluorescence images showing ERES in siCtrl and siRac1 treated cells. Scale bars, 5 μm. (E) Quantitation of experiment in D. Graph shows mean ± SEM (*n* = 3, siCtrl: 117 cells, siRac1 #2: 94 cells). ns = not siginificant.

F, G Inhibition of Rac1 with NSC23766 (50 μM for 4 h) reduces ERES in MDA-MB-231 and PC3 cells. Sec31A marked ERES are shown in F and quantification of ERES is shown in (F). Scale bars: 5 μm. (G) Graph shows mean ± SEM (*n* = 3 experiments, at least 30 cell/experiment), where asterisks (*) represent statistical significance at *P*-value <0.05, and two asterisks represent statistical significance at *P*-value <0.05 (Student's unpaired *t* test).

H Immunoblot of CRISPR Rac1 knockout PC3 cells.

I, J Effect of Rac1 inhibitor NSC23766 is on-target. CRISPR Rac1 knockout PC3 (PC3-Rac1 KO) cells were treated with DMSO (Ctrl) or NSC23766 (50 μM) for 4 h and ERES was quantified by counting Sec31A labeled puncta from confocal images. (I) Representative images showing ERES in PC3-Rac1 KO Ctrl or NSC23766 treated cells. Scale bars, 5 μm. (J) Treating PC3-Rac1 KO cells with NSC23766 does not reduce ERES. Average ERES per cell are displayed in % of control (*n* = 3, number of cells = 60–90). ns = nonsignificant.

Source data are available online for this figure.

 © 2022 The Authors

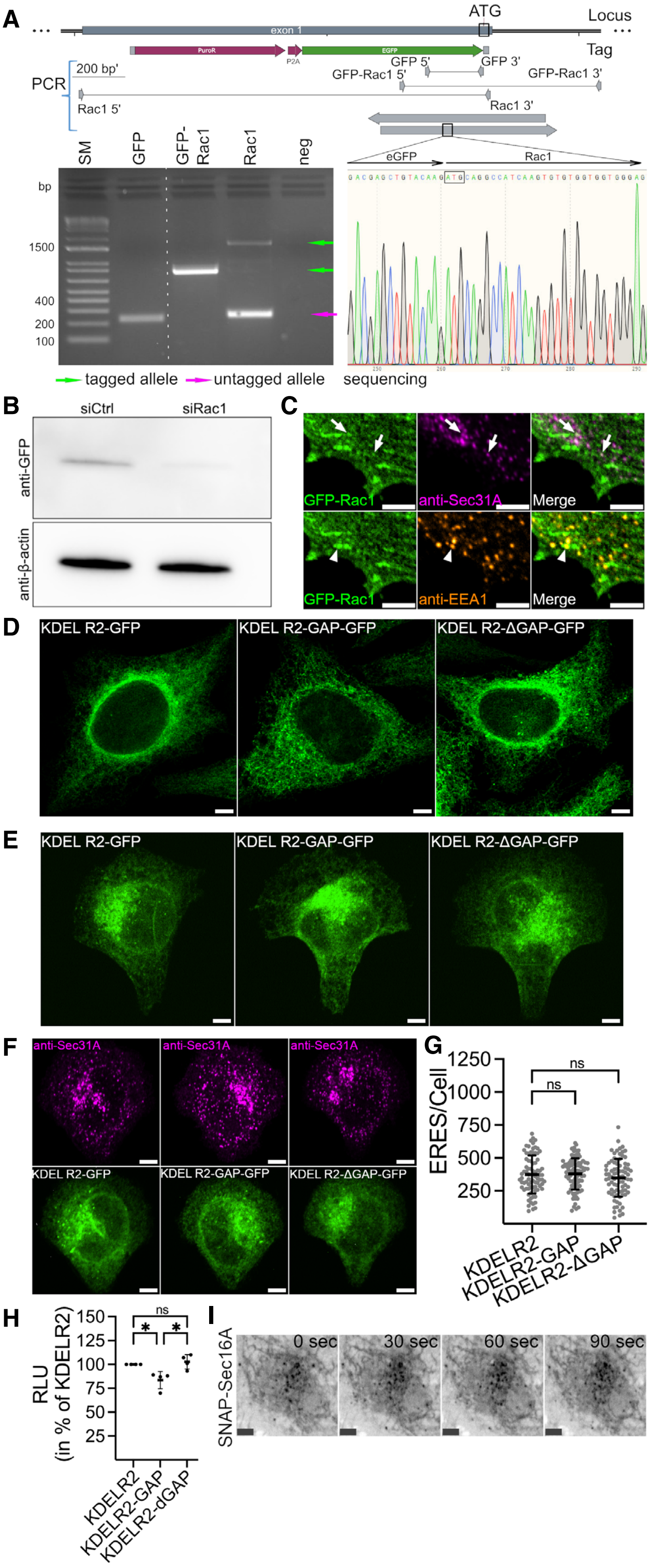

**Figure EV4. Activity of Rac1 at the ER regulates ERES.**

A CRISPR/Cas system was used to endogenously tag Rac1 in HeLa cells with GFP. PCR and Sanger sequencing approaches were used to validate successful incorporation of GFP at the desired location. Schematic illustration of GFP knock-in strategy and binding sites for primers (top). DNA agarose gel showing PCR fragments (bottom left) and sequencing chromatogram showing sequencing results (bottom right).

B HeLa cells generated in (A) were transiently transfected with 10 nM siRNA targeting Rac1 (siRac1) for 72 h prior to cell lysis and anti-GFP immunoblotting. Control (siCtrl) cells were transfected with 10 nM nontargeting siRNA oligos. A representative immunoblot showing levels of GFP-Rac1 and β-actin is shown.

C HeLa cells generated in (A) were co-immunostained with anti-Sec31A and anti-EEA1 antibodies. Representative immunofluorescence image is shown. Arrows indicate co-occurrence of GFP-Rac1 and Sec31A. Note the absence of anti-EEA1 staining. Arrowhead shows co-occurrence of GFP-Rac1 and EEA1. Scale bars, 5 μm.

D, E Representative immunofluorescence images show expression of the indicated plasmids in HeLa cells cultured on normal glass coverslips (D) or crossbow micropatterns (E). Scale bars, 5 μm.

F HeLa cells were transfected with KDELR2-GFP, KDELR2-GAP-GFP or KDELR2-ΔGAP-GFP plasmids and incubated overnight. At the end of the incubation, cells were harvested by trypsinization and 80,000 cells were cultured on fibronectin-coated micropatterns. Cells were allowed to attach for 4 h before fixation and Sec31A immunostaining. Representative immunofluorescence images showing Sec31A marked ERES (upper panel) and plasmid expression (lower panel) in cells covering small crossbow geometry. Scale bars, 5 μm.

G Quantification of (F). ERES in 79–91 cells were counted in three experiments. ns = nonsignificant, one-way ANOVA was used to test for statisticial significance. Error bars show standard deviation.

H Quantification graph shows relative luminescence values obtained from HeLa cells that stably express KDELR2-mCherry, KDELR2-GAP-mCherry or KDELR2-ΔGAP-mCherry. Cells were lysed and the level of active Rac1 was determined using Rac1 G-LISA Activation Assay kit following the manufacturer's protocol. Luminescence values obtained from four independent experiments are presented as % of KDELR2-mCherry (KDELR2) expressing cells. **P*-value <0.05, one-way ANOVA. ns = nonsignificant. Error bars show standard deviation.

I SNAP-Sec16A distribution in HeLa cells at indicated time points after light-induced recruitment of TIAM to the ER (see Fig 4I and J). Scale bar: 5 μm.

Source data are available online for this figure.

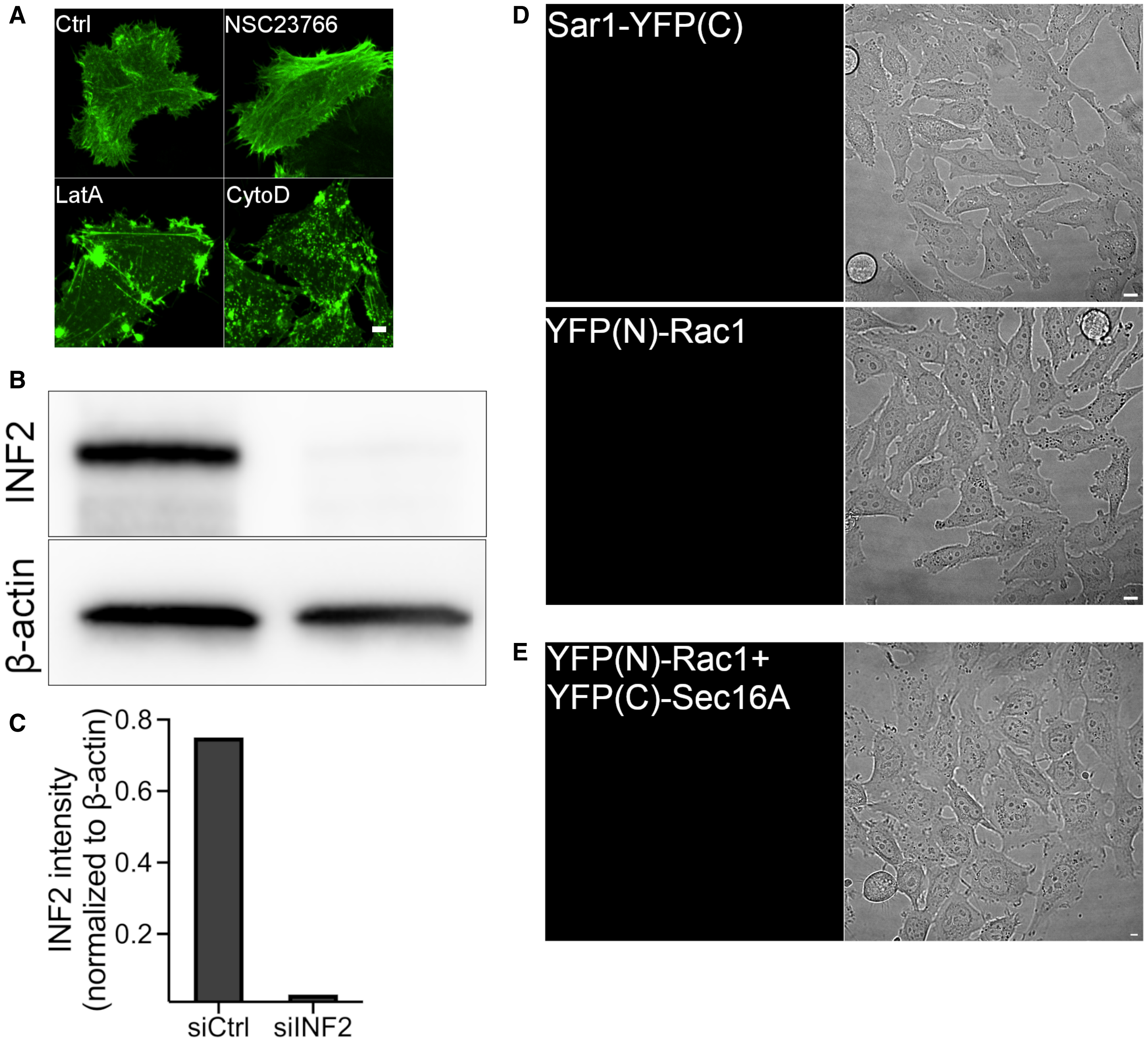


**Figure EV5. Controls for actin disruption and YFP-BiFC.**

A Treatment of HeLa cells expressing GFP-tagged actin chromobody with 0.5 μM cytochalasin D (CytoD) or latrunculin A (LatA) for 15 min severely affects actin compared to DMSO (Ctrl) or Rac1 inhibitor (NSC23766, 50 μM for 4 h).

B, C Immunoblot (B) and quantification graph (C) showing reduction in endogenous INF2 level in HeLa cells following transfection with 25 nM siRNA targeting INF2 (siINF2) for 72 h.

D When expressed alone, Sar1-YFP(C) and YFP(N)-Rac1 show no YFP signal.

E YFP(N)-Rac1 and YFP(C)-Sec16 cotransfected HeLa cells. Lack of positive YFP signal suggest no interaction of Rac1 and Sec16.

Data information: Scale bar for immunofluorescence images (A, D, and E): 5 μm.
Source data are available online for this figure.


© 2022 The Authors
