# Supplementary material for: Mechanical strain stimulates COPII‐dependent secretory trafficking via Rac1
Source: EMBO J. 2022 Aug 8;41(18):e110596. doi: 10.15252/embj.2022110596 (PMC9475550; doi:10.15252/embj.2022110596)
Supplement: Supplementary file 2 — Movie EV1 [file EMBJ-41-e110596-s001.zip › Movie EV1.docx]

Movie EV1. ERES dynamics in HeLa cells before and during equibiaxial strain.

Timelapse movie corresponding to Fig. 1C. Frames were captured every 500 ms.
