# Supplementary material for: Mechanical strain stimulates COPII‐dependent secretory trafficking via Rac1
Source: EMBO J. 2022 Aug 8;41(18):e110596. doi: 10.15252/embj.2022110596 (PMC9475550; doi:10.15252/embj.2022110596)
Supplement: Supplementary file 3 — Movie EV2 [file EMBJ-41-e110596-s007.zip › Movie EV2.docx]

Movie EV2. ManII-RUSH trafficking in HeLa cells seeded on PDMS membranes.

Timelapse movie corresponding to Fig. 1H. Frames were captured every 90 sec over the course of 30 min.
