# Supplementary material for: Mechanical strain stimulates COPII‐dependent secretory trafficking via Rac1
Source: EMBO J. 2022 Aug 8;41(18):e110596. doi: 10.15252/embj.2022110596 (PMC9475550; doi:10.15252/embj.2022110596)
Supplement: Supplementary file 4 — Movie EV3 [file EMBJ-41-e110596-s005.zip › Movie EV3.docx]

Movie EV3. A transient pool of Rac1 at the ER.

Timelapse movie corresponding to Fig. 3A, showing co-occurence of Rac1 (magenta) with ER (labelled with GFP-Sec61β). 15 sec/frame for 3 min 45 secs.
