# Supplementary material for: Mechanical strain stimulates COPII‐dependent secretory trafficking via Rac1
Source: EMBO J. 2022 Aug 8;41(18):e110596. doi: 10.15252/embj.2022110596 (PMC9475550; doi:10.15252/embj.2022110596)
Supplement: Supplementary file 5 — Movie EV4 [file EMBJ-41-e110596-s012.zip › Movie EV4.docx]

Movie EV4. Rac1 co-occurs with GFP-Sec16A positive structures

Movie corresponding to Fig. 5A shows Rac1 (magenta) co-occuring with GFP-Sec16 labelled ERES. Frames were acquired every 5 sec over the course of 75 secs.
