# Supplementary figures and images for: Mechanical strain stimulates COPII‐dependent secretory trafficking via Rac1
Source: EMBO J. 2022 Aug 8;41(18):e110596. doi: 10.15252/embj.2022110596 (PMC9475550; doi:10.15252/embj.2022110596)

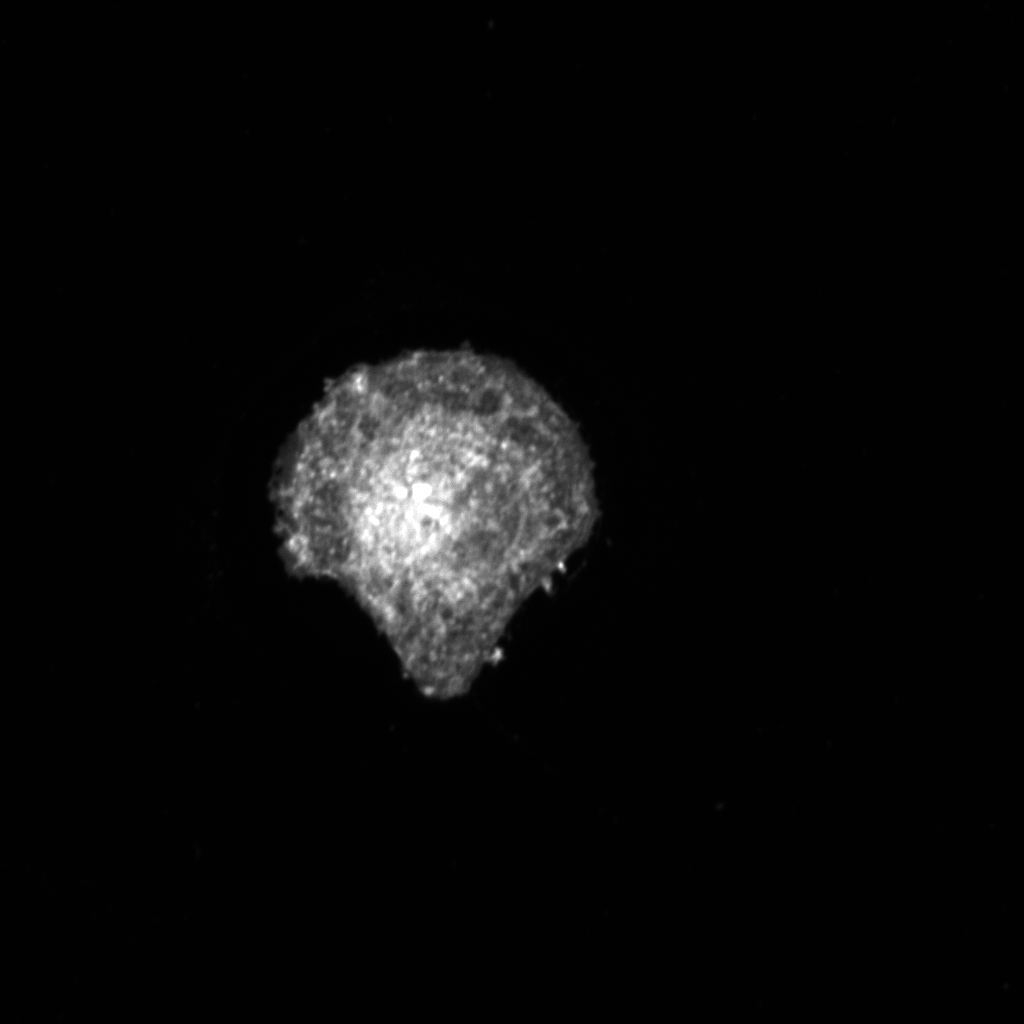

Supplement: Supplementary file 6 — Source Data for Expanded View [file EMBJ-41-e110596-s006.zip › Data/FigEV2B-siCtrl-Crossbow.tif]

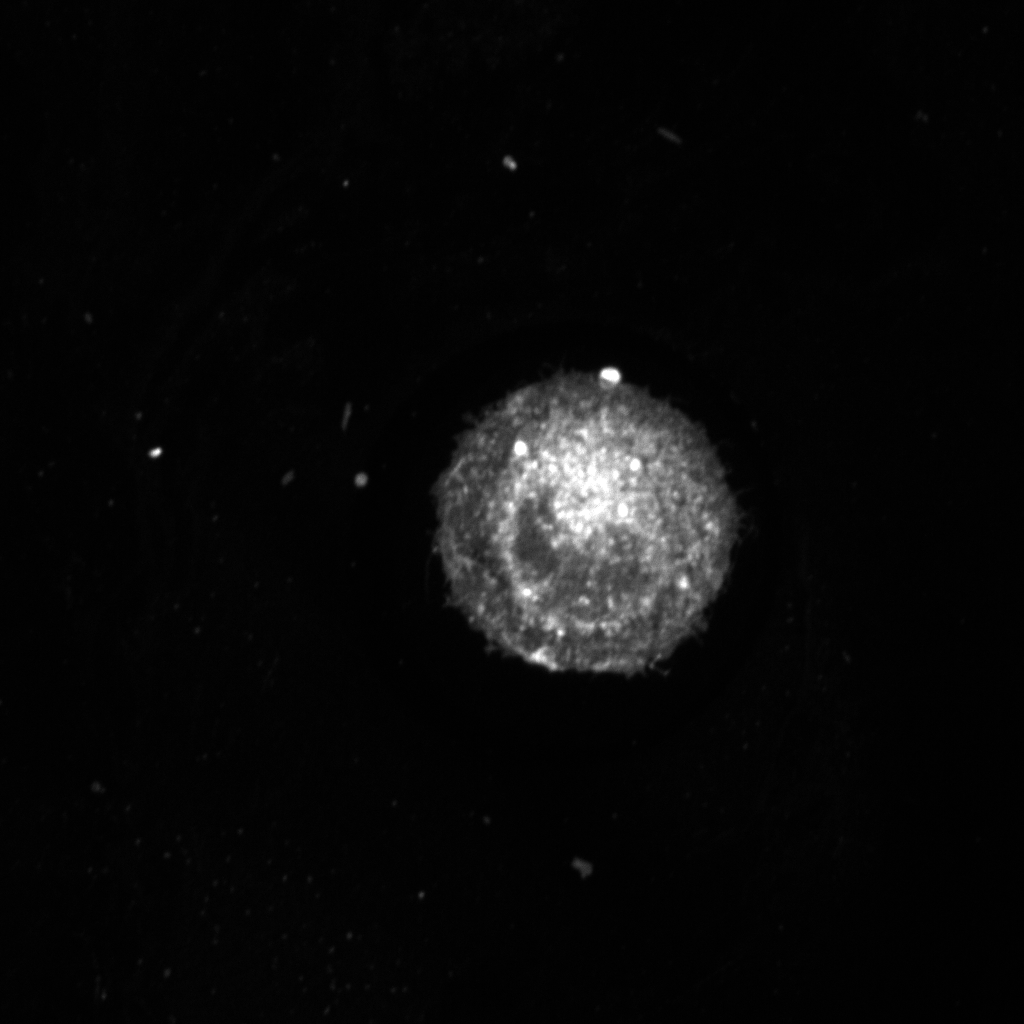

Supplement: Supplementary file 6 — Source Data for Expanded View [file EMBJ-41-e110596-s006.zip › Data/FigEV2B-siCtrl-Disc.tif]

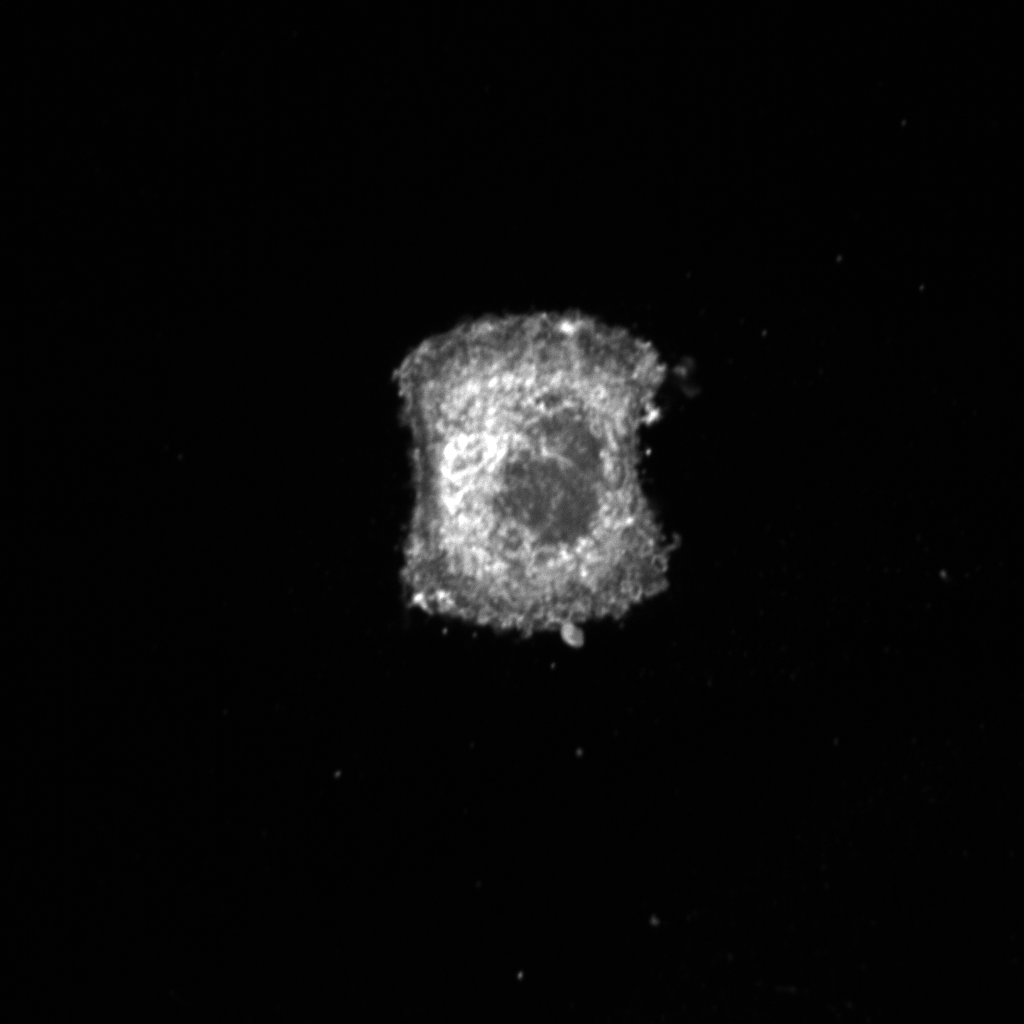

Supplement: Supplementary file 6 — Source Data for Expanded View [file EMBJ-41-e110596-s006.zip › Data/FigEV2B-siCtrl-I.tif]

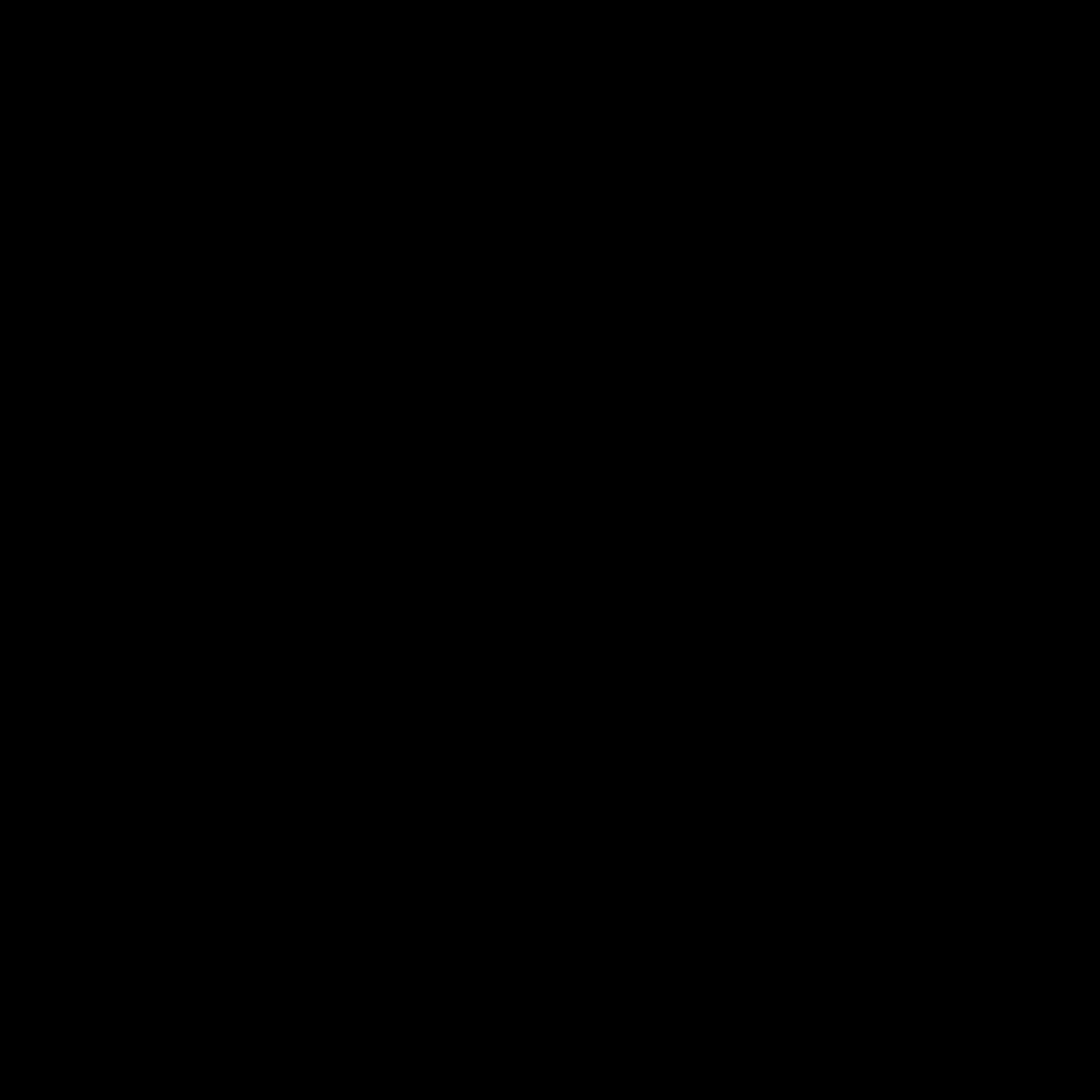

Supplement: Supplementary file 6 — Source Data for Expanded View [file EMBJ-41-e110596-s006.zip › Data/EV5E_YFP channel.tif]

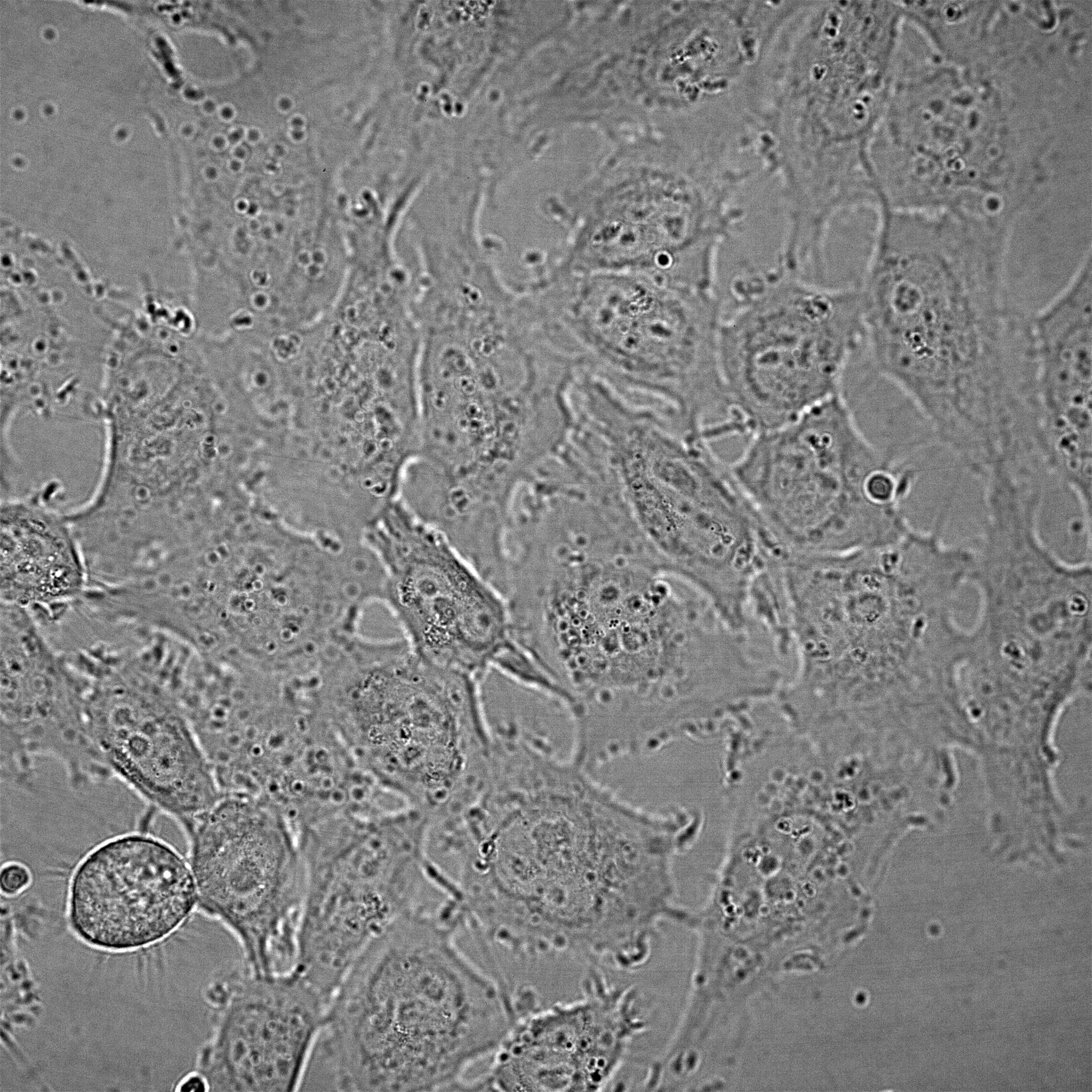

Supplement: Supplementary file 6 — Source Data for Expanded View [file EMBJ-41-e110596-s006.zip › Data/EV5E_Brightfield.tif]

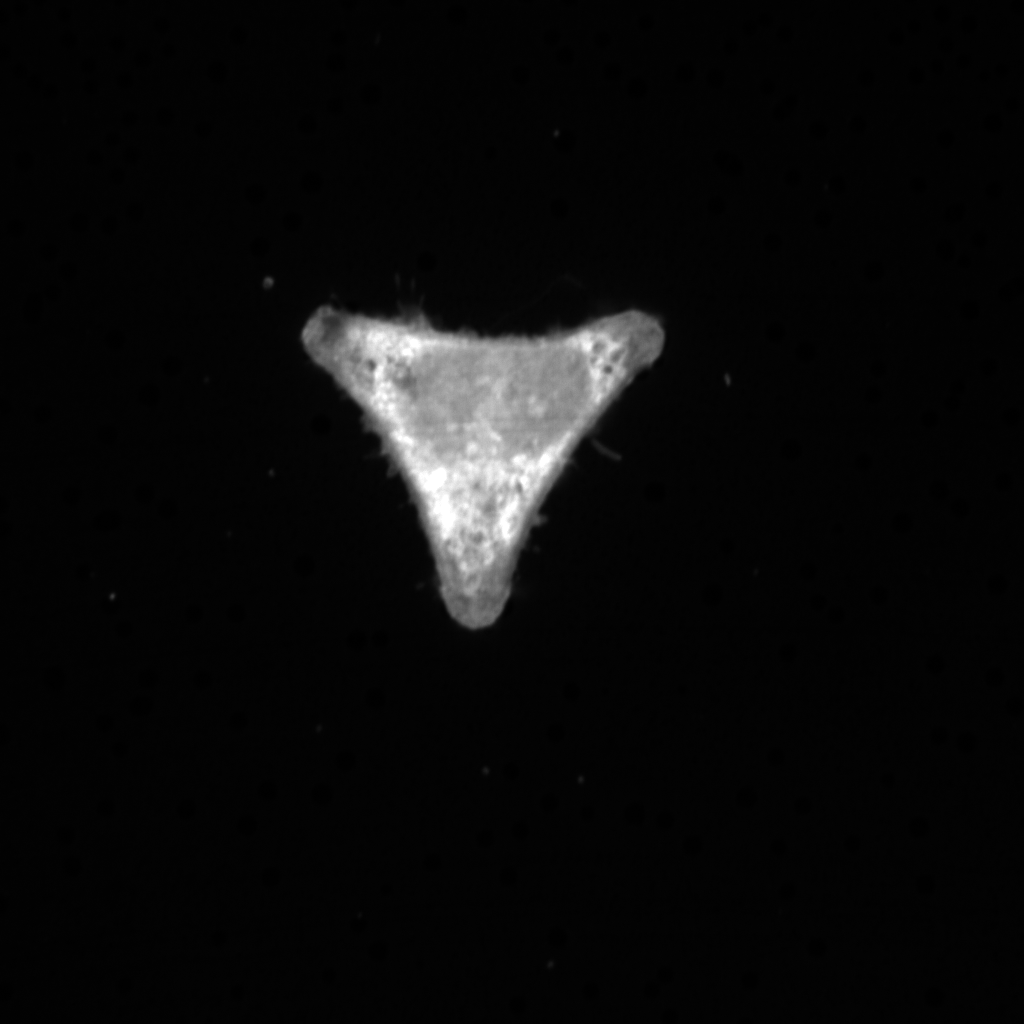

Supplement: Supplementary file 6 — Source Data for Expanded View [file EMBJ-41-e110596-s006.zip › Data/FigEV2B-siCtrl-Y.tif]

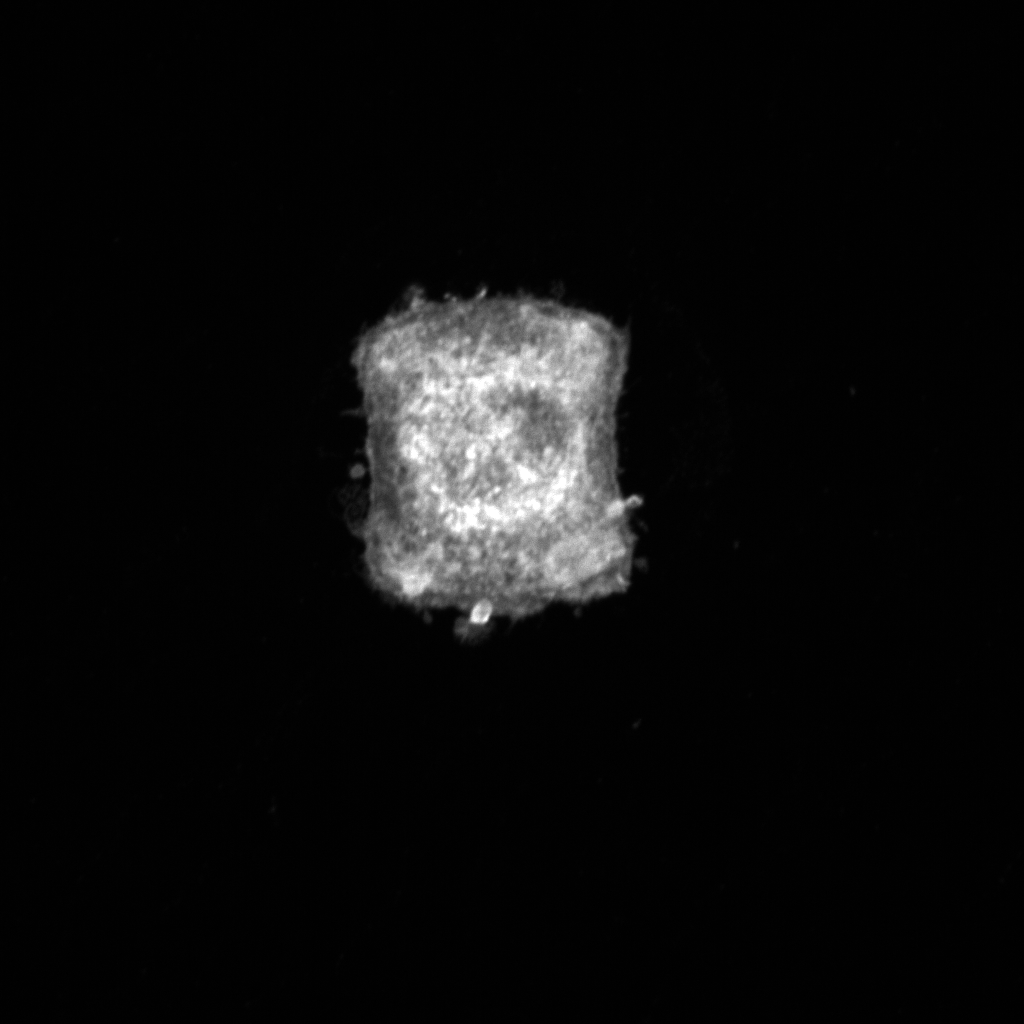

Supplement: Supplementary file 6 — Source Data for Expanded View [file EMBJ-41-e110596-s006.zip › Data/FigEV2B-siSar1AnB-I.tif]

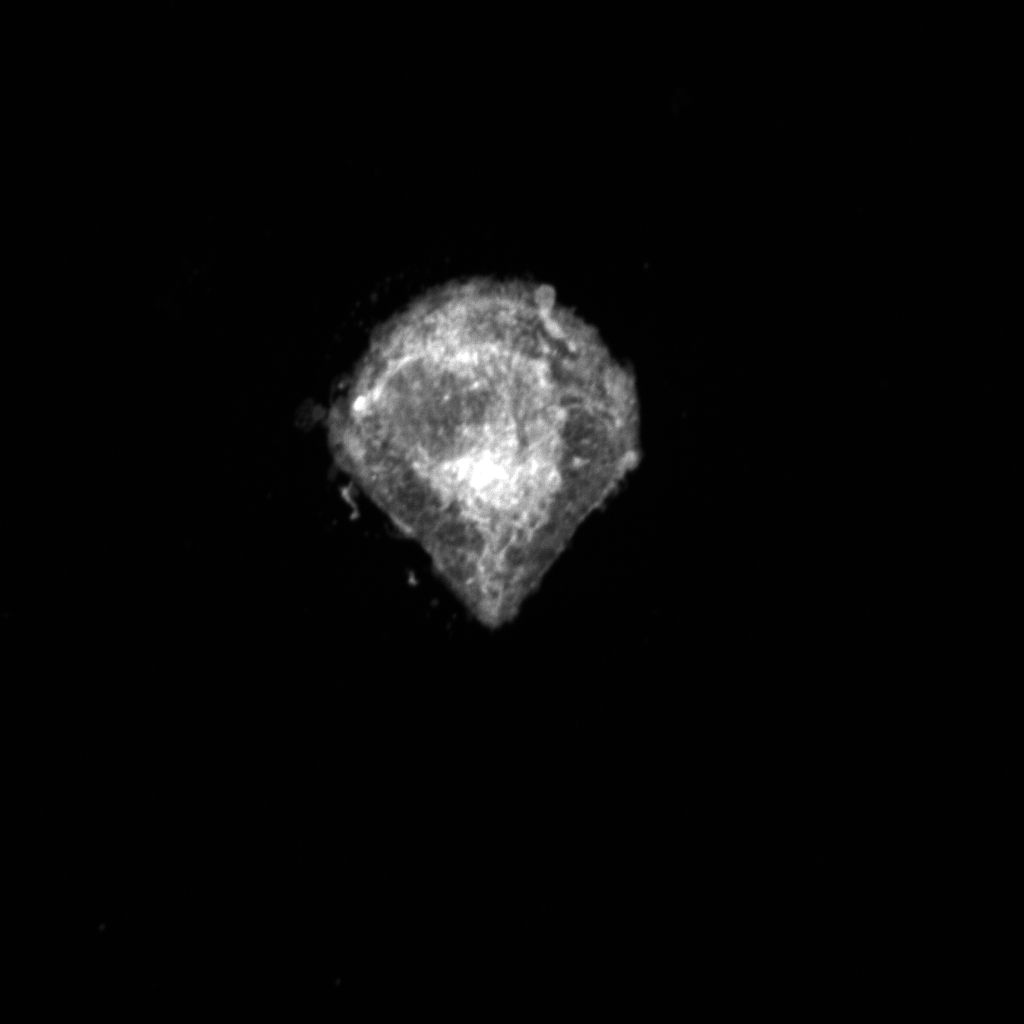

Supplement: Supplementary file 6 — Source Data for Expanded View [file EMBJ-41-e110596-s006.zip › Data/FigEV2B-siSar1AnB-Crossbow.tif]

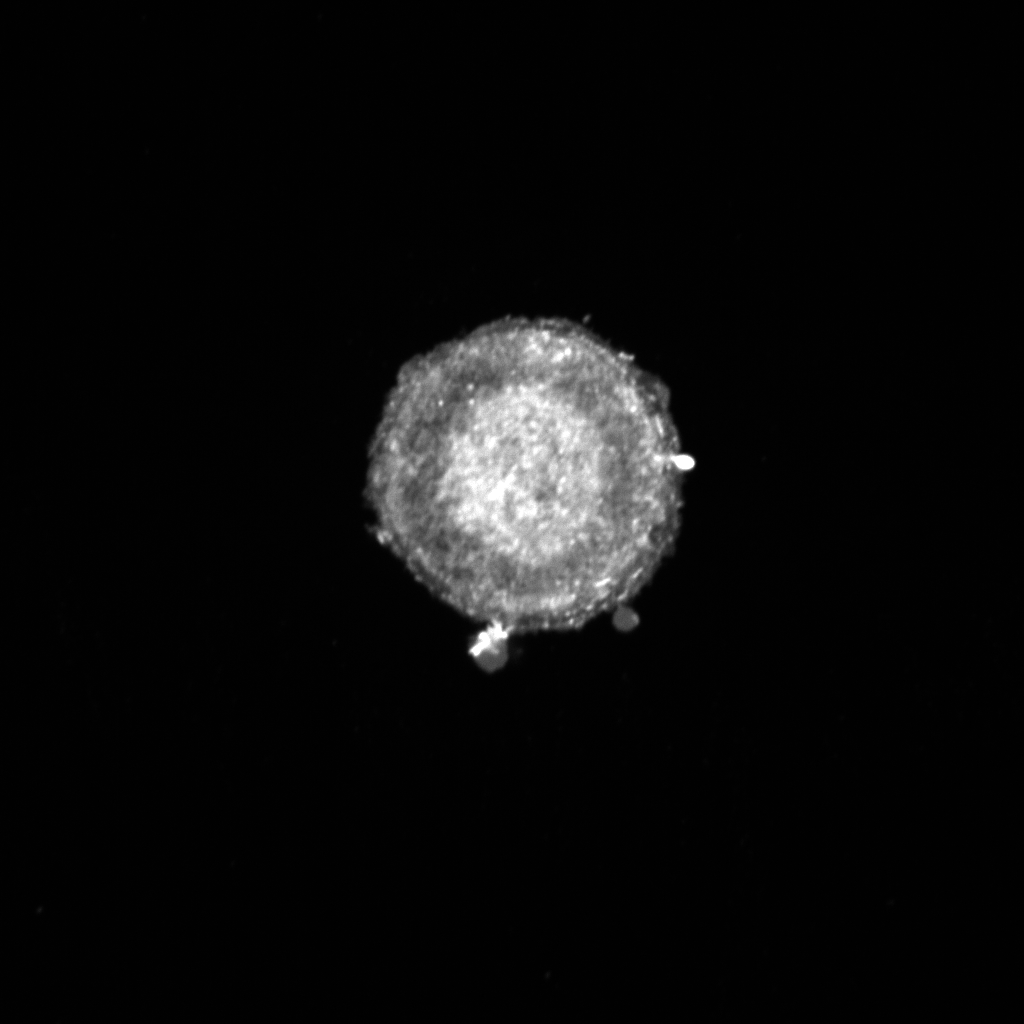

Supplement: Supplementary file 6 — Source Data for Expanded View [file EMBJ-41-e110596-s006.zip › Data/FigEV2B-siSar1AnB-Disc.tif]

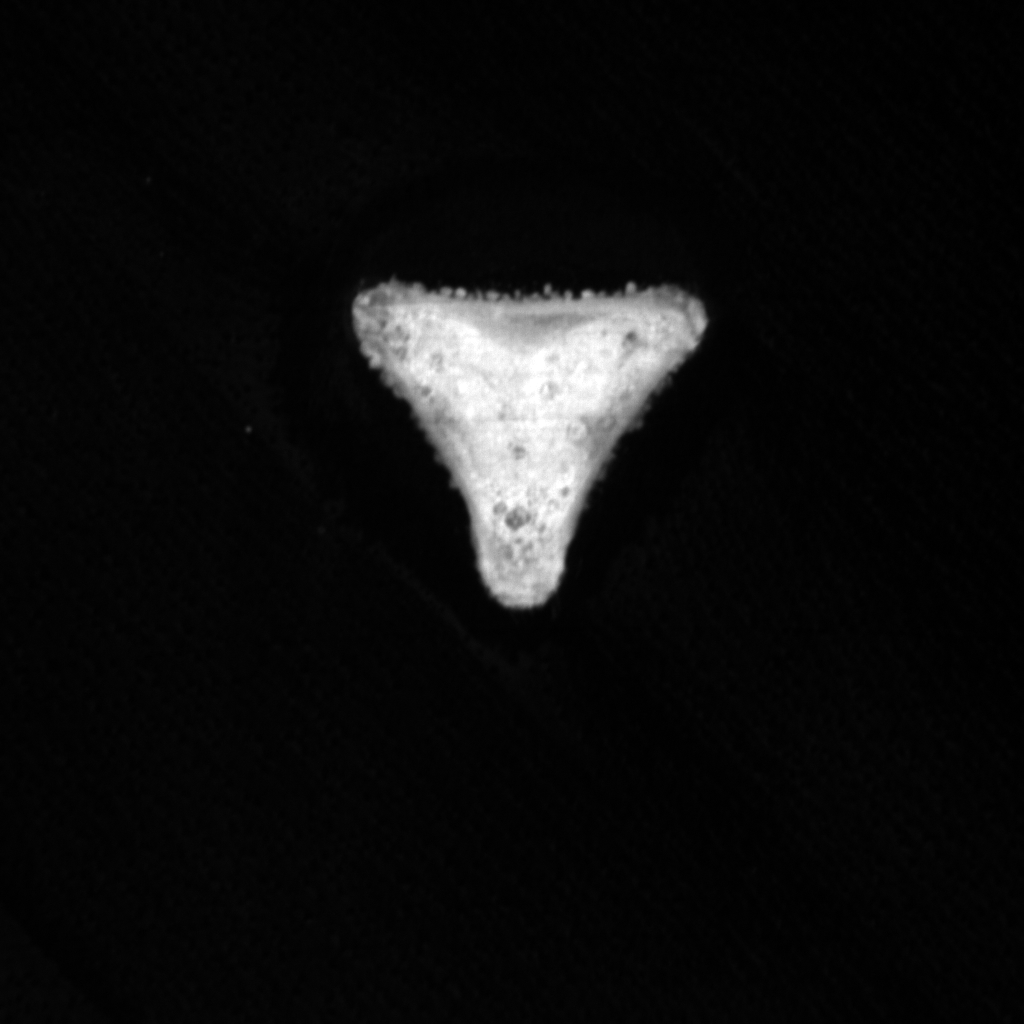

Supplement: Supplementary file 6 — Source Data for Expanded View [file EMBJ-41-e110596-s006.zip › Data/FigEV2B-siSar1AnB-Y.tif]

**Fig. EV2A**

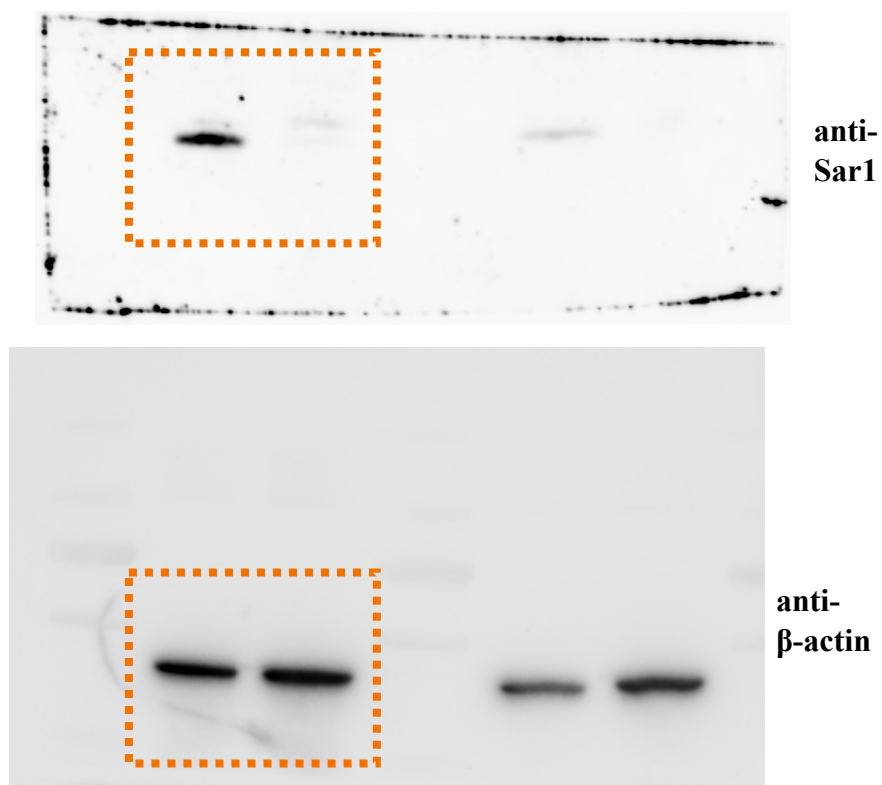

**Fig. EV2B**

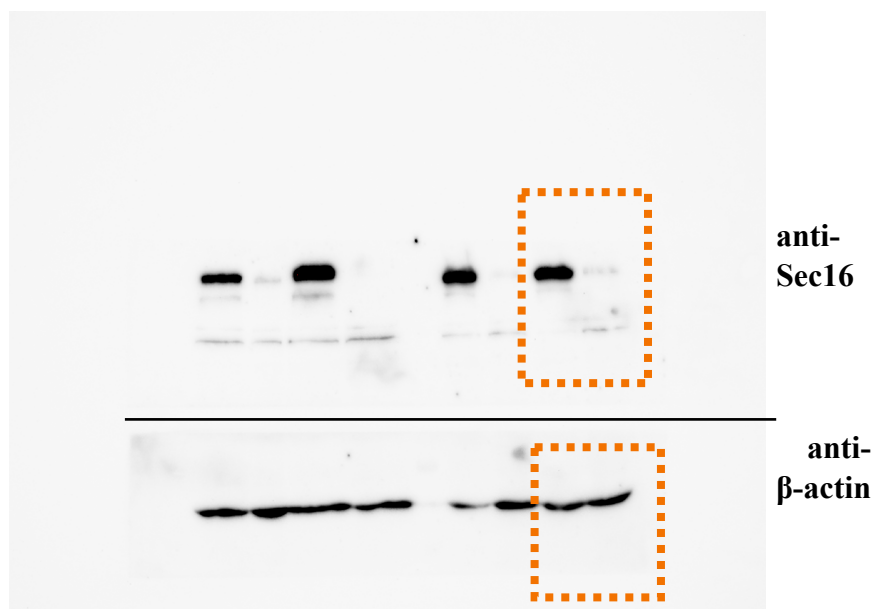

Supplement: Supplementary file 6 — Source Data for Expanded View [file EMBJ-41-e110596-s006.zip › Data/SD Fig EV2.pdf]

**Fig. EV3A**

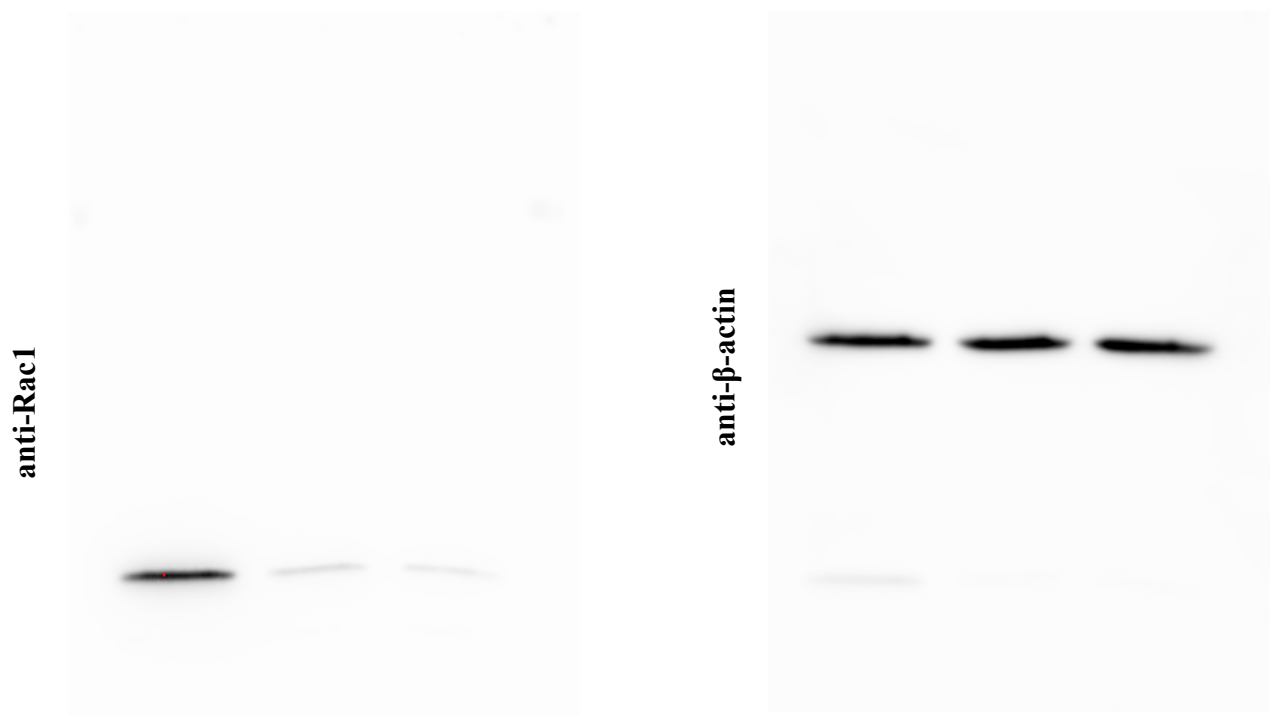

**Fig. EV3C**

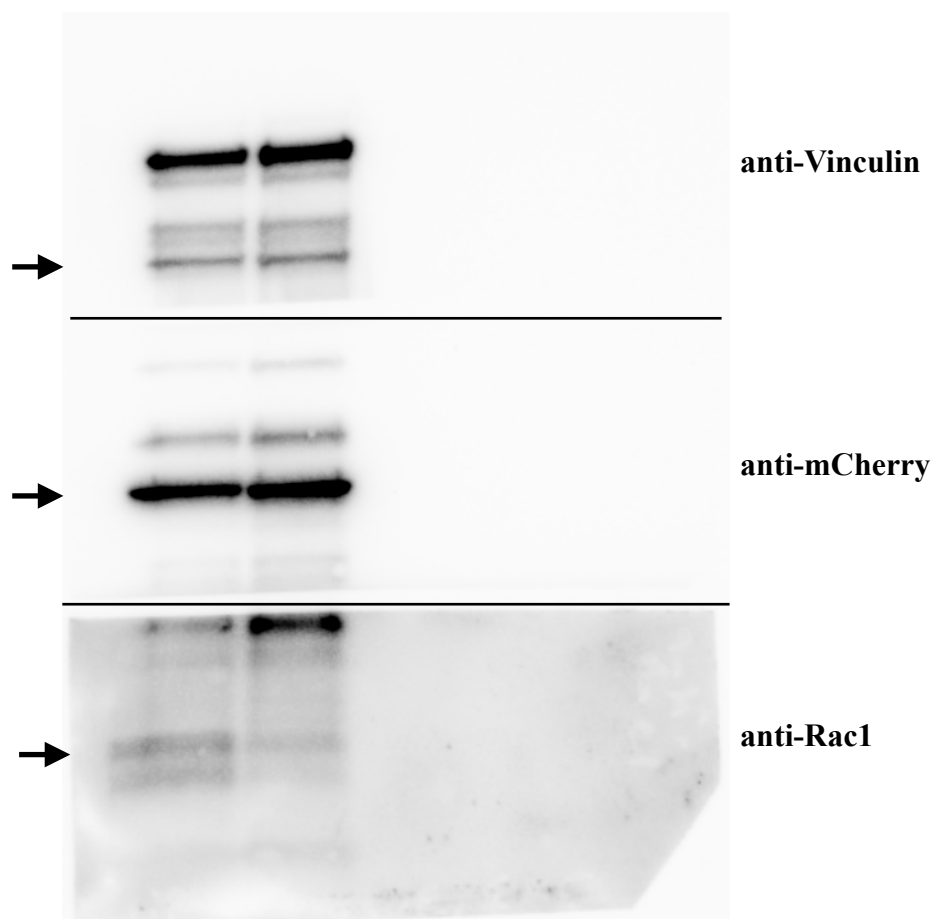

Supplement: Supplementary file 6 — Source Data for Expanded View [file EMBJ-41-e110596-s006.zip › Data/SD Fig EV3.pdf]

**Fig. EV4B**

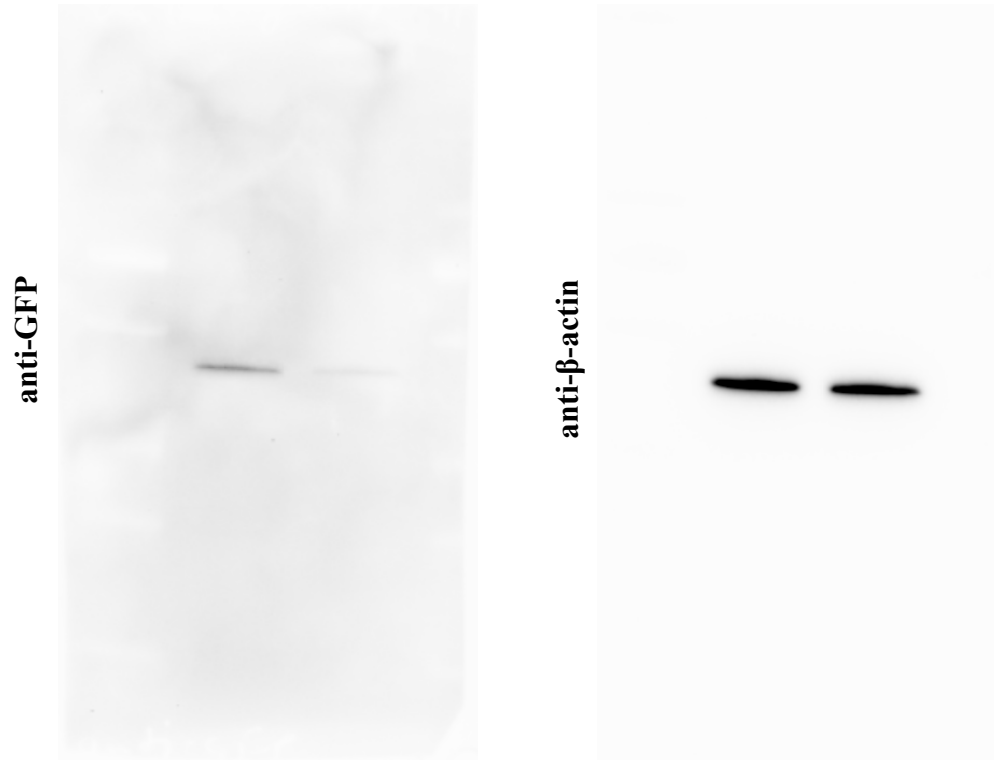

**Fig. EV5B**

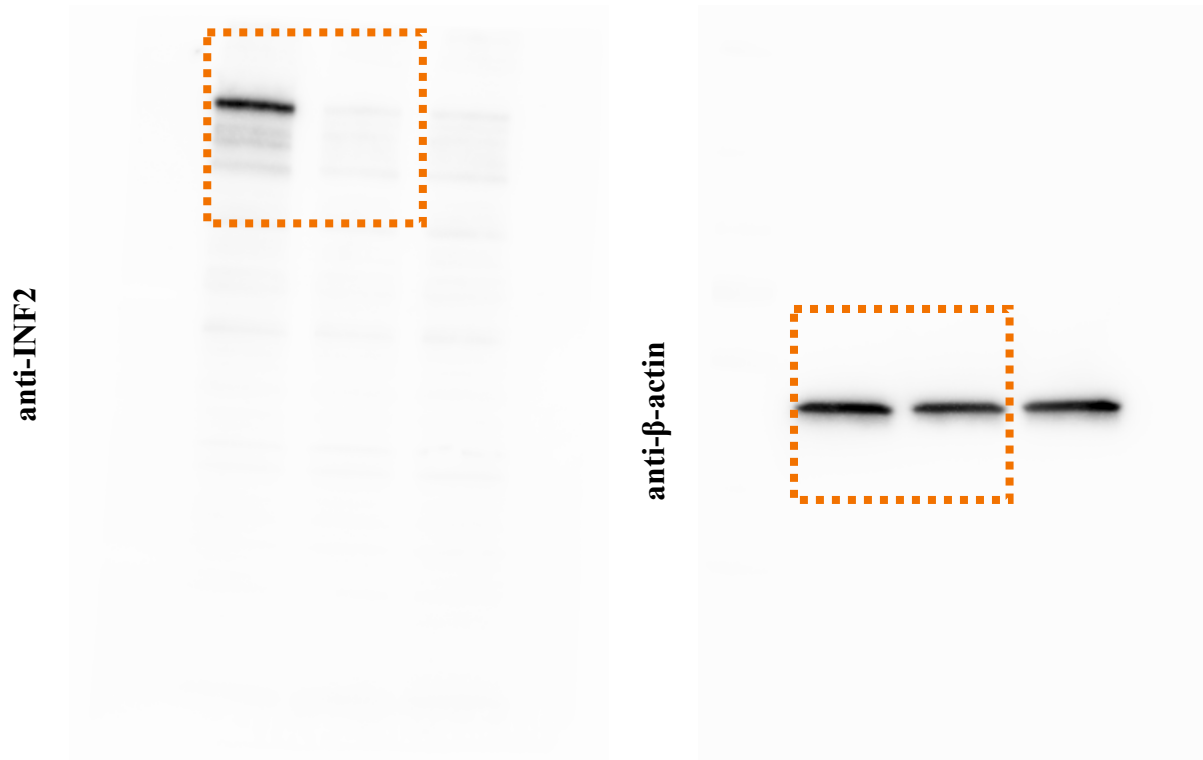

Supplement: Supplementary file 6 — Source Data for Expanded View [file EMBJ-41-e110596-s006.zip › Data/SD Fig EV4 & EV5.pdf]

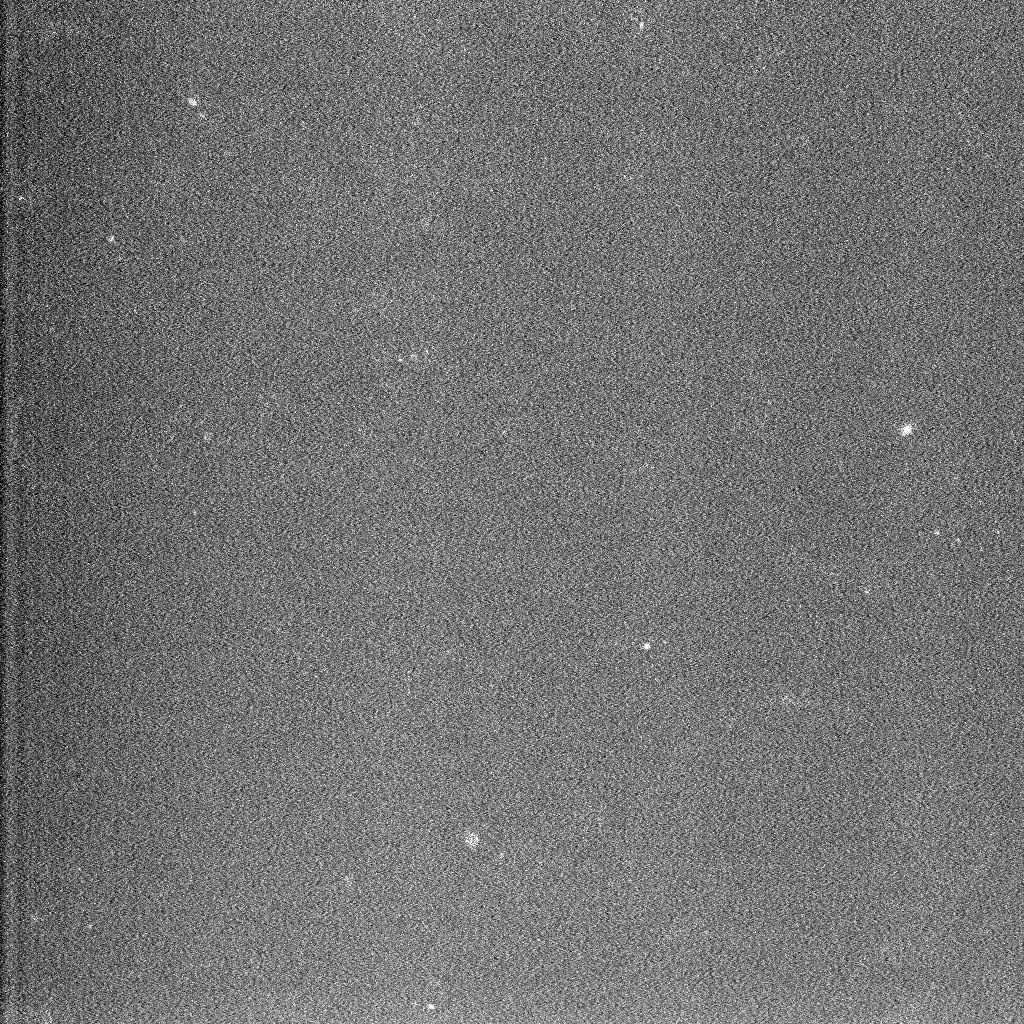

Supplement: Supplementary file 8 — Source Data for Figure 5 [file EMBJ-41-e110596-s008.zip › Fig5D-Sar1-panel-YFP-channel.tif]

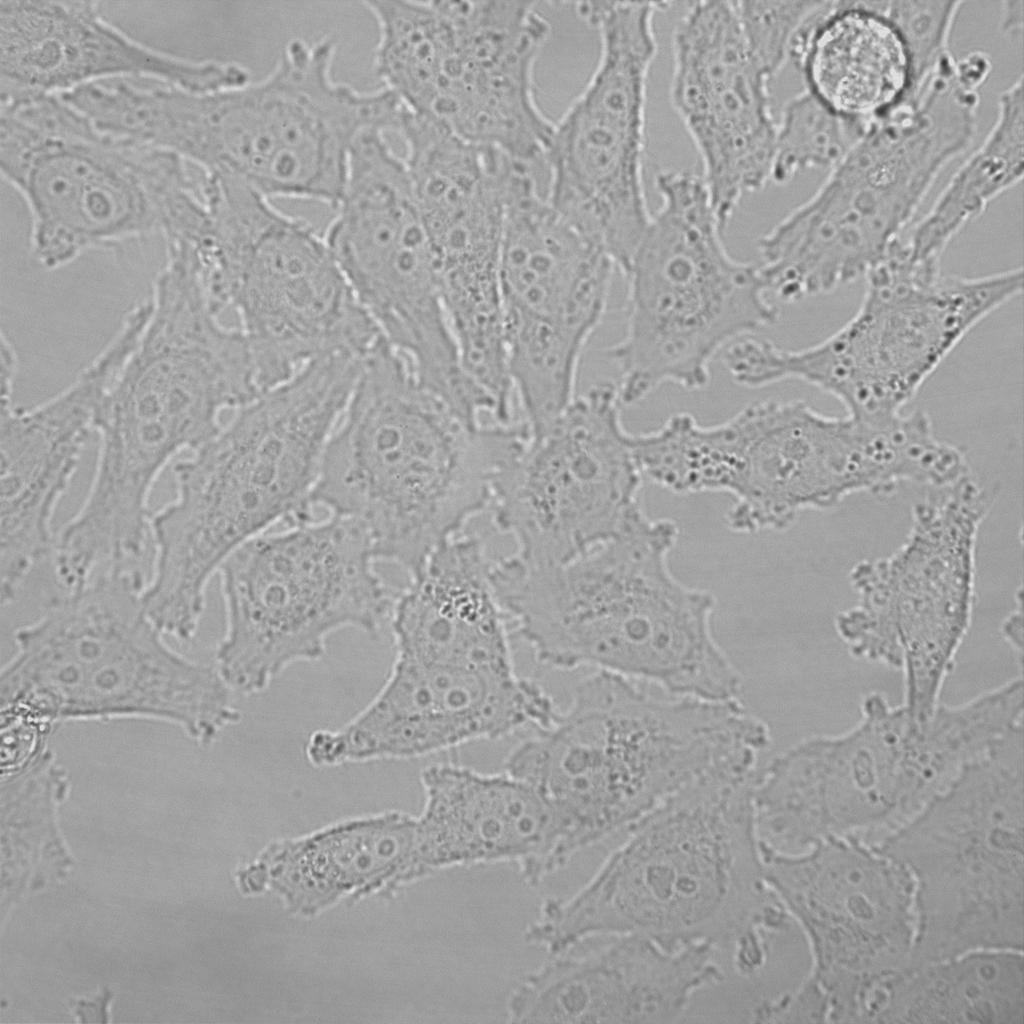

Supplement: Supplementary file 8 — Source Data for Figure 5 [file EMBJ-41-e110596-s008.zip › Fig5D-Rac1-panel-Brightfield.tif]

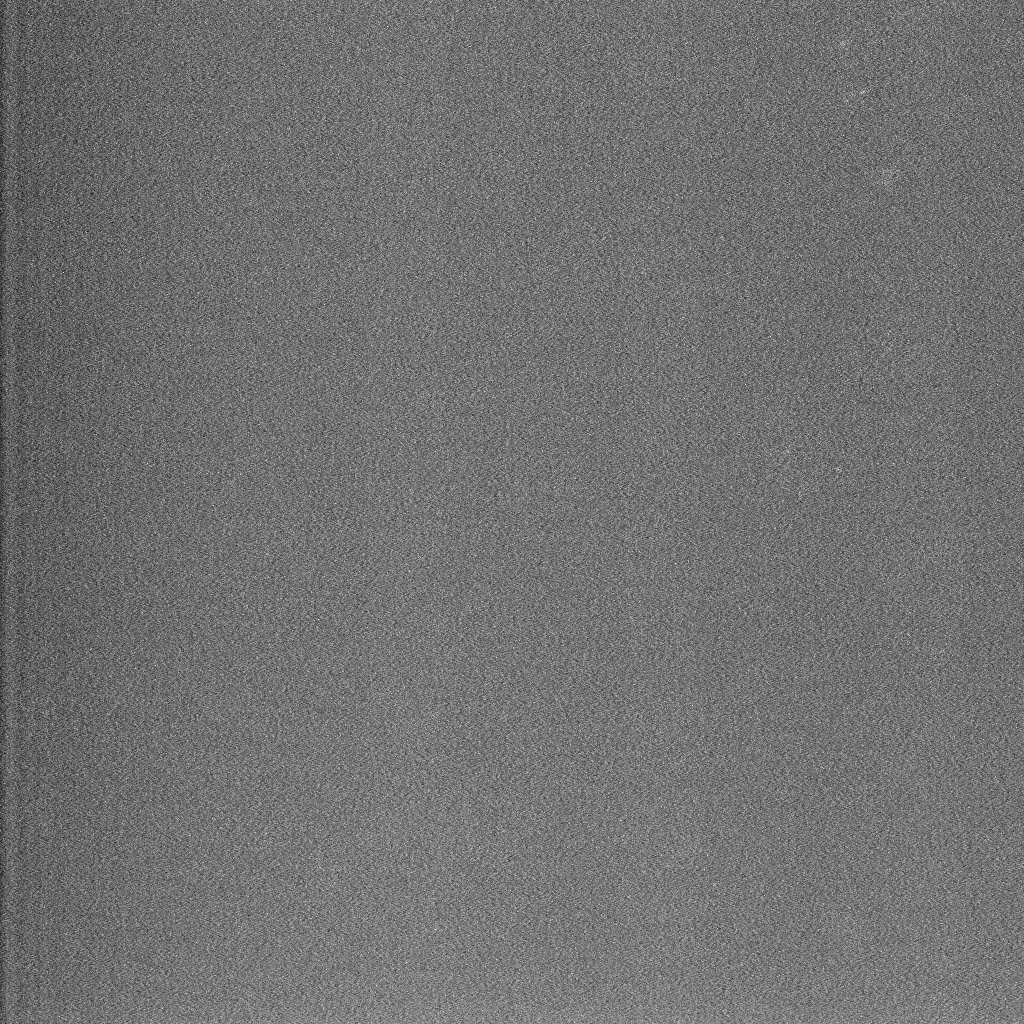

Supplement: Supplementary file 8 — Source Data for Figure 5 [file EMBJ-41-e110596-s008.zip › Fig5D-Rac1-panel-YFP-channel.tif]

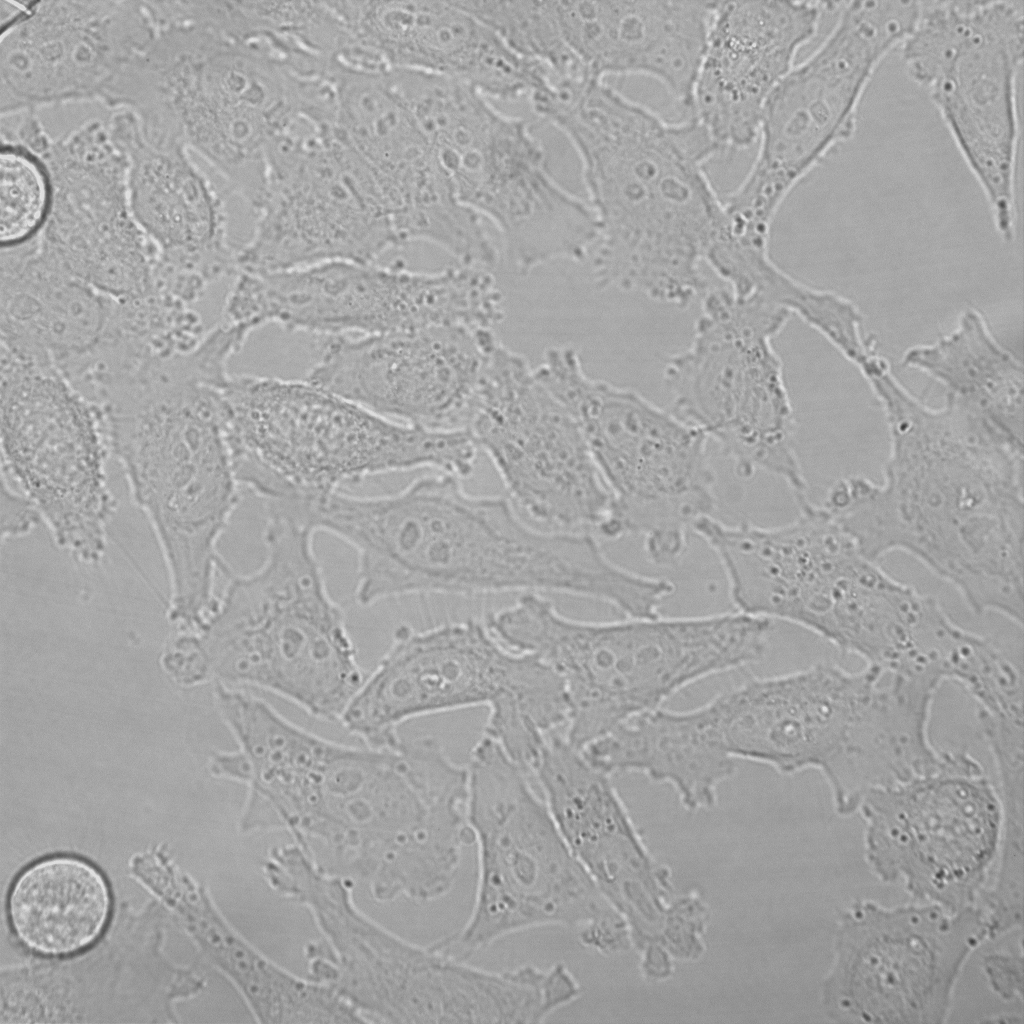

Supplement: Supplementary file 8 — Source Data for Figure 5 [file EMBJ-41-e110596-s008.zip › Fig5D-Sar1-panel-Brightfield.tif]

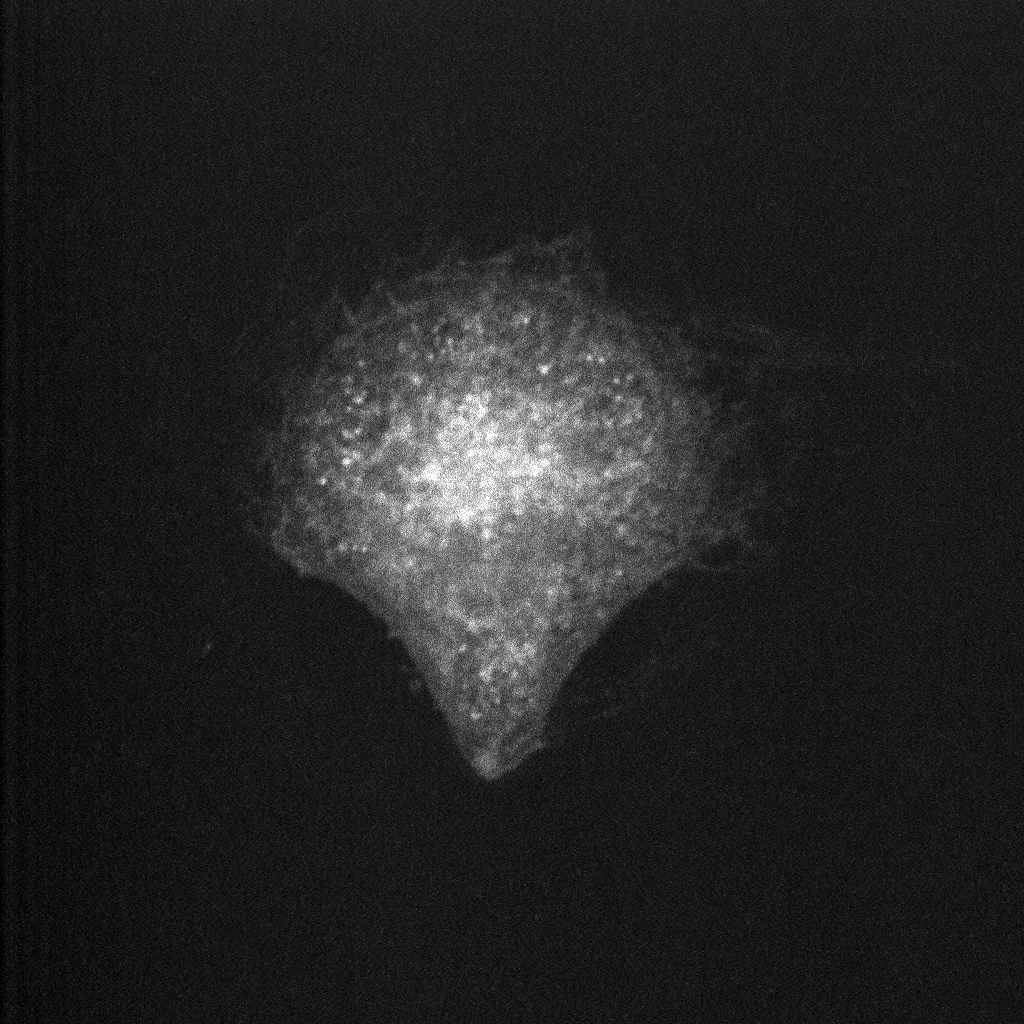

Supplement: Supplementary file 9 — Source Data for Figure 6 [file EMBJ-41-e110596-s003.zip › Data/Fig6E_Large.tif]

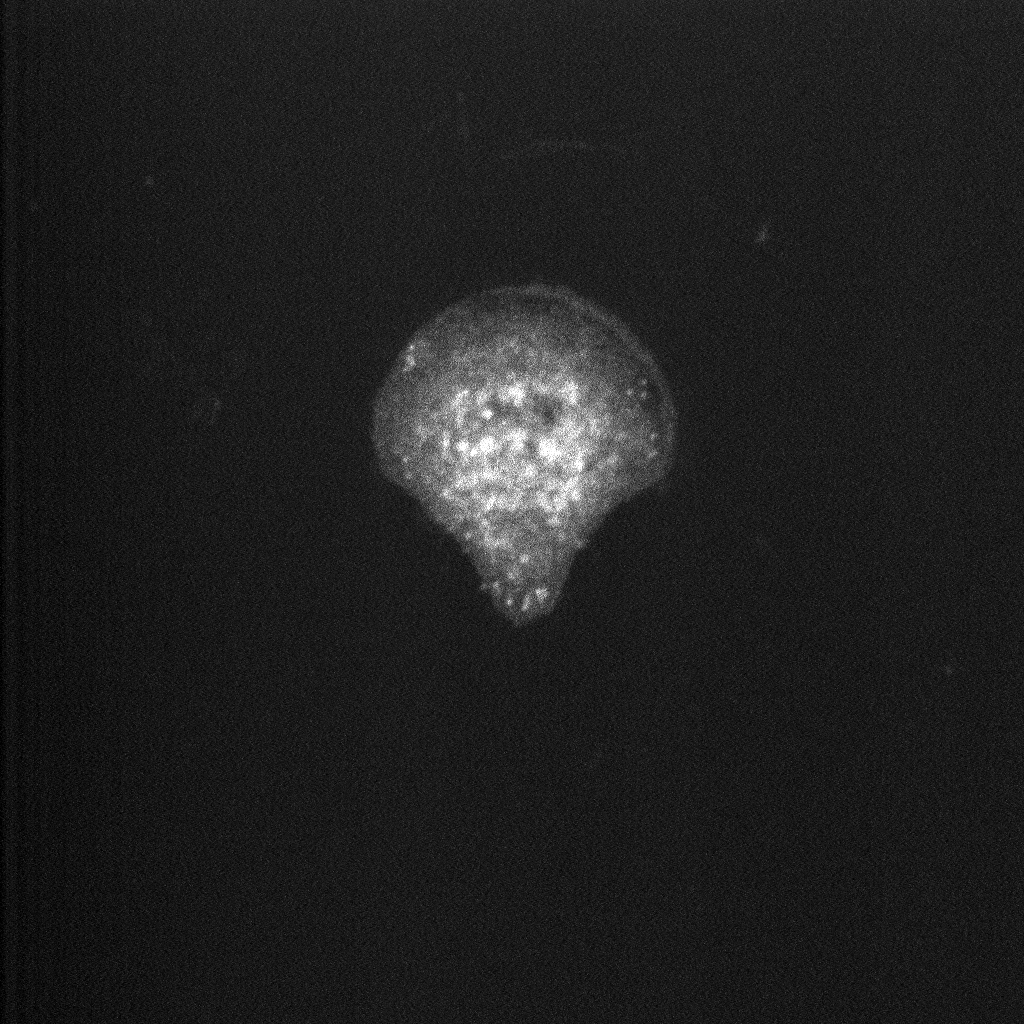

Supplement: Supplementary file 9 — Source Data for Figure 6 [file EMBJ-41-e110596-s003.zip › Data/Fig6E_Small.tif]

Source data file

Fig. 6D

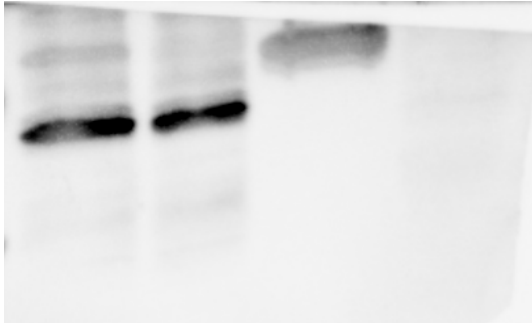

anti-Sar1  
Low  
exposure

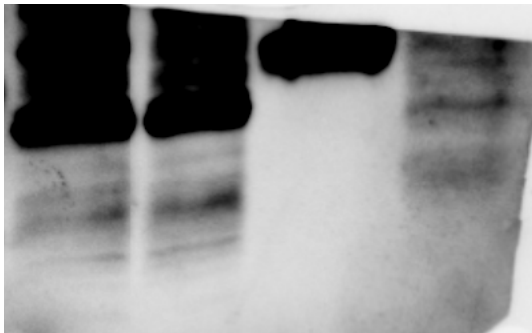

anti-Sar1  
High  
exposure

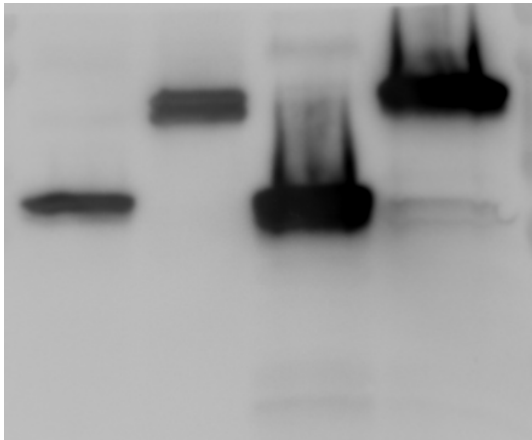

anti-  
GFP

Supplement: Supplementary file 9 — Source Data for Figure 6 [file EMBJ-41-e110596-s003.zip › SD_Fig_6.pdf]

**Fig. 7E**

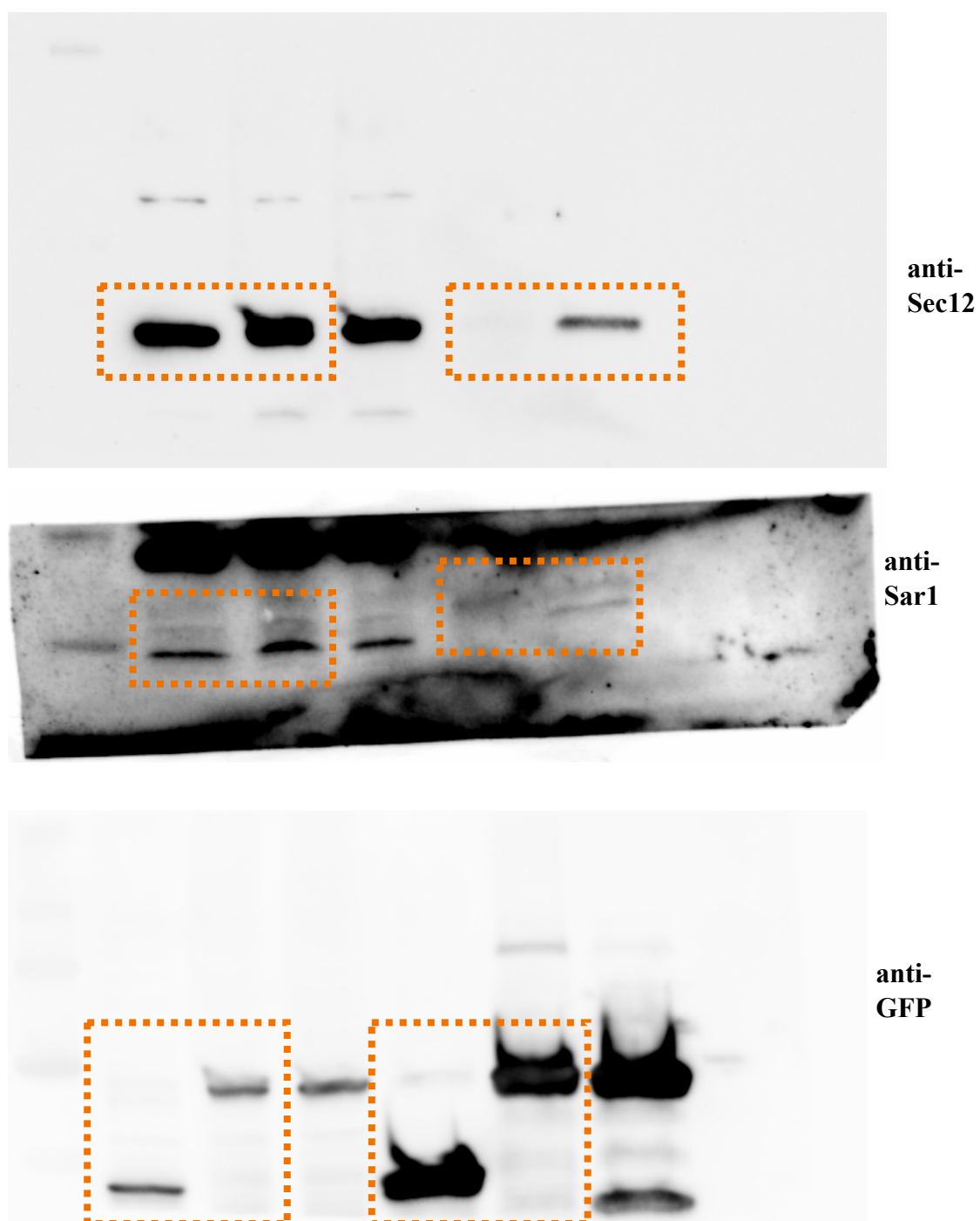

**Fig. 7F**

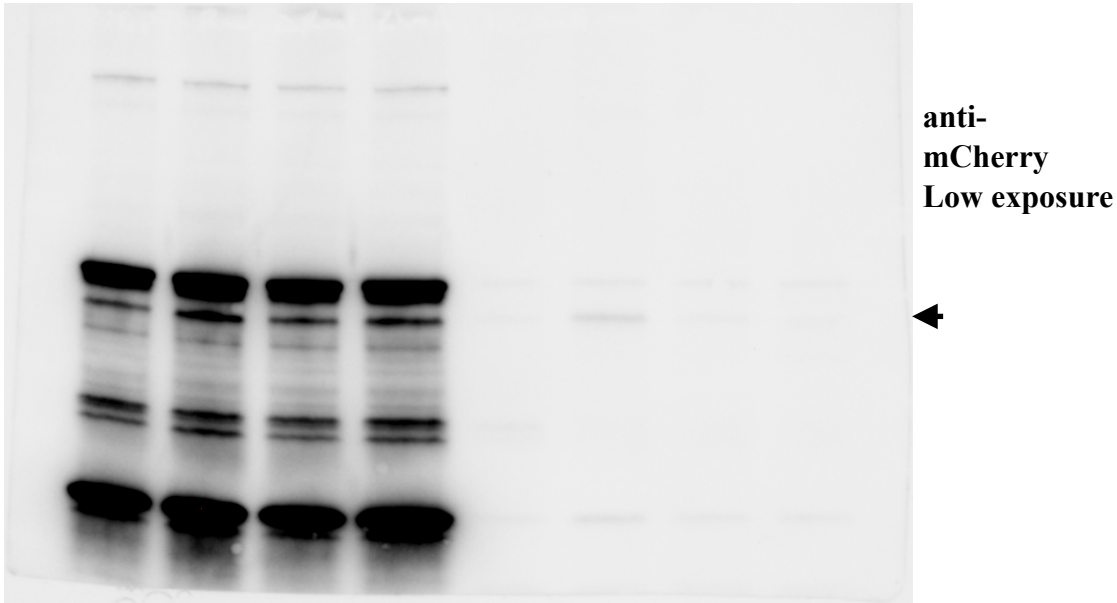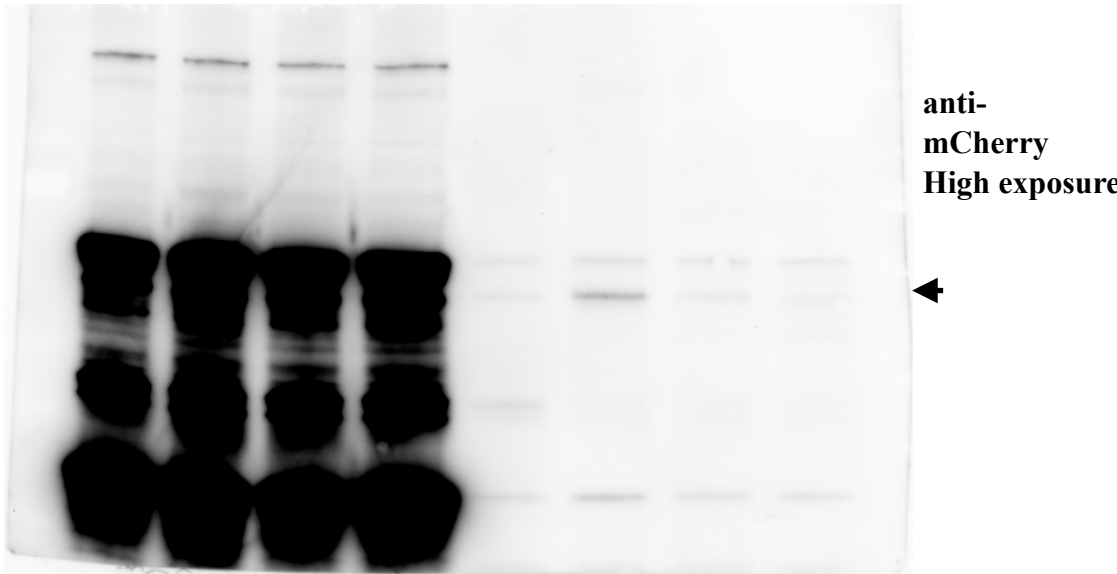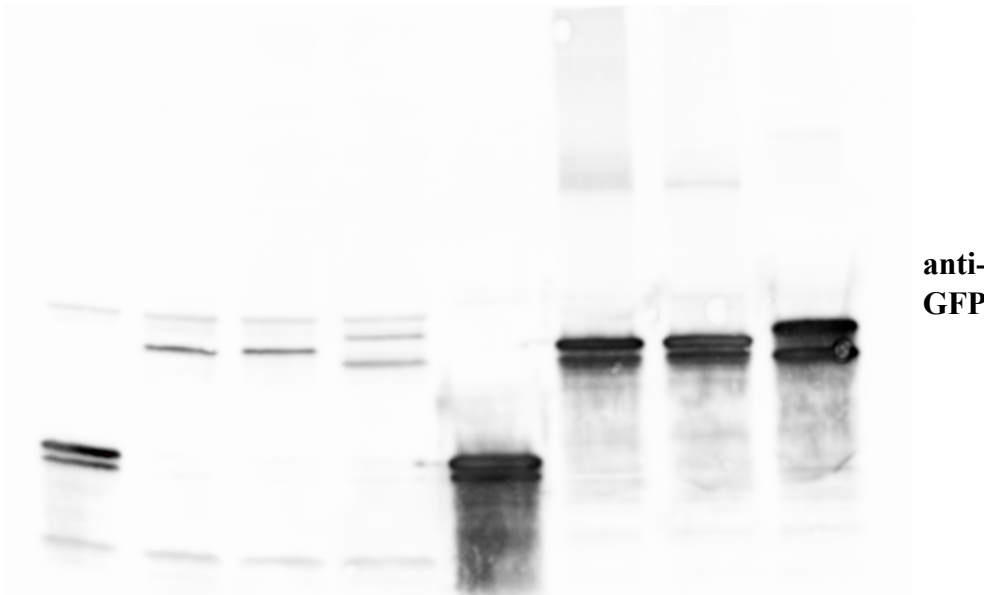

Supplement: Supplementary file 10 — Source Data for Figure 7 [file EMBJ-41-e110596-s011.pdf]

**Fig. 8A**

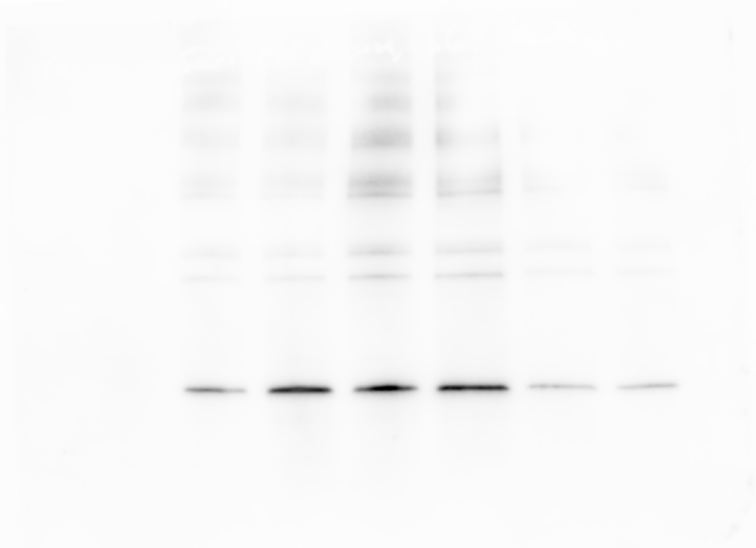

**anti-Sar1**

**Fig. 8B**

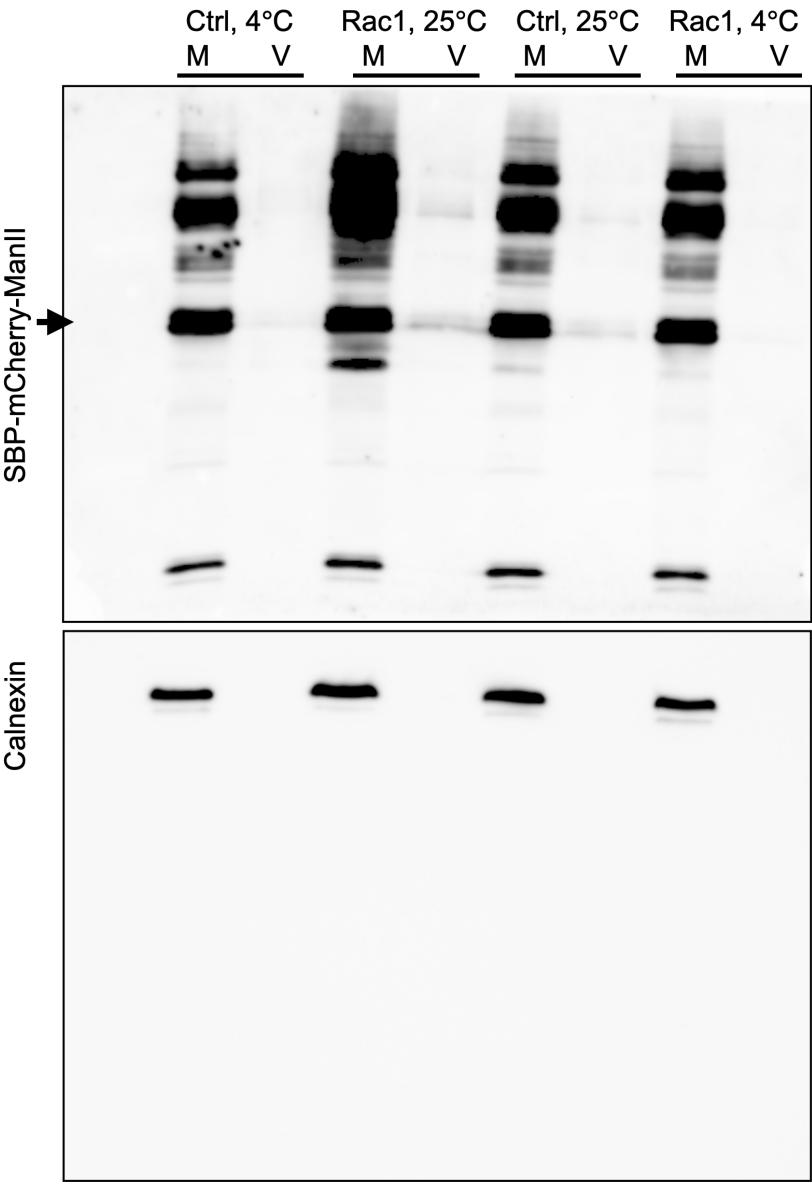

Supplement: Supplementary file 11 — Source Data for Figure 8 [file EMBJ-41-e110596-s002.pdf]
