# Supplementary material for: Mechanical strain stimulates COPII‐dependent secretory trafficking via Rac1
Source: EMBO J. 2022 Aug 8;41(18):e110596. doi: 10.15252/embj.2022110596 (PMC9475550; doi:10.15252/embj.2022110596)
Supplement: Supplementary file 12 [file EMBJ-41-e110596-s009.pdf]

THE EMBO JOURNAL

Mechanical strain stimulates COPII-dependent secretory trafficking via Rac1

Santosh Phuyal, Elena Djaerff, Anabel-Lise Le Roux, Martin Baker, Daniela Fankhauser, Sayyed Mahdizadeh, Veronika Reiterer, Amirabbas Parizadeh, Edward Felder, Jennifer Kahlhofer, David Teis, Marcelo Kazanietz, Stephan Geley, Leif Eriksson, Pere Roca-Cusachs, and Hesso Farhan
DOI: 10.15252/embj.2022110596

Corresponding author(s): Hesso Farhan (hesso.farhan@medisin.uio.no) , Pere Roca-Cusachs (proca@ibecbarcelona.eu), Santosh Phuyal (santosh.phuyal@medisin.uio.no)

Review Timeline:

Submission Date:	4th Jan 22
Editorial Decision:	31st Jan 22
Revision Received:	23rd May 22
Editorial Decision:	15th Jun 22
Revision Received:	24th Jun 22
Editorial Decision:	24th Jun 22
Revision Received:	1st Jul 22
Accepted:	5th Jul 22

Editor: William Teale

Transaction Report:

(Note: With the exception of the correction of typographical or spelling errors that could be a source of ambiguity, letters and reports are not edited. Depending on transfer agreements, referee reports obtained elsewhere may or may not be included in this compilation. Referee reports are anonymous unless the Referee chooses to sign their reports.)

1st Editorial Decision **31st Jan 2022**

Dear Dr. Farhan,

Thank you again for the submission of your manuscript entitled "Mechanical strain stimulates COPII-dependent trafficking via Rac1". We have now received the reports from three referees, which I have copied to the bottom of this message. As you can see from their comments, all of the referees appreciate the significance of your central finding and two are generally supportive of publication. Therefore, based on this overall interest, I would like to invite you to address the comments of all referees in a revised version of the manuscript. As you will see, certain points are consistently raised across all three reports, meaning some aspects of the study will require further experimental support before we can progress towards publication in The EMBO Journal. After a cross-consultation session among the reviewers, three specific issues have emerged. These are:

Firstly, all reviewers raise important technical concerns about the microscopy workflows used in the study. Here, I can see two particularly important comments: i) the issue of the variation among ratios between fluorescence signals in the RUSH experiments (as raised by reviewer 1 - point 2), and ii) that experimental artifacts may mean that Rac1 inhibition may not actually be causing a reduction ERES number, or that the effect may be cell-line-specific (as raised by reviewer 2 - paragraphs 3-7). Please also reconsider the suitability of all statistical tests that you have used throughout the manuscript.
Secondly, you should address the question of the persuasiveness of your IP data. All reviewers were concerned about the robustness of your negative controls. Please consider using a control protein which is closer to Rac1 (such as GFP-RhoA), in addition to the constitutively active and dominant negative Rac1 variants.
Thirdly, it may be possible, as you address these concerns, to use the tools you have already developed to gain a deeper general understanding of the relationship between Rac1 and ERES (as pointed out by reviewer 3 - points 3 and 4).

I think it would be a good idea to arrange a Zoom call together at your convenience to allow us to discuss these points and make sure no misunderstandings happen. Please indicate when you might be able to make yourself available.

When preparing your letter of response to the referees' comments, please bear in mind that this will form part of the Review Process File, and will therefore be available online to the community. For more details on our Transparent Editorial Process, please visit our website: https://www.embopress.org/page/journal/14602075/authorguide#transparentprocess

We generally allow three months as standard revision time. As a matter of policy, competing manuscripts published during this period will not negatively impact on our assessment of the conceptual advance presented by your study. However, we request that you contact the editor as soon as possible upon publication of any related work, to discuss how to proceed. Should you foresee a problem in meeting this three-month deadline, please let us know in advance and we may be able to grant an extension.

Thank you for the opportunity to consider your work for publication. I look forward to your revision.

Yours sincerely,

William Teale

William Teale, PhD
Editor
The EMBO Journal
w.teale@embojournal.org

When submitting your revised manuscript, please carefully review the instructions below and include the following items:

1) a .docx formatted version of the manuscript text (including legends for main figures, EV figures and tables). Please make sure that the changes are highlighted to be clearly visible.

2) individual production quality figure files as .eps, .tif, .jpg (one file per figure).

3) a .docx formatted letter INCLUDING the reviewers' reports and your detailed point-by-point response to their comments. As part of the EMBO Press transparent editorial process, the point-by-point response is part of the Review Process File (RPF), which will be published alongside your paper.

4) a complete author checklist, which you can download from our author guidelines (https://wol-prod-cdn.literatumonline.com/pb-assets/embo-site/Author Checklist%20-%20EMBO%20J-1561436015657.xlsx). Please insert information in the checklist that is also reflected in the manuscript. The completed author checklist will also be part of the RPF.

5) Please note that all corresponding authors are required to supply an ORCID ID for their name upon submission of a revised

manuscript.

6) We require a 'Data Availability' section after the Materials and Methods. Before submitting your revision, primary datasets produced in this study need to be deposited in an appropriate public database, and the accession numbers and database listed under 'Data Availability'. Please remember to provide a reviewer password if the datasets are not yet public (see https://www.embopress.org/page/journal/14602075/authorguide#datadeposition). If no data deposition in external databases is needed for this paper, please then state in this section: This study includes no data deposited in external repositories. Note that the Data Availability Section is restricted to new primary data that are part of this study.

Note - All links should resolve to a page where the data can be accessed.

7) When assembling figures, please refer to our figure preparation guideline in order to ensure proper formatting and readability in print as well as on screen:
http://bit.ly/EMBOPressFigurePreparationGuideline

Please remember: Digital image enhancement is acceptable practice, as long as it accurately represents the original data and conforms to community standards. If a figure has been subjected to significant electronic manipulation, this must be noted in the figure legend or in the 'Materials and Methods' section. The editors reserve the right to request original versions of figures and the original images that were used to assemble the figure.

8) For data quantification: please specify the name of the statistical test used to generate error bars and P values, the number (n) of independent experiments (specify technical or biological replicates) underlying each data point and the test used to calculate p-values in each figure legend. The figure legends should contain a basic description of n, P and the test applied. Graphs must include a description of the bars and the error bars (s.d., s.e.m.).

9) We would also encourage you to include the source data for figure panels that show essential data. Numerical data can be provided as individual .xls or .csv files (including a tab describing the data). For 'blots' or microscopy, uncropped images should be submitted (using a zip archive or a single pdf per main figure if multiple images need to be supplied for one panel). Additional information on source data and instruction on how to label the files are available at .

10) We replaced Supplementary Information with Expanded View (EV) Figures and Tables that are collapsible/expandable online (see examples in https://www.embopress.org/doi/10.15252/embj.201695874). A maximum of 5 EV Figures can be typeset. EV Figures should be cited as 'Figure EV1, Figure EV2" etc. in the text and their respective legends should be included in the main text after the legends of regular figures.

- For the figures that you do NOT wish to display as Expanded View figures, they should be bundled together with their legends in a single PDF file called *Appendix*, which should start with a short Table of Content. Appendix figures should be referred to in the main text as: "Appendix Figure S1, Appendix Figure S2" etc. See detailed instructions regarding expanded view here: .

- Additional Tables/Datasets should be labelled and referred to as Table EV1, Dataset EV1, etc. Legends have to be provided in a separate tab in case of .xls files. Alternatively, the legend can be supplied as a separate text file (README) and zipped together with the Table/Dataset file.

11) Our journal encourages inclusion of *data citations in the reference list* to directly cite datasets that were re-used and obtained from public databases. Data citations in the article text are distinct from normal bibliographical citations and should directly link to the database records from which the data can be accessed. In the main text, data citations are formatted as follows: "Data ref: Smith et al, 2001" or "Data ref: NCBI Sequence Read Archive PRJNA342805, 2017". In the Reference list, data citations must be labeled with "[DATASET]". A data reference must provide the database name, accession number/identifiers and a resolvable link to the landing page from which the data can be accessed at the end of the reference. Further instructions are available at .

Instructions for preparing your revised manuscript:

Please make sure you upload a letter of response to the referees' comments together with the revised manuscript.

Please also check that the title and abstract of the manuscript are brief, yet explicit, even to non-specialists.

When assembling figures, please refer to our figure preparation guideline in order to ensure proper formatting and readability in print as well as on screen:
https://bit.ly/EMBOPressFigurePreparationGuideline
See also guidelines for figure legends: https://www.embopress.org/page/journal/14602075/authorguide#figureformat

IMPORTANT: When you send the revision we will require
- a point-by-point response to the referees' comments, with a detailed description of the changes made (as a word file).
- a word file of the manuscript text.
- individual production quality figure files (one file per figure)
- a complete author checklist, which you can download from our author guidelines (https://www.embopress.org/page/journal/14602075/authorguide).
- Expanded View files (replacing Supplementary Information)
Please see out instructions to authors
https://www.embopress.org/page/journal/14602075/authorguide#expandedview

Please remember: Digital image enhancement is acceptable practice, as long as it accurately represents the original data and conforms to community standards. If a figure has been subjected to significant electronic manipulation, this must be noted in the figure legend or in the 'Materials and Methods' section. The editors reserve the right to request original versions of figures and the original images that were used to assemble the figure.

Further information is available in our Guide For Authors: https://www.embopress.org/page/journal/14602075/authorguide

We realize that it is difficult to revise to a specific deadline. In the interest of protecting the conceptual advance provided by the work, we recommend a revision within 3 months (1st May 2022). Please discuss the revision progress ahead of this time with the editor if you require more time to complete the revisions. Use the link below to submit your revision:

https://emboj.msubmit.net/cgi-bin/main.plex

Referee #1:

In this manuscript, the authors have investigated whether mechanical signals can impact the early secretory pathway. They show that a mechanical strain obtained by growing cells on micropatterns of different sizes or by biaxial stretching on PDMS membrane increases the number of endoplasmic reticulum exit sites (ERES) and stimulates ER-export of a secretory marker (mannosidase II). They provide evidence that this effect is dependent of a minor pool of Rac1 interacting with Sar1, which is involved in the biogenesis of COPII vesicles.
Overall, this is an interesting study that reveals an unexpected role of Rac1. The authors used a wide range of methodological approaches that support such a role. I have a few comments that should be addressed prior to publication:

1) Fig. 1E. A clear increase in FRET signal of the Rac1 biosensor is observed after stretching. I am not sure what this experience means given that only a minor pool of Rac1 is associated with ERES.
2) Fig. 1H (movie S2), Fig. 3G-H and Fig. 5G (RUSH experiments): the Golgi must be visualized by a marker for an accurate quantification of Man II arrival in it. In Figs. 3G and 5G, giantin was used. Is the Golgi was visualized with the same marker in Fig. 1H? The ratio between the fluorescence signal detected in Golgi and outside the Golgi is very different when comparing Figs. 3G and 5G with Fig. 1H. Is there a reason for this? Overall, the experiments shown in Fig. 1H are not convincing.
3) Fig. 2C: it is shown that the depletion of Sec16A does not affect cell size. What about Sar1 depletion?
4) Fig. 4G: The idea to activate Rac1 at the ER by optogenetics is nice but unfortunately the IF pictures provided are not very informative. Other pictures with an ER marker must be shown.

Referee #2:

This manuscript reports control of secretory pathway by mechanical cues through a direct interaction of Rac1 and Sar1. This would represent a novel and exciting development in the field. There are many external and intrinsic cues that impact on secretory activity and while perhaps not surprising that mechanical strain plays a role, the mechanism here would provide a rally compelling and exciting advance. Sar1 controls the assembly of the COPII complex at the ER, Rac1 controls multiple levels of actin cytoskeleton remodeling at various points through the cell.

The key to the paper is whether indeed Sar1 and Rac1 do indeed interact and whether mechanical strain does indeed impact COPII function.

The data in Figure 1 seek to define an increase in ERES number caused by cell spreading. While this could be the case, it is equally possible that the pre-existing ERES are less well clustered and therefore more definable using the software. This has

been shown previously in work on TFG for example. Indeed, that this change occurs within a small number of hours is challenging to reconcile with enhanced expression of COPII which would presumably be needed to account for this increase in ERES number (as happens for example in plasma cell differentiation).

I think the work suffers from being done in HeLa cells here in which COPII seems very tightly clustered and difficult to resolve in terms of individual puncta. While other cell types have been used to validate outcomes later in the paper, it would be better to see well spread cells here. Also one is left to question the generality given that these are a cancer cell line - is there a relevance to hard versus soft tumors here?

I struggled to interpret some of Figure 2 and question the use of statistics here in 2B (my interpretation is that in all cases depletion of Sar1 still reduces cell spread irrespective of micropattern. I think it difficult to conclude that these data support that "the early secretory pathway is required for adaptation to mechanical strain" rather that it suggests the early secretory pathway is required for cell spreading (which is largely considered known from its central role in both matrix biogenesis, integrin trafficking, and general membrane dynamics). Sec16A is used as a key COPII protein here (2C) yet I saw no validation of Sec16A knockdown or indication of how the degree of knockdown is correlated with cell-by-cell outcomes. Incomplete depletion could explain these data.

The Rac inhibition work is questionable from the conclusions drawn, I am not convinced for example that the inhibition f Rac1 activity does indeed reduces ERES number. Also, I was surprised by the apparent reproducibility of some data - e.g. what is the consistent peak of activity at 20 mins in 3E pink line? If these are true averages from independent replicates one would not expect that. I was also not clear if the data in 3G were from stable cell lines - expression levels of such trafficking reporters can have a big impact on trafficking efficiency. Also, for robustness, such data should really include a restoration of function such as RNAi resistant Rac1.

Also in terms of image processing - in "background subtraction" is the same parameter applied to all images in a data set (as it must be) or on a case-by case/per image "auto calculated" basis? If the latter, this will in my view invalidate the data. If uniformly applied how is the background calculated i.e. on which image?

I did not find the colocalization data in Figure 4 convincing. Rac1 might be on endosomes at ER-endosome contact sites or at points of actin-ER crossover. Even if one accepts "colocalization" 2 spots with partial overlap seems to low to explain significant impacts on cell function. How does this change under strain? The images also look overly processed - raw data should be made available.

What validation has been done of GFP-Rac1 as a reporter?

In 4G and H again I would like to have been able to review the underpinning data. These changes seem small and no statistical test is applied. No indication is given of reproducibility either.

The KDELR experiments are intriguing but incomplete - surely these need to be linked to assays of Rac activation? Also KDELR2 will cycle from the ER to the Golgi so will not "only act at the ER".

My major concern lies with the interaction data - the modelling is nice but needs experimental support. That shown does not robustly provide this. In Figure 7 I am not convinced by the IP data owing to a lack of controls. Negative controls of other small GTP binding proteins, other COPII proteins etc must be included. Similarly in Fig 8 the gels are overcropped - full gels/blots must be shown and negative and indeed positive controls are essential here. The data hint at the possibility of an interaction but are not sufficient to support the strength of conclusions drawn. Fig 8 is similarly unconvincing, not quantified, and lacks controls (e.g. Rho as a negative control, dominant negative and constitutively active Rac mutants, use of the Rac inhibitor).

Concerns on the statistical data - in every case, the numbers of technical and biological replicates must be stated. In many cases the data are not compelling and "hidden" in histograms when the original data should be included and plots done as scatter/violin/etc. SEM is not appropriate here either - standard deviations should be shown in all cases (see https://rupress.org/jcb/article/177/1/7/34602/Error-bars-in-experimental-biology)

The t-test is likely not appropriate at all as most of the data do not look normally distributed. A non-parametric test with multiple comparisons is likely more appropriate.

I am sorry that I cannot be more positive about this work. The topic is very exciting and has significant potential for in vivo relevance. That said, the data do (at least not yet) sufficiently support the conclusions.

Referee #3:

This manuscript from the Farhan group addresses an important question in the secretion field. How cells regulate secretion at

the level of ER exit sites (ERES) and other cellular structures remains poorly understood. In fact, even specific conditions that trigger regulation of secretion are not fully catalogued/documented. Mechanical stimulation is known to influence the plasma membrane (and endocytosis), cell growth and motility, all of which depend to some extent on secretion. Here, the authors probe the link between mechanical strain and secretion. The topic is exciting, and the findings are important. The main finding is that ERES abundance does indeed respond to mechanical stress, and that this is dependent on Rac1. The authors go on to show that Rac1 may play a more constitute role in ERES maintenance, and here there is a lost opportunity that would leverage the mechanical strain approaches developed in the first half of the study to reinforce the role for Rac1.

I have several specific criticisms:

1. On p. 3 the authors claim that the observed increase in ERES is not due to increase in volume/biomass. Although I appreciate the short time frame of the experiment, I'm not sure I understand the broader reasoning here. To substantiate this claim, the authors could measure cell volume as well as surface area and ERES number to demonstrate the relationships between these parameters more precisely.
2. Fig. 1E: the FRET sensor would be an excellent reporter to use in large vs. small cells to further substantiate the Rac1 effects.
3. The effect of Sar1 knockdown on cell spreading is profound. I was left wondering why Rac1 inhibition does not similarly cause cells to fully spread, especially if Rac1 activation lies upstream of Sar1 and its role in ERES.
4. The authors go on to show that Rac1 may play a more constitutive role in ERES maintenance/function. Here I felt there is room to leverage the tools to better probe the mechanical effects. For example, does KDELR-GAP overexpression abrogate stretching effects? Similarly, does CytoD/LatA treatment abrogate the stretch response, implying broader stretch effects beyond local Rac1? Finally, do Rac1/Sar1 BiFC puncta increase upon mechanical strain?
5. Controls for the Rac1-Sar1 BiFC experiment are missing (ie no fluorescence when each partner is expressed alone).
6. In Figure 7, the pulldowns showing Rac1/Sar1 interaction, and the "active Sar1" assay should include additional GDP and/or GMP-PNP controls to demonstrate nucleotide specificity.

Minor items:
• Figure 4D should show larger magnifications of ERES as insets, similar to Fig 4A (other puncta are also visible here, is the identity of those spots known?).
• Figure 4H: image data should be shown at least as a supplement.
• Figure 8: quantification of 2 experiments without error bars is not great. I suggest to just show quantifications of both individual experiments if a third repeat is not feasible.

Response to the Editorial comments:
Firstly, all reviewers raise important technical concerns about the microscopy workflows used in the study. Here, I can see two particularly important comments: i) the issue of the variation among ratios between fluorescence signals in the RUSH experiments (as raised by reviewer 1 - point 2),
Response: We think that there might be a misunderstanding. There is no big variation. In Figure 3G the ratio after 20 min of biotin addition is around 8. In Figure 5G the ratio measured at 20 min after biotin is has a value of 6.5. The end points of both figures are different (20 min in one and 30 min in the other). This might have caused the confusion.

ii) that experimental artifacts may mean that Rac1 inhibition may not actually be causing a reduction ERES number, or that the effect may be cell-line-specific (as raised by reviewer 2 - paragraphs 3-7).
Response: As far as cell line specificity is concerned, we showed already in the previous version that mechanical stimuli and/or Rac1 inhibition affects the number of ERES in HeLa, PC3 and MDA-MB-231 cells. Because all of these cell lines are transformed, we have now used RPE-1 cells (**Fig.1C, Fig.S1C-E**) and find that ERES in these cells also respond to mechanical stimuli in Rac1-dependent manner. Thus, our observations are not cell line specific.
As for the other point raised by reviewer 2, which concerns whether the increase of ERES upon stretching is real, or whether it is simply a redistribution of ERES. We think the increase is genuine for two reasons: firstly, if the increase were not a real one, but only due to redistribution of ERES, then it would be expected to occur in any condition, irrespective of the biological context. However, we observe the increase only in control cells and not in Rac1 inhibited cells. Secondly, the increase of ERES is in line with the acceleration of ER-to-Golgi trafficking as observed in the RUSH assay. A mere redistribution will not cause a change in ER-to-Golgi trafficking. Therefore, we think that our conclusions and interpretations of these data is valid.

Please also reconsider the suitability of all statistical tests that you have used throughout the manuscript.
Response: We have now elaborated “Statistical analyses” section in the methods. We have also updated all figure legends to better describe the statistical tests that were used, the number of experiments and the number of cells quantified from each experiment.

Secondly, you should address the question of the persuasiveness of your IP data. All reviewers were concerned about the robustness of your negative controls. Please consider using a control protein which is closer to Rac1 (such as GFP-RhoA), in addition to the constitutively active and dominant negative Rac1 variants.
Response: We have performed the coIP with GFP-RhoA as suggested, and did not detect any interaction. These data have now been added to the manuscript (**Fig.7F**).
To further support the Rac1-Sar1 interaction data, we used the computational modeling to predict residues in Rac1 that mediate an interaction with Sar1. We mutated these residues and observed a marked reduction of the interaction (**Fig. 7F**). Thus, our IP data have now been substantially strengthened.
We have refrained from using constitutively active and dominant negative Rac1 variants for reasons specified in response to reviewer #2 (please check the response at the end of p.6-7). Rather, we chose to use GTPγS and GDP as suggested by Reviewer 3.

Thirdly, it may be possible, as you address these concerns, to use the tools you have

already developed to gain a deeper general understanding of the relationship between Rac1 and ERES (as pointed out by reviewer 3 - points 3 and 4).
Response: We have addressed both points raised by the reviewer. In point 3, the reviewer asked us to test whether Rac1 inhibition has an impact on cell spreading on large micropatterns. This was indeed the case, and these data are now part of the revised manuscript (**Fig.2D&E**).
In point 4, the reviewer asked for a couple of experiments. On one hand, the reviewer asked us to test whether inhibition of local Rac1 activity at the ER abrogates stretching effects. We tested this by culturing cells that express the ER-anchored Rac1-GAP on Crossbow micropattern, and noted a reduction in ERES only when cells were forced to grow larger (**Fig.4G&H, Fig. S4F&G**).
The reviewer also asked us to test whether actin is involved in mediating the stretch response of ERES under mechanical stimulation. This is a relevant control experiment because we ruled out that actin plays a role in mediating the effect of Rac1 on ERES under standard growth conditions. We have performed the experiment the reviewer asked for and data are presented in **Fig.5F&G**. We found that actin disruption with cytochalasin D during mechanical strain did not prevent the increase in ERES number. These new data further support the notion that actin does not mediate mechanotransduction signaling to ERES (at least not in the context of our experiments).

Response to Referee #1:

In this manuscript, the authors have investigated whether mechanical signals can impact the early secretory pathway. They show that a mechanical strain obtained by growing cells on micropatterns of different sizes or by biaxial stretching on PDMS membrane increases the number of endoplasmic reticulum exit sites (ERES) and stimulates ER-export of a secretory marker (mannosidase II). They provide evidence that this effect is dependent of a minor pool of Rac1 interacting with Sar1, which is involved in the biogenesis of COPII vesicles. Overall, this is an interesting study that reveals an unexpected role of Rac1. The authors used a wide range of methodological approaches that support such a role. I have a few comments that should be addressed prior to publication:
Response: We thank the reviewer for their positive comments and constructive criticism.

1) Fig. 1E. A clear increase in FRET signal of the Rac1 biosensor is observed after stretching. I am not sure what this experience means given that only a minor pool of Rac1 is associated with ERES.
Response: We agree that the mechanically activated global Rac1 pool has other effects in cells in addition to signaling to ERES. Thus, we think that only a part of the mechanically activated Rac1 pool signals to ERES. To get an estimation of how big this pool might be, we tested the impact of the ER-anchored Rac1-GAP on total Rac1 activity. We observed that the reduction is in the range of 10-15% as shown in the new **Fig.S4H**. Of course, this is even an overestimation, because an ER-anchored Rac1-GAP might also affect Rac1 pools that are in close vicinity to the ER.

2) Fig. 1H (movie S2), Fig. 3G-H and Fig. 5G (RUSH experiments): the Golgi must be visualized by a marker for an accurate quantification of Man II arrival in it. In Figs. 3G and 5G, giantin was used. Is the Golgi was visualized with the same marker in Fig. 1H? The ratio between the fluorescence signal detected in Golgi and outside the Golgi is very different

when comparing Figs. 3G and 5G with Fig. 1H. Is there a reason for this? Overall, the experiments shown in Fig. 1H are not convincing.
Response: The ratio is approximately the same in 3G-H and 5G (now 5K). In Fig. 3G-H the ratio after 20 min is around 8. In Fig. 5G the ratio is measured at 20 min after biotin and has an average value of 6.5. This is generally within the normal variation. The ratio in Fig.1H (now Fig. 1J) is different than the ones in Fig. 3H and 5K because this experiment was done in a different way than the ones in Fig.3H and 5K. Firstly, the experiment in Figure 1H is a live cell imaging experiment where cells were grown on the PDMS membranes, while the ones in Figs. 3&5 are fixed cells grown on glass coverslips. Another difference is that the experiment in Figure 1H was imaged with an epifluorescence microscope equipped with a mercury lamp, while the experiments in Figs. 3&5 were imaged at a laser scanning confocal/spinning-disk microscopes. We are aware of this difference, but the overall trend is the same in both experiments.
We think that the experiment in Fig.1H is a nice contribution to our manuscript. We understand the reviewer's concern because no Golgi marker was used. However, we used Mannosidase-II as a RUSH reporter, which is a Golgi protein. Hence, ManII will only traffic to the Golgi, and at the end of the RUSH experiment serve as an indicator for Golgi position. This can be appreciated from Fig. 3G, Fig.5C and J (timepoint 30 min). Furthermore, we did not use a RUSH reporter that traffics beyond the Golgi (such as VSVG), because in such a case the signal in the Golgi peaks (as the cargo enters the Golgi) and then drops (as the cargo leaves the Golgi). In the case of ManII, the signal increases in the Golgi region and then reaches a plateau. This makes assessing the arrival of Golgi residing cargos in RUSH experiments possible even in the absence of the Golgi markers. We have also modified Fig.1H (now Fig. 1J) to improve the data visualization for Fig.1H (now Fig. 1J). Finally, the inherent nature of PDMS membrane make live cell imaging (focusing issues) over time a challenging task, which is why an epifluorescence microscope was used and therefore colocalizations would anyway not have been possible. We very much hope to have convinced the reviewer to keep the data in Figure 1H as part of our manuscript.

3) Fig. 2C: it is shown that the depletion of Sec16A does not affect cell size. What about Sar1 depletion?
Response: We performed the experiment asked by the reviewer and the new data is now shown in **Fig. 2C**, where we show that Sar1A/B depletion has no effect on cell size. The Sec16A knockdown and cell size data is now part of the supplementary **Fig.S2G&H**.

4) Fig. 4G: The idea to activate Rac1 at the ER by optogenetics is nice but unfortunately the IF pictures provided are not very informative. Other pictures with an ER marker must be shown.
Response: We used an ER-residing protein Sec61β to recruit TIAM to the ER. As recommended by the reviewer, we have now included Sec61β in the figure and show localization of both TIAM and Sec61β over time following the optogenetic recruitment. This is now shown in **Fig.4I**.

Response to Referee #2:

This manuscript reports control of secretory pathway by mechanical cues through a direct interaction of Rac1 and Sar1. This would represent a novel and exciting development in the field. There are many external and intrinsic cues that impact on secretory activity and while perhaps not surprising that mechanical strain plays a role, the mechanism here would

provide a rally compelling and exciting advance. Sar1 controls the assembly of the COPII complex at the ER, Rac1 controls multiple levels of actin cytoskeleton remodeling at various points through the cell.

The key to the paper is whether indeed Sar1 and Rac1 do indeed interact and whether mechanical strain does indeed impact COPII function.
Response: We thank the reviewer for their critical assessment of our manuscript and suggestions for improvement.

The data in Figure 1 seek to define an increase in ERES number caused by cell spreading. While this could be the case, it is equally possible that the pre-existing ERES are less well clustered and therefore more definable using the software. This has been shown previously in work on TFG for example. Indeed, that this change occurs within a small number of hours is challenging to reconcile with enhanced expression of COPII which would presumably be needed to account for this increase in ERES number (as happens for example in plasma cell differentiation).
Response: We agree with the reviewer that the ERES increase is unlikely due to *de novo* synthesis of COPII, because of the short time frame of our experiments (few minutes to maximally 4 h). We thank the reviewer for raising this important point, which we have now discussed. Then there is the possibility of redistribution of ERES rather than a genuine increase. We think the increase in ERES number is genuine for two reasons: firstly, if the increase were not a real one, but only due to redistribution of ERES, then it would be expected to occur in any condition, irrespective of the biological context. However, we observe the increase only in control cells and not in Rac inhibited cells. Secondly, the increase of ERES is in line with the acceleration of ER-to-Golgi trafficking as observed in the RUSH assay. A mere redistribution will not cause a change in ER-to-Golgi trafficking. Therefore, we think that our conclusions and interpretations of these data is valid.

I think the work suffers from being done in HeLa cells here in which COPII seems very tightly clustered and difficult to resolve in terms of individual puncta. While other cell types have been used to validate outcomes later in the paper, it would be better to see well spread cells here. Also one is left to question the generality given that these are a cancer cell line - is there a relevance to hard versus soft tumors here?
Response: In addition to HeLa cells, we had already used 2 other cell lines (PC3 and MDA-MB-231) to test the generality of our findings. In response to the reviewer's comment, we obtained new data from experiments carried out with mechanically challenged RPE-1 cells, which are non-transformed cells (**Fig.1C, Fig.S1C-E**), to further strengthen our results.
In our experience, HeLa cells are well spread cells and we observe the juxtanuclear clustering of ERES in all cell lines we ever worked on (HeLa, HeLaK, HeLaS3, HEK239, HEK293T, MDA-MB-231, BT549, U2OS, PC3, primary fibroblasts, various multiple myeloma cells, and RPE-1). Most importantly, we can segment and count the juxtanuclear ERES (please see the image with overlaid count masks below).
While an interesting topic, the investigations of how ERES adapt to soft and stiff substrates is beyond the scope of this work. Another highly relevant mechanical stress is compression, and we are planning to investigate this in the future. However, the current work focuses only on stretching as a mechanical stimulus.

I struggled to interpret some of Figure 2 and question the use of statistics here in 2B (my interpretation is that in all cases depletion of Sar1 still reduces cell spread irrespective of micropattern. I think it difficult to conclude that these data support that "the early secretory

pathway is required for adaptation to mechanical strain" rather that it suggests the early secretory pathway is required for cell spreading (which is largely considered known from its central role in both matrix biogenesis, integrin trafficking, and general membrane dynamics). Sec16A is used as a key COPII protein here (2C) yet I saw no validation of Sec16A knockdown or indication of how the degree of knockdown is correlated with cell-by-cell outcomes. Incomplete depletion could explain these data.

Response:

We agree with the reviewer that our experiment shows how ERES regulate the ability of cells to spread, and we have added this phrase now to the text. However, we also think that the spread defect on a large micropattern also reflects a reduced ability of cells to adapt to mechanical stress. Spreading of cells on micropatterns is different that the spontaneous cell spreading. The reviewer has correctly pointed out that spontaneous spreading is something that happens mainly as a combination of internal forces in cells (e.g. actin) together with integrin and matrix deposition. However, spreading of cells on a micropattern is different from this, because cells are “forced” to adopt a geometry of certain size. Work by others showed that cells experience more tension at their cell surface when asked to cover a larger geometry. We think that the secretory pathway helps cells cope with this stress by controlling membrane supply. We agree with the reviewer that the secretory pathway controls membrane flux, but we are unaware of any previous paper where the effect of COPII on cell spreading on micropatterns was tested.

To better convey this message and support this finding, we have added a new experiment. We reasoned that cells with reduced ERES function will exhibit more membrane damage when subjected to mechanical strain. To test this experimentally, we determined the membrane permeability of cells exposed to uniaxial stretch using propidium iodide. As shown in **Fig. S2F**, cells depleted of Sec16A become more permeable to propidium iodide 10-15 min after stretching. We hope that this clarification, as well as the new data increase the confidence of the reviewer in our data.

The reviewer also questions the choice of statistical test: We chose to perform Chi-square test because of the qualitative nature of the experiment (a binary variable in “yes” or “no” format resulting from the question: do cells fully occupy the micropatterns?). In such cases, the Chi-square test is a very good choice, and we are confident that it is suitable.

A blot validating our Sec16 siRNA is now shown in **Fig. S2G**. We thank the reviewer for correcting this oversight.

The Rac inhibition work is questionable from the conclusions drawn, I am not convinced for example that the inhibition f Rac1 activity does indeed reduces ERES number. Also, I was surprised by the apparent reproducibility of some data - e.g. what is the consistent peak of activity at 20 mins in 3E pink line? If these are true averages from independent replicates one would not expect that. I was also not clear if the data in 3G were from stable cell lines - expression levels of such trafficking reporters can have a big impact on trafficking efficiency. Also, for robustness, such data should really include a restoration of function such as RNAi resistant Rac1.

Also in terms of image processing - in "background subtraction" is the same parameter applied to all images in a data set (as it must be) or on a case-by case/per image "auto calculated" basis? If the latter, this will in my view invalidate the data. If uniformly applied how is the background calculated i.e. on which image?

Response: We have tested a role for Rac1 in regulating ERES in several ways (with and without mechanical stimulus), and provide strong evidence that Rac1 regulates ERES. We show that:

(i) Rac1 inhibition reduces ERES

(ii) Rac1 knockdown reduces ERES
(iii) Rac1 inhibition delays ER-to-Golgi trafficking
(iv) Rac1 knockdown delays ER-to-Golgi trafficking
(v) Rac1 inhibition does not affect ERES in Rac1-knockout cells (**Fig. S3I&J**), indicating an on-target effect
(vi) Rac1 interacts with Sar1
(vii) In the presence of Rac1, we observe more active Sar1

Therefore, we are confident that our conclusion that Rac1 regulates ERES function is correct and convincing.

As for the issue of counting ERES, our lab has a strong expertise in counting ERES, which is well documented in several of our previously published papers. Regardless, we include two sample images where we have overlaid count masks on ERES.

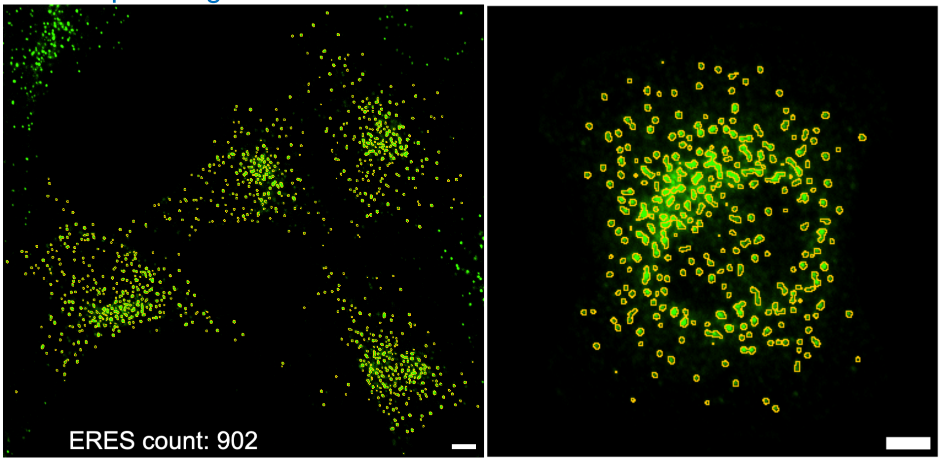

We assume that the reviewer is referring to the pink line in Fig. 3H since Fig.3E shows FRAP-curve of GFP-Sec16A and the time scale is in seconds. We apologize that the error bars were not properly displayed because of our formatting and choice to display standard error of mean. We have now modified Fig.3H from line graph (previously) to scatter plot where we show raw data distribution (76 – 104 cells in total from 3 independent experiments). The modified figure also shows mean and standard deviation.
We hope this addresses the concern.

All RUSH assays in this manuscript are performed with stable cell lines. We apologize that this information was not clear from the figure legends. We have now updated our figure legends with this information wherever necessary.
We had demonstrated in **Fig.S3C-E** that stable expression of siRNA resistant Rac1 renders the ERES in these cells resistant to siRNA. In addition, we have also performed NSC23766 washout experiments and show (**Fig.3C&D**) recovery of ERES in cells following the washout. We hope that this increases the confidence of the reviewer in our data.
In terms of image processing, the background subtraction was carried out in ImageJ, where a rolling ball radius of 50 pixels was applied to all images in a data set. All images within an experiment were processed and analyzed in exact same way. We have now specified this in the methods section of the manuscript.

I did not find the colocalization data in Figure 4 convincing. Rac1 might be on endosomes at ER-endosome contact sites or at points of actin-ER crossover. Even if one accepts "colocalization" 2 spots with partial overlap seems to low to explain significant impacts on cell function. How does this change under strain? The images also look overly processed - raw data should be made available.

Response: In response to reviewer's concern, we carried out (results shown in **Fig. S4C**) a triple colocalization study using HeLa cells that contain endogenously tagged Rac1. We immunostained GFP-Rac1 HeLa cells with an endosomal marker (EEA1) and ERES marker Sec31A. We did observe (as previously shown by others) Rac1 on endosomes. We found that Rac1 that colocalizes with the endosomal marker EEA1, does not colocalize with ERES. This suggests that the endosomal pool of Rac1 is distinct from ERES. These data are shown in Figure S4C.
The images shown in **Fig.4A** are SRRF images. We have not processed the image in anyway other than slightly adjust brightness and contrast. We had to perform SRRF imaging since the cytosolic background of Rac1 was masking the subtle pool of Rac1 at the ER. The imaging was performed on an Andor Dragonfly microscope that has the SRRF feature pre-installed (a feature that the company developed together with Ricardo Henriques, whose group described the SRRF imaging method). We constantly observed a small and a very rapid/transient pool of Rac1 at the ER. In response to the reviewer's question about the effect of mechanical stress on Rac1-Sar1 interaction, we performed BiFC experiment showing that cells on a large micropattern have more Rac1-Sar1 complexes than cells on a small micropattern.

What validation has been done of GFP-Rac1 as a reporter?
Response: We assume that the reviewer is referring to the cells with endogenously tagged Rac1. After endogenous tagging, we performed a series of control experiments to validate the newly generated cell line as shown in **Fig. S4A&B**. We designed PCR primers spanning different regions and observed PCR fragments as expected (**Fig. S4A left panel**). Importantly, Sanger sequencing result shows GFP-incorporation at the desired site (**Fig. S4A right panel)**. Finally, we also transfected these cells with siRNA against Rac1 and obtained a clear a reduction of the GFP-Rac1 levels (Fig. S4B). These data show that the cells contain endogenously tagged Rac1.

In 4G and H again I would like to have been able to review the underpinning data. These changes seem small and no statistical test is applied. No indication is given of reproducibility either.
Response: The quantification graph (previous 4G, now 4J) already shows raw data distribution. Perhaps this was not clear from the figure legend. An example image is shown now in Fig. S4I. We include counts/cell in the table below (this will also be made available as part of source data):

Table format: XY		X	Group A								
		Time (sec)	SNAP-Sec16A puncta/per cell								
		X	A:Y1	A:Y2	A:Y3	A:Y4	A:Y5	A:Y6	A:Y7	A:Y8	A:Y9
1	pre-recruitment	0	43	31	51	32	62	36	22	31	19
2	30 sec post	30	55	61	62	44	86	56	28	29	22
3	60 sec post	60	63	49	91	57	73	44	28	28	22
4	90 sec post	90	67	46	66	47	81	56	29	28	22
[illegible]	[illegible]										

When we run a paired t-test between t0 and t90, there is a statistical significant difference when comparing all cells measured. This has now been indicated in the quantification graph.
The KDELR experiments are intriguing but incomplete - surely these need to be linked to assays of Rac activation? Also KDELR2 will cycle from the ER to the Golgi so will not "only act at the ER".
Response: We agree that the construct is likely to cycle, but the main effect will be at the ER, because this is where the steady state localization of this construct is.

In response to the reviewer's comment, we performed a G-LISA assay and show that expression of the ER-anchored Rac1-GAP reduces the total cellular pool of active Rac1 by about 10-15%. This small, but reproducible effect, is in the range that we expected and show that we do not affect a bigger pool of Rac1 in cells.

My major concern lies with the interaction data - the modelling is nice but needs experimental support. That shown does not robustly provide this. In Figure 7 I am not convinced by the IP data owing to a lack of controls. Negative controls of other small GTP binding proteins, other COPII proteins etc must be included. Similarly in Fig 8 the gels are overcropped - full gels/blots must be shown and negative and indeed positive controls are essential here. The data hint at the possibility of an interaction but are not sufficient to support the strength of conclusions drawn.
Response: We agree with the reviewer, and have therefore performed more control experiments. As requested, we included RhoA as an additional control in our coIP experiments and show that RhoA does not interact with Sar1 (**Fig. 7F**). Of note, we used our *in silico* modeling strategy to predict key amino acids in Rac1 that is important for its interaction with Sar1. The two predicted amino acid residues were arginine at position 163 and lysine at position166. We mutated these residues in Rac1 and observed a reduced Rac1-Sar1 interaction (**Fig.7F**).
The uncropped blots are shown below as requested:

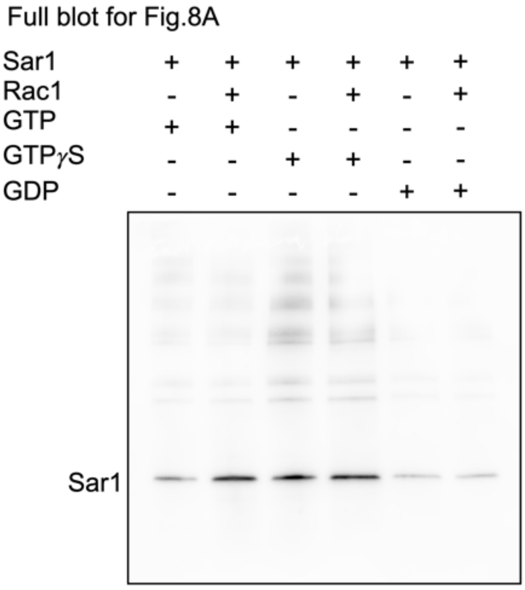

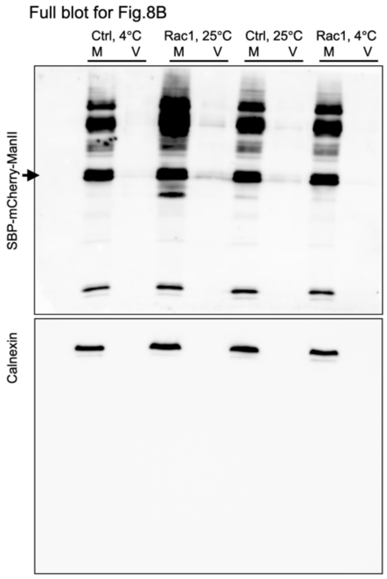

Fig 8 is similarly unconvincing, not quantified, and lacks controls (e.g. Rho as a negative control, dominant negative and constitutively active Rac mutants, use of the Rac inhibitor).
Response: A quantification of the budding assay is shown.
We think that using GTPγS and GDP is better than using dominant negative, or constitutively active mutants. Therefore, we repeated the active Sar1 pulldown experiments in the presence of GTPγS and GDP (**Fig. 8A**). GTPγS will allow for high levels of Sar1 activation and consequently, no effect of Rac1 is observed. Likewise, using GDP also abolished any effect of Rac1 on Sar1 activation.

Concerns on the statistical data - in every case, the numbers of technical and biological replicates must be stated. In many cases the data are not compelling and "hidden" in histograms when the original data should be included and plots done as scatter/violin/etc. SEM is not appropriate here either - standard deviations should be shown in all cases (see https://rupress.org/jcb/article/177/1/7/34602/Error-bars-in-experimental-biology)
The t-test is likely not appropriate at all as most of the data do not look normally distributed. A non-parametric test with multiple comparisons is likely more appropriate.
Response: We used SDs as suggested by the reviewer and changed the graphical presentation of the figures to include individual data points.

I am sorry that I cannot be more positive about this work. The topic is very exciting and has significant potential for in vivo relevance. That said, the data do (at least not yet) sufficiently support the conclusions.
Response: We hope that the new data and the amendments will convince the reviewer now that our work is a strong candidate.

Response to Referee #3:

This manuscript from the Farhan group addresses an important question in the secretion field. How cells regulate secretion at the level of ER exit sites (ERES) and other cellular structures remains poorly understood. In fact, even specific conditions that trigger regulation of secretion are not fully catalogued/documented. Mechanical stimulation is known to

influence the plasma membrane (and endocytosis), cell growth and motility, all of which depend to some extent on secretion. Here, the authors probe the link between mechanical strain and secretion. The topic is exciting, and the findings are important. The main finding is that ERES abundance does indeed respond to mechanical stress, and that this is dependent on Rac1. The authors go on to show that Rac1 may play a more constitute role in ERES maintenance, and here there is a lost opportunity that would leverage the mechanical strain approaches developed in the first half of the study to reinforce the role for Rac1.
Response: We thank the reviewer for recognizing the importance of our work and for providing constructive criticism.

1. On p. 3 the authors claim that the observed increase in ERES is not due to increase in volume/biomass. Although I appreciate the short time frame of the experiment, I'm not sure I understand the broader reasoning here. To substantiate this claim, the authors could measure cell volume as well as surface area and ERES number to demonstrate the relationships between these parameters more precisely.
Response: We agree with the reviewer that we might have speculated too much here without actually demonstrating this point with the increase in biomass. We have therefore removed this statement from the text. To accurately show this, we would have had to perform proteomic and lipidomic experiments to show that the biomass of our cells has not changed.

2. Fig. 1E: the FRET sensor would be an excellent reporter to use in large vs. small cells to further substantiate the Rac1 effects.
Response: We performed the experiment asked by the reviewer. As shown in **Fig.1F**, the FRET reporter indicates higher Rac1 activity in cells on a large micropattern than on a small micropattern corroborating the results obtained with mechanically stimulated cells grown on PDMS membranes.

3. The effect of Sar1 knockdown on cell spreading is profound. I was left wondering why Rac1 inhibition does not similarly cause cells to fully spread, especially if Rac1 activation lies upstream of Sar1 and its role in ERES.
Response: We performed this experiment and show that inhibition of Rac1 phenocopies the Sar1 with respect to cell spreading. The data are part of **Fig.2D&E.**

4. The authors go on to show that Rac1 may play a more constitutive role in ERES maintenance/function. Here I felt there is room to leverage the tools to better probe the mechanical effects. For example, does KDELR-GAP overexpression abrogate stretching effects? Similarly, does CytoD/LatA treatment abrogate the stretch response, implying broader stretch effects beyond local Rac1? Finally, do Rac1/Sar1 BiFC puncta increase upon mechanical strain?
Response: We overexpressed KDELR2-GAP and cultured these cells on micropatterns to quantitate ERES. We found that cells overexpressing KDELR2-GAP have less ERES when cultured on large micropattern (**Fig.4G&H**) similarly to the effect we observed with Rac1 inhibition shown in **Fig.1**.
We also performed the second experiment the reviewer asked for, where we show that CytoD treated cells still respond by increasing ERES number when subjected to stretch (**Fig. 5F&G**). This result supports the notion that actin is not involved in mediating the observed Rac1 effect on ERES.

Finally, we also performed the third experiment the reviewer asked for and show that cells on a large micropattern have more Rac1/Sar1 BiFC puncta than cells on a small micropattern. This is to be expected because cells on large micropatterns have more ERES. These data are shown in **Fig. 6E&F**.

5. Controls for the Rac1-Sar1 BiFC experiment are missing (ie no fluorescence when each partner is expressed alone).
Response: The requested controls are shown in **Fig. S6A**.

6. In Figure 7, the pulldowns showing Rac1/Sar1 interaction, and the "active Sar1" assay should include additional GDP and/or GMP-PNP controls to demonstrate nucleotide specificity.
Response: We repeated the IP experiments in the present of GTPγS and GDP (Figure 8A). GTPγS will allow for high levels of Sar1 activation and consequently, no effect of Rac1 is observed. Likewise, using GDP also abolished any effect of Rac1 on Sar1 activation.
To further increase the confidence in our IP experiments, we also tested whether RhoA interacts with Sar1, and it did not.
We also used modeling to predict two residues in Rac1 that could potentially mediate the interaction with Sar1. Mutation of these residues in Rac1 reduced the interaction with Sar1 as shown in Fig.7F.

Minor items:
• Figure 4D should show larger magnifications of ERES as insets, similar to Fig 4A (other puncta are also visible here, is the identity of those spots known?).
Response: We have included magnified insets as reviewer requested.

• Figure 4H: image data should be shown at least as a supplement.
Response: image data are shown now in the supplement (**Fig. S4I**)

• Figure 8: quantification of 2 experiments without error bars is not great. I suggest to just show quantifications of both individual experiments if a third repeat is not feasible.
Response: We followed the suggestion of the reviewer and display the data from two individual experiments.

Dear Hesso,

Thank you for submitting your manuscript for consideration by the EMBO Journal. It has now been seen for a second time by three referees whose comments are shown below.

As you will see you have addressed most of the referees' concerns to their satisfaction. However, referee 2 has some, I believe reasonable, residual concerns, especially over quantification and controls used in some of your Western blots. I therefore think it would be of general benefit to give you the opportunity to address these points. If you would like to have a quick Zoom call to go over the work, I will be available all next week.

Given the referees' positive recommendations, I would like to invite you to submit a revised version of the manuscript, addressing the comments of all three reviewers. I should add that it is EMBO Journal policy to allow only a single round of revision, and acceptance of your manuscript will therefore depend on the completeness of your responses in this revised version.

When preparing your letter of response to the referees' comments, please bear in mind that this will form part of the Review Process File, and will therefore be available online to the community. For more details on our Transparent Editorial Process, please visit our website: https://www.embopress.org/page/journal/14602075/authorguide#transparentprocess

Best wishes,

William

William Teale, PhD
Editor
The EMBO Journal
w.teale@embojournal.org

Referee #1:

The authors have addressed my previous comments in a satisfactory way.

Referee #2:

There is no doubt that the manuscript is much improved by the new data. However, I retain concerns on the colocalization data and interaction data. There remains an insufficient use of controls, notably in Figures 7 and 8. Blots e.g. 8B are saturated and not suitable for quantification and anyway ECL is only semi-quantitative. There are no controls in 8A - input? a small GTP binding protein that isn't impacts e.g. Rab1? Looking carefully at 7F Rac1 appears expressed at a higher level than the other proteins which could explain greater detection. This compromises the Rac1 KF/AA mutation data. The co-transfection approach here is not ideal - this should really be done with recombinant protein. In 8A these data require extensive repetition and quantification as surely addition of GTP or GTP-gamma-S will increase the population of "active" Sar1 in each experiment. I appreciate that the Rac1 lane acts as an internal control here but this is not in itself sufficient.

In terms of colocalization if one were to rotate the Sec61 channel, I am reasonably convinced that some "colocalization" would still be evident. Even Spot 2 indicates spots adjacent not overlapping. In other places the statistical testing does not support the conclusions e.g. 3D where the NSC washout has no effect compared to the adjacent column.

I simply don't think that the effect sizes support the strength of conclusions, particularly the title. Many of my original concerns remain.

Fundamentally, I do agree that there is something interesting going on here but I am not yet convinced that the data support a direct role for Rac1 in any ERES response to mechanical strain as claimed in the title.

Referee #3:

The authors have satisfactorily addressed all of my concerns and I consider the revised manuscript fully acceptable for publication. One very minor addition concerns my previous point 4(c) about the Rac1/Sar1 BiFC puncta: my question was more about total fluorescence rather than number of spots. Of course, it's expected that the ERES number will go up because of the large cell effect, but I was curious as to whether the BiFC approach could be used to interpret an increase in the direct interaction between Sar1 and Rac1 upon stretching, which would further support the authors' claims. Not a big deal, but might be interesting to measure...

As suggested by you, we have now changed a few things in our manuscript to have a better balance between data and conclusions. All changes are highlighted in purple color.
We added a piece of discussion where we clearly state that while we think that the Rac1-Sar1 complex is specific, that future work will likely uncover other endomembrane functions for Rac1 via crosstalk with other small GTPases.

We also changed the initial part of the discussion to make it more balanced.
We changed Figure 3D such that it now shows dots for all individual data points (i.e. cells) where the number of ERES has been counted. The calculated statistics are now significant and therefore supports the data.

Finally, we changed the title of the manuscript to a more conservative one: “Rac1 is a mechanosensitive regulator of ER exit sites”. We think this title is now fully supported by the data and we think because it is short and catchy, that it will appeal to a broader readership.
I hope the changes are satisfactory and that our work is now a candidate for publication in the EMBO Journal.

2nd Revision - Editorial Decision 24th Jun 2022

Dear Hesso,

We have now received re-review reports from all three referees. You have addressed the concerns of the referees satisfactorily. However, I would like you to discuss in the manuscript the points of Referee 2 regarding the specificity of the Sar1-Rac1 interaction.

In addition, there are some remaining editorial points which need to be addressed. Would you therefore please:

incorporate the edits made to the figure legends by Wiley staff in the "Data edited ms file"

add a "Data Availability Section"

include up to five key words

update the Conflict of Interest Section according to the instructions on our website. Please note that Dr Roca-Cusachs is an EMBO Member.

add Amirabbas Parizadeh and Edward Felder to the Author Credit /CrediT section

complete the "Information included in the manuscript?" in the author checklist

consider including ORCID IDs for Drs Roca-Cusachs and Phuyal

update the funding for the project on our submission website

supply EV figures as separate files

include callouts for figures 5D, 6E&F

include an appendix file with a table of contents for submitted files as requested in our instructions to authors

supply a zipped files of movies with their legends

move Table 1 to after the figure legends

move Acknowledgements, Contributions and COI should to before the Reference section

upload EV Figures individually. Figs EV5 and EV6 could be merged into one figure. The legends should be added to the end of the manuscript file, with the heading 'Expanded View Figure Legends'. The Key Resources table could be uploaded as Table EV1

include a panel label for Fig 6A.

We encourage the publication of source data, particularly for electrophoretic gels and blots, with the aim of making primary data more accessible and transparent to the reader. It would be great if you could provide me with a PDF file per figure that contains the original, uncropped and unprocessed scans of all or key gels used in the figures. The PDF files should be labeled with the appropriate figure/panel number, and should have molecular weight markers; further annotation could be useful but is not essential. The PDF files will be published online with the article as supplementary "Source Data" files. Source Data can also include Excel tables to accompany your graphs. We anticipate that their inclusion will make your work more discoverable and useable to scientists in the future.

I would like to request you pay particular attention to the following figures:

in Figures 6D and 8B, the middle lane is too closely cropped. Blots are too closely cropped. Please send the source data and, if possible, include more space around the images

in Figure 7E the blots need to be provided at higher resolution Please also send the source data

provide source data for Figure EV2F, EV6, EV2B and Figure 6E.

We include a synopsis of the paper (see http://emboj.embopress.org/). Please provide me with a general summary statement

and 3-5 bullet points that capture the key findings of the paper. We also need a summary figure for the synopsis. The size should be 550 wide by [200-400] high (pixels). You can also use something from the figures if that is easier.

EMBO Press is an editorially independent publishing platform for the development of EMBO scientific publications.

Thank you again for the opportunity to consider your work for publication. I look forward to your revision.

Best wishes,

William

William Teale, PhD
Editor
The EMBO Journal
w.teale@embojournal.org

Please find attached our revised manuscript that was originally entitled “Mechanical strain stimulates COPII-dependent trafficking via Rac1”.

As suggested by you, we have now changed a few things in our manuscript to have a better balance between data and conclusions. All changes are highlighted in purple color.
We added a piece of discussion where we clearly state that while we think that the Rac1-Sar1 complex is specific, that future work will likely uncover other endomembrane functions for Rac1 via crosstalk with other small GTPases.

We also changed the initial part of the discussion to make it more balanced.

We changed Figure 3D such that it now shows dots for all individual data points (i.e. cells) where the number of ERES has been counted. The calculated statistics are now significant and therefore supports the data.

We changed the title of the manuscript to a more conservative one: “Rac1 is a mechanosensitive regulator of ER exit sites”. We think this title is now fully supported by the data and we think because it is short and catchy, that it will appeal to a broader readership.

Finally, we responded to all other editorial comments.

I hope the changes are satisfactory and that our work is now a candidate for publication in the EMBO Journal

Dear Hesso,

I am pleased to inform you that your manuscript has been accepted for publication in the EMBO Journal.

Congratulations on a really nice study!

Please note that it is EMBO Journal policy for the transcript of the editorial process (containing referee reports and your response letter) to be published as an online supplement to each paper. If you do NOT want this, you will need to inform the Editorial Office via email immediately. More information is available here:
https://www.embopress.org/page/journal/14602075/authorguide#transparentprocess

Your manuscript will be processed for publication in the journal by EMBO Press. Manuscripts in the PDF and electronic editions of The EMBO Journal will be copy edited, and you will be provided with page proofs prior to publication. Please note that supplementary information is not included in the proofs.

You will be contacted by Wiley Author Services to complete licensing and payment information. The required 'Page Charges Authorization Form' is available here: https://www.embopress.org/pb-assets/embo-site/tej_apc.pdf - please download and complete the form and return to embopressproduction@wiley.com

Should you be planning a Press Release on your article, please get in contact with embojournal@wiley.com as early as possible, in order to coordinate publication and release dates.

If you have any questions, please do not hesitate to call or email the Editorial Office. Thank you for your contribution to The EMBO Journal.

Yours sincerely,

William

William Teale, PhD
Editor
The EMBO Journal
w.teale@embojournal.org

** Click here to be directed to your login page: https://emboj.msubmit.net

EMBO Press Author Checklist

Corresponding Author Name: Hessso Farhan	
Journal Submitted to: EMBO Journal	
Manuscript Number: EMBOJ-2022-110596R1	

USEFUL LINKS FOR COMPLETING THIS FORM
The EMBO Journal - Author Guidelines
EMBO Reports - Author Guidelines
Molecular Systems Biology - Author Guidelines
EMBO Molecular Medicine - Author Guidelines

Reporting Checklist for Life Science Articles (updated January 2022)
This checklist is adapted from Materials Design Analysis Reporting (MDAR) Checklist for Authors. MDAR establishes a minimum set of requirements in transparent reporting in the life sciences (see Statement of Task: 10.31222/osf.io/9sm4x). Please follow the journal's guidelines in preparing your manuscript.
Please note that a copy of this checklist will be published alongside your article.

Abridged guidelines for figures
1. Data
The data shown in figures should satisfy the following conditions:
- the data were obtained and processed according to the field's best practice and are presented to reflect the results of the experiments in an accurate and unbiased manner.
- ideally, figure panels should include only measurements that are directly comparable to each other and obtained with the same assay.
- plots include clearly labeled error bars for independent experiments and sample sizes. Unless justified, error bars should not be shown for technical replicates.
- if n<5, the individual data points from each experiment should be plotted. Any statistical test employed should be justified.
- Source Data should be included to report the data underlying figures according to the guidelines set out in the authorship guidelines on Data Presentation.

2. Captions
Each figure caption should contain the following information, for each panel where they are relevant:
- a specification of the experimental system investigated (eg cell line, species name).
- the assay(s) and method(s) used to carry out the reported observations and measurements.
- an explicit mention of the biological and chemical entity(ies) that are being measured.
- an explicit mention of the biological and chemical entity(ies) that are altered/varied/perturbed in a controlled manner.
- the exact sample size (n) for each experimental group/condition, given as a number, not a range;
- a description of the sample collection allowing the reader to understand whether the samples represent technical or biological replicates (including how many animals, litters, cultures, etc.).
- a statement of how many times the experiment shown was independently replicated in the laboratory.
- definitions of statistical methods and measures:
  - common tests, such as t-test (please specify whether paired vs. unpaired), simple χ2 tests, Wilcoxon and Mann-Whitney tests, can be unambiguously identified by name only, but more complex techniques should be described in the methods section;
  - are tests one-sided or two-sided?
  - are there adjustments for multiple comparisons?
  - exact statistical test results, e.g., P values = x but not P values < x;
  - definition of 'center values' as median or average;
  - definition of error bars as s.d. or s.e.m.

Please complete ALL of the questions below.
Select "Not Applicable" only when the requested information is not relevant for your study.

Materials

Newly Created Materials	Information included in the manuscript?	In which section is the information available? (Reagents and Tools Table, Materials and Methods, Figures, Data Availability Section)
New materials and reagents need to be available; do any restrictions apply?	Yes	No resptrictions ally

Antibodies	Information included in the manuscript?	In which section is the information available? (Reagents and Tools Table, Materials and Methods, Figures, Data Availability Section)
For **antibodies** provide the following information:<br>- Commercial antibodies: RRID (if possible) or supplier name, catalogue number and or/clone number<br>- Non-commercial: RRID or citation	Yes	All information is added as a key resurce table

DNA and RNA sequences	Information included in the manuscript?	In which section is the information available? (Reagents and Tools Table, Materials and Methods, Figures, Data Availability Section)
**Short novel DNA or RNA including primers, probes**: provide the sequences.	Yes	all relevant sequences are provided

Cell materials	Information included in the manuscript?	In which section is the information available? (Reagents and Tools Table, Materials and Methods, Figures, Data Availability Section)
**Cell lines:** Provide species information, strain. Provide accession number in repository **OR** supplier name, catalog number, clone number, and/**OR** RRID.	Yes	HeLa cells are HeLaS3 and were purchased from ATCC. PC3 cells are from the Kazaniez lab and were purchased from ATCC
**Primary cultures:** Provide species, strain, sex of origin, genetic modification status.	Not Applicable	NA
Report if the cell lines were recently **authenticated** (e.g., by STR profiling) and tested for mycoplasma contamination.	Yes	Mycoplasma tests are done monthly

Experimental animals	Information included in the manuscript?	In which section is the information available? (Reagents and Tools Table, Materials and Methods, Figures, Data Availability Section)
**Laboratory animals or Model organisms:** Provide species, strain, sex, age, genetic modification status. Provide accession number in repository **OR** supplier name, catalog number, clone number, **OR** RRID.	Not Applicable	NA
**Animal observed in or captured from the field:** Provide species, sex, and age where possible.	Not Applicable	NA
Please detail **housing and husbandry conditions**.	Not Applicable	NA

Plants and microbes	Information included in the manuscript?	In which section is the information available? (Reagents and Tools Table, Materials and Methods, Figures, Data Availability Section)
**Plants:** provide species and strain, ecotype and cultivar where relevant, unique accession number if available, and source (including location for collected wild specimens).	Not Applicable	NA
**Microbes:** provide species and strain, unique accession number if available, and source.	Not Applicable	NA

Human research participants	Information included in the manuscript?	In which section is the information available? (Reagents and Tools Table, Materials and Methods, Figures, Data Availability Section)
If collected and within the bounds of privacy constraints report on age, sex and gender or ethnicity for all study participants.	Not Applicable	NA

Core facilities	Information included in the manuscript?	In which section is the information available? (Reagents and Tools Table, Materials and Methods, Figures, Data Availability Section)
If your work benefited from core facilities, was their service mentioned in the acknowledgments section?	Yes	Acknowledgement section

Design

Study protocol	Information included in the manuscript?	In which section is the information available? (Reagents and Tools Table, Materials and Methods, Figures, Data Availability Section)
If study protocol has been **pre-registered, provide DOI in the manuscript**. For clinical trials, provide the trial registration number **OR** cite DOI.	Not Applicable	NA
Report the **clinical trial registration number** (at ClinicalTrials.gov or equivalent), where applicable.	Not Applicable	NA

Laboratory protocol	Information included in the manuscript?	In which section is the information available? (Reagents and Tools Table, Materials and Methods, Figures, Data Availability Section)
Provide DOI OR other citation details if **external detailed step-by-step protocols** are available.	Not Applicable	NA

Experimental study design and statistics	Information included in the manuscript?	In which section is the information available? (Reagents and Tools Table, Materials and Methods, Figures, Data Availability Section)

Include a statement about **sample size** estimate even if no statistical methods were used.	Not Applicable	
Were any steps taken to minimize the effects of subjective bias when allocating animals/samples to treatment (e.g. **randomization procedure**)? If yes, have they been described?	Not Applicable	
Include a statement about **blinding** even if no blinding was done.	Not Applicable	
Describe **inclusion/exclusion criteria** if samples or animals were excluded from the analysis. Were the criteria pre-established?<br><br>If sample or data points were omitted from analysis, report if this was due to attrition or intentional exclusion and provide justification.	Not Applicable	
For every figure, are **statistical tests** justified as appropriate? Do the data meet the assumptions of the tests (e.g., normal distribution)? Describe any methods used to assess it. Is there an estimate of variation within each group of data? Is the variance similar between the groups that are being statistically compared?	Yes	Mostly t-test was used, either paired or iunpaired, depnding on the experimental design. In Figure 2, where the effect was a Yes/No effect, X2-test was performed

Sample definition and in-laboratory replication	Information included in the manuscript?	In which section is the information available?<br>(Reagents and Tools Table, Materials and Methods, Figures, Data Availability Section)
In the figure legends: state number of times the experiment was **replicated** in laboratory.	Yes	All figures legends contain a clear statement how many times experiments were carried out
In the figure legends: define whether data describe **technical or biological replicates**.	Yes	All replicates mentioned throughout the manuscript are biological replicates.

Ethics

Ethics	Information included in the manuscript?	In which section is the information available?<br>(Reagents and Tools Table, Materials and Methods, Figures, Data Availability Section)
Studies involving **human participants**: State details of **authority granting ethics approval** (IRB or equivalent committee(s), provide reference number for approval.	Not Applicable	
Studies involving **human participants**: Include a statement confirming that **informed consent** was obtained from all subjects and that the experiments conformed to the principles set out in the WMA Declaration of Helsinki and the Department of Health and Human Services Belmont Report.	Not Applicable	
Studies involving **human participants:** For publication of **patient photos**, include a statement confirming that consent to publish was obtained.	Not Applicable	
Studies involving experimental **animals**: State details of **authority granting ethics approval** (IRB or equivalent committee(s), provide reference number for approval. Include a statement of compliance with ethical regulations.	Not Applicable	
Studies involving **specimen and field samples:** State if relevant **permits** obtained, provide details of authority approving study; if none were required, explain why.	Not Applicable	

Dual Use Research of Concern (DURC)	Information included in the manuscript?	In which section is the information available?<br>(Reagents and Tools Table, Materials and Methods, Figures, Data Availability Section)
Could your study fall under dual use research restrictions? Please check biosecurity documents and list of **select agents and toxins** (CDC): https://www.selectagents.gov/sat/list.htm	Not Applicable	No
If you used a select agent, is the security level of the lab appropriate and reported in the manuscript?	Not Applicable	Most regents used in this manuscript are security level1, except where we used lentiviruses which are biosecurity level 2
If a study is subject to dual use research of concern regulations, is the name of the **authority granting approval and reference number** for the regulatory approval provided in the manuscript?	Not Applicable	

Reporting

The MDAR framework recommends adoption of discipline-specific guidelines, established and endorsed through community initiatives. Journals have their own policy about requiring specific guidelines and recommendations to complement MDAR.

Adherence to community standards	Information included in the manuscript?	In which section is the information available?<br>(Reagents and Tools Table, Materials and Methods, Figures, Data Availability Section)
State if relevant guidelines or checklists (e.g., **ICMJE, MIBBI, ARRIVE, PRISMA**) have been followed or provided.	Not Applicable	
For **tumor marker prognostic studies,** we recommend that you follow the **REMARK** reporting guidelines (see link list at top right). See author guidelines, under 'Reporting Guidelines'. Please confirm you have followed these guidelines.	Not Applicable	
For **phase II and III randomized controlled trials**, please refer to the **CONSORT** flow diagram (see link list at top right) and submit the CONSORT checklist (see link list at top right) with your submission. See author guidelines, under 'Reporting Guidelines'. Please confirm you have submitted this list.	Not Applicable	

Data Availability

Data availability	Information included in the manuscript?	In which section is the information available?<br>(Reagents and Tools Table, Materials and Methods, Figures, Data Availability Section)
Have **primary datasets** been deposited according to the journal's guidelines (see 'Data Deposition' section) and the respective accession numbers provided in the Data Availability Section?	Not Applicable	
Were **human clinical and genomic datasets** deposited in a public access-controlled repository in accordance to ethical obligations to the patients and to the applicable consent agreement?	Not Applicable	
Are **computational models** that are central and integral to a study available without restrictions in a machine-readable form? Were the relevant accession numbers or links provided?	Not Applicable	
If publicly available data were reused, provide the respective **data citations in the reference list**.	Not Applicable	
